# MicroRNA target gene prediction model based on input-feature dependency and sample data expansion technique

**DOI:** 10.1371/journal.pcbi.1014402

**Published:** 2026-06-11

**Authors:** Yan Shao, Yazhou Li, Hexin Zhai, Shimin Dong

**Affiliations:** 1 Department of Emergency, The First Hospital of Hebei Medical University, Shijiazhuang, Hebei, China; 2 Department of Emergency, The Fourth Hospital of Hebei Medical University, Shijiazhuang, Hebei, China; 3 Department of Emergency, The Third Hospital of Hebei Medical University, Shijiazhuang, Hebei, China; Xinjiang Technical Institute of Physics and Chemistry, CHINA

## Abstract

Predicting microRNA target genes is essential for understanding their biological functions. This study developed a miRNA target gene prediction model based on input-feature dependency. Features were treated as multiple random variables, with marginal densities estimated using Gaussian mixture models (GMM) and dependencies captured by regular vine (R-vine) copula to derive joint probability density functions. We constructed class-conditional joint densities for positive and negative samples separately using GMM and R-vine copula, then combined these with prior probabilities using Bayes’ rule to obtain posterior probabilities of positive interactions, using a standard 0.5 probability threshold for deterministic prediction. To address insufficient data and class imbalance, hybrid distribution mega-trend diffusion was used to generate virtual samples for data augmentation. Computational validation showed high predictive performance even when only 30% of the training data were used. As proof-of-concept, we experimentally validated one predicted interaction (miR-8485 targeting JAK2) using dual-luciferase, cellular, and animal experiments, confirming the biological relevance of this specific model-generated prediction. These findings provide a valuable tool for understanding miRNA functions and disease mechanisms.

## Author summary

In this study, we developed a new computational model to more accurately predict which genes are regulated by microRNAs—small RNA molecules that play key roles in health and disease. Predicting these targets is difficult because biological data are often limited, imbalanced, and contain complex relationships between features. Our model addresses these challenges by combining two innovations: a probabilistic prediction framework that accounts for dependencies between input features, and a data expansion method that generates realistic synthetic samples to balance the dataset. Computational experiments show that our model performs well even when trained on only 30% of the training data and outperforms existing methods in predictive accuracy. Through laboratory experiments, we validated one prediction—that miR-8485 targets the *JAK2* gene—serving as a proof-of-concept demonstration that the model can generate biologically-plausible hypotheses. Our findings provide researchers with a promising tool for uncovering microRNA functions, which can help advance our understanding of diseases and support the development of new therapies.

## Introduction

MicroRNAs (miRNAs) are a class of endogenous, non-coding, single-stranded RNA molecules of 20–25 bases long. The miRNAs are involved in various physiological processes, including cell differentiation, hormone secretion, lipid metabolism, apoptosis, growth, and development, as well as various pathological processes, including lung cancer, leukemia, diabetes, colon cancer, and viral infections [1]. Studies on miRNAs enhance our understanding of complex regulatory networks in organisms, providing theoretical insights into cellular behavior and disease pathogenesis, as well as potential practical applications in disease diagnosis, treatment, and prevention [2]. To date, researchers have identified a large number of miRNAs; however, the functions and mechanisms of action of most of these miRNAs are unclear. Consequently, identifying the target genes regulated by miRNAs is crucial, highlighting the urgent need to develop efficient target gene recognition algorithms [3].

The earliest methods for target gene prediction were based on biological experiments, primarily including western blotting, reporter gene assays, DNA microarrays, immunoprecipitation, and protein mass spectrometry [4]. Advances in miRNA-related knowledge and the development of target gene prediction technology have considerably improved the prediction of miRNA target genes using bioinformatics algorithms. Target gene prediction methods are based on sequence rules or machine learning [5]. Sequence rule-based methods use statistical patterns of known miRNAs and their target genes as recognition features for prediction. Common models based on this approach include miRanda, TargetScan, and RNAhybrid [6]. However, these methods rely on biophysical models that are highly dependent on the experience of the designer or user expertise, introducing strong subjectivity. Concurrently, the relationship between miRNAs and target genes is complex and high-dimensional, which often leads to substantial errors in predictions even when advanced algorithms are applied [7].

Conversely, machine learning-based approaches use statistical models to automatically discover recognition rules from training datasets to obtain predictive results. Compared with biological experimental methods and sequence rule-based approaches, machine learning can achieve more accurate predictions. Accordingly, since the advent of TargetBoost, the first target gene prediction method based on machine learning, in 2005, a large number of target gene prediction methods based on machine learning have been developed [8]. With the development of deep neural network (DNN) technology, an increasing number of researchers have used DNN models to predict target genes [9]. For instance, Yadalam et al. [10] proposed a gradient boosting prediction method based on weighted co-expression and differential gene expression analyses. Uthayopas et al. [11] proposed the PRIMITI method for target gene prediction. This method integrates CLIP-seq and gene expression data and uses XGBoost with multiple features to predict functional miRNA-binding sites and their inhibitory activity on mRNA. Xie et al. [12] used a vector projection similarity-based method to predict miRNA-disease associations. Their results indicated that leave-one-out cross-validation (LOOCV) and five-fold cross-validation (CV) experiments demonstrated good performance of the proposed method. More recently, several advanced computational approaches have been developed for related biomedical prediction tasks. Zhao et al. [13] proposed a heterogeneous information network learning model with neighborhood-level structural representation for predicting long non-coding RNA (lncRNA)–miRNA interactions, demonstrating the power of integrating multi-source biological data.

In the context of drug repositioning, Zhao et al. [14] introduced a geometric deep learning framework that leverages attention mechanisms over heterogeneous information networks to predict drug–disease associations. More recently, Li et al. [15] developed a sequence-based deep learning model combining convolutional neural networks with attention mechanisms to predict HIV-1 protease cleavage sites, effectively handling imbalanced data through biased support vector machines. The DNN model has strong adaptability and feature extraction capability and can complete prediction tasks more effectively; however, its application is limited by two major problems. The first problem is the low accuracy of predictions. Target gene prediction can be classified into deterministic and probabilistic approaches according to different prediction results. The result obtained from deterministic prediction is a specific outcome with a more intuitive form (e.g., whether the target gene corresponds to a miRNA) [16]. The result of probabilistic prediction is a probability distribution that can provide uncertainty analysis for the prediction [17]. Although many existing machine learning models for target gene prediction can produce probabilistic outputs (e.g., through softmax layers or calibrated probability estimates), these approaches typically operate within a discriminative framework that does not explicitly model feature dependencies. For instance, the degree of seed-region base-sequence matching and miRNA–mRNA dimer thermodynamic stability are two commonly-used prediction characteristics. In general, if the base-matching degree is high, the thermodynamic stability is greater; thus, the two are positively dependent [18]. Therefore, to increase prediction accuracy, one can establish a probabilistic prediction model that considers dependencies among all input features, combined with deterministic prediction, to obtain more complete prediction results and accurately identify target genes regulated by miRNAs.

Target gene prediction requires a large number of similar types of miRNA-positive and miRNA-negative target gene samples to complete the modeling task, and the proportion of these two types of samples in the dataset should be relatively balanced [19]. However, in many cases, the modeling process cannot identify a sufficient number of similar types of sample data, resulting in a serious imbalance in the proportion of sample data in the modeling process. For instance, existing gene databases have more positive samples than negative samples, which can affect prediction accuracy. In this regard, sample dataset expansion techniques provide a solution for insufficient sample data and unequal sample distributions in prediction models.

Commonly-used dataset expansion methods include interpolation, noise injection, data sampling, and virtual sample generation [20]. Of these, mega-trend diffusion (MTD), a virtual sample generation technique, is one of the most commonly-used and effective methods [21]. The traditional MTD method typically assumes that the original samples follow a specific probability distribution and generates virtual samples based on this assumed distribution. However, in practical applications, the original samples often fail to conform strictly to a predefined theoretical distribution. This discrepancy between model assumptions and real-world conditions leads to substantial errors in the virtual samples produced using conventional MTD approaches [22,23]. Consequently, Dong et al. [24] proposed a dual-distribution MTD technique. This approach innovatively uses a normal distribution to model the original sample intervals while utilizing a uniform distribution to construct virtual sample intervals, thereby achieving effective dataset expansion. However, gene data often exhibit complex multimodal distribution characteristics, and this dual-distribution modeling approach has certain limitations when dealing with such intricate distribution patterns. Therefore, improving the data representation capability and establishing a hybrid distribution (HD) data expansion mechanism is necessary.

Therefore, in the current study, we constructed a miRNA target gene prediction model based on input-feature dependency. First, 18 features of the target gene were considered as inputs representing multiple random variables, and a class-conditional generative modeling strategy was used. Gaussian mixture models and R-vine copulas were used to estimate the joint density of features separately for positive and negative samples. These class-conditional densities were then integrated with prior probabilities using Bayes’ theorem to compute the posterior probability of the true interaction. The posterior probabilities we obtained originated from explicitly modeling the class-conditional joint distributions of features. This generative framework enabled us to capture complex feature dependencies that are often overlooked in conventional approaches. A decision threshold of 0.5 was applied to the posterior probability to obtain binary classifications. Finally, HD–MTD was used to construct virtual samples to effectively enhance the prediction accuracy of both parametric and non-parametric models and to address the problem of insufficient total sample data and imbalanced proportions of negative and positive samples.

## Result

### Data set description

The miRNA target gene prediction dataset was extracted from TarBase v8.0. The dataset comprised data from five higher mammals: *Homo sapiens*, *Rattus norvegicus*, *Mus musculus*, *Bos taurus*, and *Ovis aries*. After removing the polycyclic data from the miRNA–mRNA dimer structure, 831 positive miRNA target genes and 306 negative miRNA target genes were identified. The dataset was randomly split into training (70%) and test (30%) sets, stratified by class label to preserve the original class distribution. The test set was held out entirely during model development and hyperparameter tuning and was used only for final performance evaluation.

### Prediction criteria

The prediction results were divided into four types: true positive (TP), true negative (TN), false positive (FP), and false negative (FN). These four outcomes correspond to correctly-predicted positive samples, correctly-predicted negative samples, incorrectly-predicted positive samples, and incorrectly-predicted negative samples. The five commonly-used criteria for target gene prediction were:


$$\begin{array}{c} \text{Accuracy }=\frac{TP\;+\;TN}{TP\;+\;TN\;+\;FP\;+\;FN}\;\times \;\text{1}\text{0}\text{0}\% \end{array}$$ (1)

$$\begin{array}{c} \text{Specificity }=\frac{TN}{TN\;+\;FP}\;\times \;\text{1}\text{0}\text{0}\% \end{array}$$ (2)

$$\begin{array}{c} \text{Precision }=\frac{TP}{TP\;+\;Fp}\;\times \;\text{1}\text{0}\text{0}\% \end{array}$$ (3)

$$\begin{array}{c} \text{Recall }=\text{ Sensitivity }=\frac{TP}{TP\;+\;FN}\;\times \;\text{1}\text{0}\text{0}\% \end{array}$$ (4)

An additional criterion, the F1 score, was introduced to balance precision and recall.


$$\begin{array}{c} \text{F1 score }=\;\frac{(\text{1}\;+\;\beta^{\text{2}})\;recall\;\times \;precision}{\beta^{\text{2}}\times \;recall\;+\;precision} \end{array}$$ (5)

where β∈[0,1].

Based on Equation (5), a higher F1 value indicates better recall and precision, and thus a more accurate prediction.

A receiver operating characteristic (ROC) curve is a graphical representation that visually demonstrates the performance of a model. In the current study, the area under the ROC curve (AUC) was used to evaluate the model’s performance. The horizontal and vertical axes of the ROC curve represent the true positive rate (TPR) and false positive rate (FPR), respectively, where TPR = Sensitivity, and FPR = 1 − Specificity.

### Statistical analysis

To assess the statistical significance of the performance improvements achieved by our proposed model, we conducted paired bootstrap tests at the instance level with 10,000 resamples to compare the F1 scores and AUC values between our model and each baseline model. Specifically, for each comparison between the proposed model and a baseline model, we resampled the test set instances with replacement (maintaining the original sample size) and recomputed the F1 score and AUC for both models on each bootstrap sample. The *p*-value for each comparison was calculated as the proportion of bootstrap samples in which the performance difference (proposed model minus baseline model) was ≤ 0 (two-sided test). This paired approach ensured that both models were evaluated on identical bootstrap samples, providing a fair comparison. The 95% confidence intervals (CIs) for all evaluation metrics were computed using the percentile bootstrap method (10,000 resamples) at the instance level.

Given that our proposed model was compared against five baseline models (GMM without dependency; single Gaussian model (SGM) considering dependency; Weibull distribution (WD); the convolutional neural network model from the miRBench framework, miRBench-CNN [25]; and the hybrid architecture combining an autoencoder and a convolutional neural network, Hybrid AE-CNN) we applied the Bonferroni correction for multiple comparisons to control the family-wise error rate. The significance threshold was adjusted to α = 0.05/ 5 = 0.01. Thus, *p*-values < 0.01 were considered statistically significant. All reported *p*-values in S5 Table are raw *p*-values; asterisks indicate significance after Bonferroni correction (***p* < 0.001, **p* < 0.01, *p* < 0.05 before correction, with only *p* < 0.01 considered significant after correction).

### Target gene feature extraction and recognition rules

In total, 90 miRNA target gene features were extracted in this study, including seed-region hydrogen bond count, elemental ratio, and continuous matching characteristics; thermodynamic features; seed-region conservation features; context sequence features; target gene accessibility penalty features; miRNA sequence 2-mer features; and binding structure features. The decision function of the proposed prediction model was:


$$\begin{array}{c} S=\sum_{i=\text{1}}^{k}\omega_{i}x_{i}\;+\;b \end{array}$$ (6)

where $x_{i}$ and $\omega_{i}$ are the value and weight of the *i*-th feature, respectively; *k* is the total number of features, and b is the bias term. If *S* > 0, the sample is classified as a positive target gene; otherwise, it is classified as a negative target gene.

The normalized 90-dimensional feature vector of each sample and its label (positive/negative) were used as input data, and the optimal weight vector and bias were obtained by solving the following convex quadratic programming problem:


$$\begin{array}{c} \left\{ \begin{array}{c} {min}_{\omega ,b}\frac{\text{1}}{\text{2}}{\left\|\omega_{i}\right\|}^{\text{2}}+C\sum_{i=\text{1}}^{n}\varepsilon_{i}\\ \text{s}.\text{t}.\;y_{i}({\omega_{i}}^{T}x_{i}+b)\geq \text{1}-\varepsilon_{i},\varepsilon_{i}\geq \text{0} \end{array}\right.\; \end{array}$$ (7)

where $\left\|\omega_{i}\right\|$ represents the norm of the weight vector, ρ≥0 denotes the margin variable, C is the penalty parameter, and $\varepsilon_{i}\geq \text{0}$ are the slack variables.

To obtain the optimal weight vector, *ω*, we solved the convex quadratic programming problem defined in Eq. (7) using a linear Support Vector Machine (SVM). The model was implemented using the LinearSVC class from the scikit-learn library in Python, with the liblinear solver selected. A linear kernel was used, consistent with the linear decision function shown in Eq. (6). The penalty parameter, *C*, was optimized using five-fold CV on the training set. We conducted a grid search over *C* ∈ {0.01, 0.1, 1, 10, 100} and selected the value that maximized the average G-means score across the validation folds. The optimal *C* value was found to be 1.

The most important *k* features directly associated with the biological mechanism of miRNA targeting were selected from all 90 characteristics as input features for the prediction model, whereas the remaining characteristics were treated as redundant information. This approach effectively reduced computational costs.

The feature effectiveness measure, $\gamma_{i}$, was used to calculate the importance of each feature:


$$\begin{array}{c} \gamma_{i}={\left(\frac{\omega^{*}(i)}{\left\|\omega^{*}\right\|}\right)}^{\text{2}} \end{array}$$ (8)

where $\omega^{*}(i)$ is the *i*-th element of $\omega_{i}$.

The 90 features were sorted based on their $\gamma_{i}$ values in descending order, and the top *k* features with the highest $\gamma_{i}$ values were selected.

The value of *k* was determined using the G-means (∈[0,1]), which is the geometric mean of TP and TN; a value closer to 1 indicated better model performance. When the number of features was 18, G-means approached a peak value, indicating optimal performance. Increasing the number of features did not substantially improve the G-means but increased computational cost. Therefore, *k* = 18 was selected as the optimal feature set size. The values and weights of the input features selected for this study are presented in Table 1.

**Table 1:** Values and weights of each input feature.

Feature	Value (${𝐱}_{i}$)	Weight ($\omega_{i}$)
Sm_6mer	$x_{\text{1}}=\left\{ \begin{array}{c} @l\text{6},\;if\;Sm\_\text{6}me\\ \text{5},\;if\;Sm\_\text{5}me \end{array}\right.\;$	+0.85
Rgs_match	$x_{\text{2}}=\left\{ \begin{array}{c} @l\text{1},\;if\;G-U\;\\ \text{2},\;if\;A-U\;\\ \text{3},\;if\;G-C\; \end{array}\right.\;$	+0.72
Rgs_energy	$x_{\text{3}}={\Delta G}_{seed}=MFE({miRNA}_{pos\text{2}-\text{8}}:{mRNA}_{seed\;region})$ Minimum free energy (MFE) of the seed region (positions 2–8) duplex, calculated using RNAfold. More negative values indicate greater thermodynamic stability of the seed pairing.	-0.62
Acc_energy	$x_{\text{4}}={\Delta G}_{accessibility}=MFE({mRNA}_{local\;context})$ Energy required to make the target site accessible for miRNA binding, estimated as the MFE of the local mRNA secondary structure surrounding the target site, calculated using RNAfold	-0.58
Rgt_energy	$x_{\text{5}}={\Delta G}_{total}=MFE({miRNA}_{full\;length}:{mRNA}_{full\;target\;site})$ Minimum free energy of the full-length miRNA–mRNA duplex (including both seed and non-seed regions), calculated using RNAfold	-0.41
Rgt_match	$x_{\text{6}}=j$ j is the number of nucleotide matches in the non-seed region	+0.35
Sm_7mer_m8	$x_{\text{7}}=\left\{ \begin{array}{c} @l\text{1},\text{ perfect match at position}s\;\text{2}-\text{8}\\ \text{0},\;otherwise \end{array}\right.\;$	+0.42
Sm_7mer_m1	$x_{\text{8}}=\left\{ \begin{array}{c} @c\text{1},\;z_{\text{1}}-{\;z}_{\text{7}}\;complementary\;pairing\;with\;target\;gene\\ \text{\textasciitilde{}\textasciitilde{}\textasciitilde{}\textasciitilde{}0},\;otherwise \end{array}\right.\;$	+0.38
Sm_7mer_A1	$x_{\text{9}}=\left\{ \begin{array}{c} @c\text{1},\text{ perfect match at position}s\;\text{2}-\text{8}\text{ and }\prime A^{\prime }\;opposite\;miRNA\\ \;position\;\text{1}\\ \text{0},\;otherwise \end{array}\;\right.\;$	+0.36
Consv_seed	$x_{\text{10}}=\tau_{\text{1}}$ $\tau_{\text{1}}\in [\text{0},\text{1}]\;$ is the PhyloP score	+0.38
2mer1	$x_{\text{11}}=\left\{ \begin{array}{c} @c\text{1},\;z_{\text{1}}-z_{\text{2}}\;complementary\;pairing\;with\;target\;gene\\ \text{0},\;otherwise \end{array}\right.\;$	+0.22
Consv_3cntxt	$x_{\text{12}}=\tau_{\text{2}}$ $\tau_{\text{2}}\in [\text{0},\text{1}]\;$ is the PhyloP score	+0.18
Rgs_mismatch	$x_{\text{13}}=N$ N is the number of seed region mismatches	-0.45
2mer12	$x_{\text{14}}=\left\{ \begin{array}{c} @c\text{1},\;z_{\text{12}}-z_{\text{13}}\;complementary\;pairing\;with\;target\;gene\\ \text{0},\;otherwise \end{array}\right.\;$	+0.15
Consv_5cntxt	$x_{\text{15}}=\tau_{\text{3}}$ $\tau_{\text{3}}\in [\text{0},\text{1}]\;$ is the PhyloP score	+0.12
Nt1	$x_{\text{16}}=\vartheta$ The value of ϑ represents the base type at the first position of the target gene: ϑ = 1 for A, ϑ = 2 for U, ϑ = 3 for G, and ϑ = 4 for C.	+0.10
2mer7	$x_{\text{17}}=\left\{ \begin{array}{c} @c\text{1},\;z_{\text{7}}-z_{\text{8}}\;complementary\;pairing\;with\;target\;gene\\ \text{0},\;otherwise \end{array}\right.\;$	+0.08
2mer6	$x_{\text{18}}=\left\{ \begin{array}{c} @c\text{1},\;z_{\text{6}}-z_{\text{7}}\;complementary\;pairing\;with\;target\;gene\\ \text{0},\;otherwise \end{array}\right.\;$	+0.06

### Biological rationale for the selected 18 features

The 18 selected features can be categorized into four biologically-relevant groups based on their functional roles in miRNA–mRNA targeting (Table 1).

First, seed region features (Sm_6mer, Sm_7mer_m8, Sm_7mer_m1, Sm_7mer_A1, Rgs_match, Rgs_mismatch) capture the critical base-pairing between miRNA positions 2–8 and the mRNA target. The seed region is well-established as the primary determinant of miRNA target recognition [18], and mismatches in this region severely impair binding efficiency.

Second, thermodynamic features (Rgs_energy, Acc_energy, Rgt_energy) reflect the binding stability and accessibility of the target site. Rgs_energy quantifies seed region duplex stability, Acc_energy measures the energy required to unfold local mRNA secondary structures, and Rgt_energy represents total binding affinity. These features collectively determine whether a target site is accessible and thermodynamically-favorable for miRNA binding [6].

Third, conservation features (Consv_seed, Consv_3cntxt, Consv_5cntxt) indicate evolutionary pressure on the target site. PhyloP scores measure nucleotide-level conservation across species; higher conservation suggests functional importance and has been shown to correlate with genuine miRNA targeting [7].

Fourth, contextual features (2mer1, 2mer6, 2mer7, 2mer12, Nt1, Rgt_match) capture flanking sequence effects and non-seed region contributions. The 2-mer features represent dinucleotide pairing patterns outside the seed region that modulate binding specificity [12]. Nt1 records the nucleotide type at the first position of the target gene, and Rgt_match quantifies non-seed region complementarity. These contextual factors are increasingly recognized as important modulators of miRNA targeting efficiency [25].

Together, these 18 features comprehensively represent the multi-faceted mechanisms of miRNA–mRNA interaction, including sequence complementarity, thermodynamic stability, evolutionary conservation, and contextual modulation.

### Feature number selection using G-means

**Fig 1:**
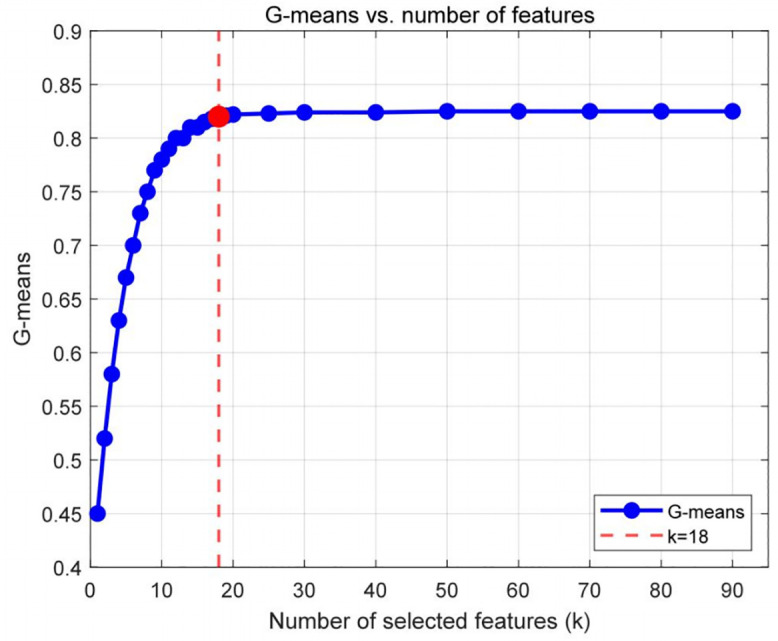
Feature number selection based on G-means.

To determine the optimal number of features (k), we sorted all 90 features in descending order of $\gamma_{\text{i}}$ and evaluated the G-means (geometric mean of sensitivity and specificity) on the validation set as k increased from 1 to 90. The G-means increased rapidly from k = 1 to k = 18 (from 0.45 to 0.820), reaching a clear plateau at k = 18 (Fig 1). Beyond k = 18, the G-means improved only marginally (ΔG-means < 0.005), increasing from 0.820 at k = 18 to 0.825 at k = 90. Therefore, k = 18 was selected as the optimal feature set size, balancing predictive performance and computational cost.

### Dependency analysis

**Fig 2:**
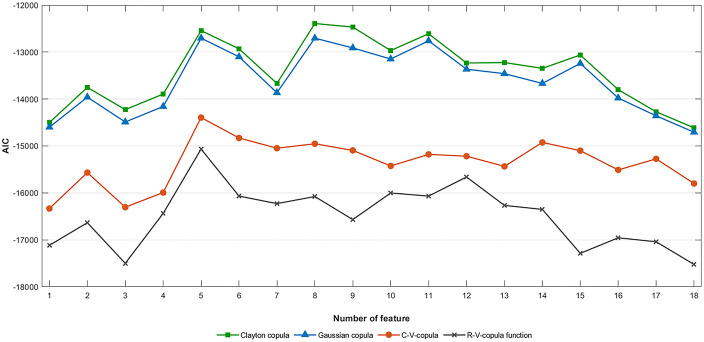
Akaike information criterion (AIC) comparison results of different models.

Clayton, Gaussian, and canonical vine copulas were used for comparison to demonstrate the superiority of the R-V-copula function. The Akaike information criterion (AIC) was used to evaluate the dependency-fitting ability of the different copula models. The smaller the AIC value, the stronger the model’s fitting capability. The AIC comparison results for the different copula functions are shown in Fig 2.

The Gaussian and Clayton copulas exhibited the highest AIC values, indicating the poorest ability to describe dependencies. This is primarily because both copulas could not adequately capture dependencies among high-dimensional multivariate variables, and their performance decreased substantially as dimensionality increased. In the current study, the input consisted of 18 different feature types, forming high-dimensional multivariate data; therefore, the results obtained using these two copula functions were poor.

The canonical vine copula, as a vine-structure copula, was more suitable for modeling dependencies among high-dimensional variables than the Gaussian and Clayton copulas and showed relatively good fitting performance. However, the C-vine copula had structural limitations and could not fully capture the complexity of genetic data. In contrast, the R-V-copula, as one of the most general vine structures, provided strong fitting capability for high-dimensional multivariate variables and did not suffer structural limitations, thereby yielding the best results.

### Performance test of the proposed prediction model

Three benchmark models (GMM without dependency, single Gaussian model (SGM) considering dependency, and the Weibull distribution (WD)) and two state-of-the-art models (the convolutional neural network model from the miRBench framework (miRBench-CNN) [25] and the hybrid architecture combining an autoencoder and a convolutional neural network (Hybrid AE-CNN) [26]) were used to evaluate the effectiveness of the proposed prediction model (GMM with dependency).

To ensure fair and reproducible comparisons with state-of-the-art models, all baseline models were evaluated under identical experimental conditions. Specifically:

(1) Data partitioning: All models were trained and tested on the same stratified 70% –30% train–test split of the dataset, with the test set kept untouched until final evaluation. The same random seed (42) was used to ensure reproducibility.
(2) Input features: All baseline models used the identical set of 18 selected features listed in Table 1. For deep learning models (miRBench-CNN and Hybrid AE-CNN) that originally accepted different feature formats, we adapted their input layers to accept the 18-dimensional feature vector while preserving their core architectural designs as described in the original publications [25,26].
(3) Hyperparameter selection: For miRBench-CNN and Hybrid AE-CNN, hyperparameters were optimized using five-fold CV on the training set only, following the same procedure used for our model. The optimal configurations were: for miRBench-CNN, we used the default architecture from miRBench but reduced the input dimension to 18; for Hybrid AE-CNN, we retained the autoencoder latent dimension of 32 and CNN kernel sizes of 3 and 5, as originally reported. All other hyperparameters (learning rate: 0.001, batch size: 32, epochs: 100 with early stopping) were kept identical across deep learning models.

**Fig 3:**
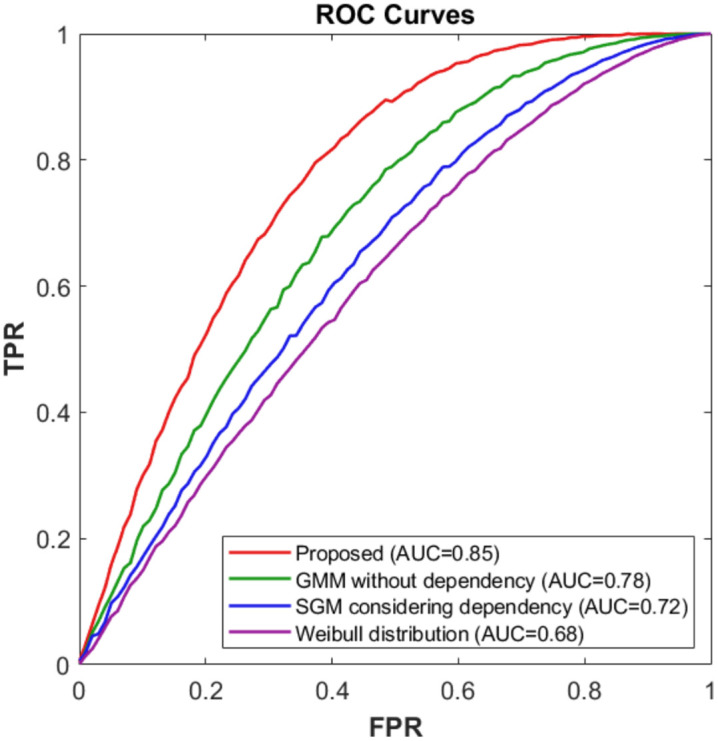
Receiver operating characteristic (ROC) curves of different models. TPR, true positive rate; FPR, false positive rate.

The comparison results and ROC curves of the different models are presented in Table 2 and Fig 3. The results demonstrated that:

**Table 2:** Comparison of results under different criteria.

Model	F1 score	Accuracy	Specificity	Precision	Sensitivity	AUC
Proposed	0.8139 [0.792, 0.836]	0.8253 [0.804, 0.847]	0.8431 [0.822, 0.864]	0.7953 [0.771, 0.819]	0.7867 [0.763, 0.810]	0.85 [0.82,0.88]
GMM without dependency	0.7417 [0.714, 0.769]	0.74659 [0.720, 0.773]	0.7653 [0.739, 0.792]	0.7217 [0.693, 0.750]	0.7196 [0.691, 0.748]	0.78 [0.75, 0.81]
SGM considering dependency	0.6941 [0.667, 0.721]	0.7077 [0.681, 0.734]	0.7175 [0.691, 0.744]	0.6819 [0.653, 0.711]	0.6723 [0.644, 0.701]	0.72 [0.69, 0.75]
WD	0.6441 [0.618, 0.670]	0.6575 [0.632, 0.683]	0.6752 [0.649, 0.701]	0.6232 [0.596, 0.650]	0.6157 [0.588, 0.643]	0.68 [0.65, 0.71]
miRBench-CNN	0.8127 [0.791, 0.834]	0.8256 [0.804, 0.847]	0.8321 [0.810, 0.854]	0.7831 [0.759, 0.807]	0.7761 [0.752, 0.800]	0.83 [0.80, 0.86]
Hybrid AE-CNN	0.8137 [0.792, 0.835]	0.8145 [0.793, 0.836]	0.8226 [0.801, 0.844]	0.7765 [0.752, 0.801]	0.7621 [0.737, 0.787]	0.81 [0.78, 0.84]

All baseline models were retrained and evaluated under identical experimental conditions: the same 70%/30% train–test split, the same 18 input features (Table 1), and consistent five-fold cross-validation on the training set for hyperparameter selection. Values in brackets indicate 95% confidence intervals calculated from 10 independent runs with different random seeds. Statistical significance compared to the proposed model: *p < 0.05, **p < 0.01, ***p < 0.001 (paired bootstrap test with 10,000 resamples). Full experimental details are provided in the ‘Benchmark model comparison settings’ subsection of the Methods section.

(1) Of the probabilistic models, GMM outperformed both SGM and WD across all evaluation metrics, confirming the advantage of mixture modeling over single-distribution assumptions for capturing complex feature distributions in miRNA target data.
(2) Incorporating feature dependencies via R-vine copula improved performance, as evidenced by the comparison between the full model and GMM without dependency, highlighting the importance of modeling inter-feature correlations.
(3) When compared with state-of-the-art deep learning methods under identical experimental conditions (same data split, same 18 features, and consistent CV protocol), the proposed model achieved competitive performance. The model attained an F1 score of 0.8139 and an AUC of 0.85, which are comparable to those of miRBench-CNN (F1 = 0.8127, AUC = 0.83) and Hybrid AE-CNN (F1 = 0.8137, AUC = 0.81) (Table 2). All values reported in Table 2 were obtained by retraining each baseline model on our training set and evaluating on our test set, rather than being directly cited from prior publications. This ensures a fair comparison across all methods.

We conducted paired bootstrap tests (10,000 resamples) to assess whether the performance differences between our proposed model and each baseline model were statistically significant. Our model substantially outperformed all baseline models (GMM without dependency, SGM considering dependency, WD, miRBench-CNN, and Hybrid AE-CNN) in terms of both F1 score and AUC (all *P* values < 0.05) (S5 Table). These results confirm that the observed improvements were not due to random chance and demonstrate the robustness of our approach.

### Performance evaluation under different data sizes

To systematically evaluate the performance of the proposed model under limited data conditions and to assess the effectiveness of HD-MTD, we conducted experiments using 30%, 50%, and 100% of the training set (which originally comprised 70% of the entire dataset). The test set remained unchanged throughout. For each data size condition, we applied three data expansion techniques (HD-MTD, DD-MTD, and traditional MTD) to generate virtual negative samples, balancing the positive-to-negative ratio to 1:1. The experimental protocol, detailed in the Methods section, involved 10 repeated runs with different random seeds to ensure statistical robustness. The mean F1 scores and 95% confidence intervals are presented in Table 3. For the experiments evaluating data expansion techniques, we adopted the following rigorous protocol to prevent data leakage (Table 3):

**Table 3:** Performance evaluation using different data sizes.

Model	F1 score (100% data)	F1 score (50% data)	F1 score (30% data)
Proposed model with HD-MTD	0.8809 [0.86,0.90]	0.8102 [0.79,0.83]	0.7931 [0.77,0.81]
Proposed model with DD-MTD	0.8509 [0.83,0.87]	0.7802 [0.76,0.80]	0.7531 [0.73,0.77]
Proposed model with traditional MTD	0.8312 [0.81,0.85]	0.7362 [0.71,0.76]	0.7013 [0.68,0.72]
Proposed model without data expansion technique	0.8139 [0.79,0.84]	0.7219 [0.70,0.74]	0.6732 [0.65,0.70]

Values in brackets indicate 95% confidence intervals calculated from 10 independent runs with different random seeds. Paired bootstrap tests (10,000 resamples) confirmed that HD-MTD outperforms all other data expansion techniques across all data sizes (p < 0.05 for all comparisons). These results demonstrate that the performance gains achieved by HD-MTD are statistically significant and robust across different data availability scenarios.

(1) Data partitioning: For each experimental condition (using 100%, 50%, or 30% of the total data), the original dataset was first randomly split into training (70%) and test (30%) sets using stratified sampling to preserve the original class distribution.
(2) Data expansion: Different types of MTD were applied exclusively to the training set to generate virtual negative samples. The target ratio after expansion was set to 1:1 (positive:negative), meaning that negative samples were generated until the number of negative samples in the training set equaled the number of positive samples.
(3) Model training and evaluation: The model was trained on the expanded training set (containing both original and synthetic samples) and evaluated on the pristine test set (containing only original samples, with no synthetic data). This ensured that performance metrics reflected the model’s generalization ability on real-world data.
(4) Robustness check: The entire procedure (splitting–expansion–training–evaluation) was repeated 10 times with different random seeds, and the average F1 scores are reported in Table 3.

In the presence of sufficient sample data (i.e., when 100% of the total data were used), all data expansion techniques were able to generate virtual negative samples to balance the proportion of positive and negative samples, thereby enhancing prediction accuracy. Compared with traditional MTD and DD-MTD techniques, HD-MTD performed dataset expansion more effectively. When the number of samples was insufficient (i.e., when 30% or 50% of the total data were used), the prediction accuracy of the models decreased as the amount of data decreased. The HD-MTD technique effectively alleviated this decline in prediction accuracy because it used HDs to describe the data.

When trained on only 30% of the training data, the model with HD-MTD achieved an F1 score of 0.7931 (95% CI: [0.77, 0.81]), which was comparable to the performance of the model trained on 100% of the data without expansion (F1 = 0.8139). Statistical analysis confirmed that the difference between these two conditions was not significant (*p* > 0.05, paired bootstrap test), supporting the claim that the proposed approach performed effectively even with substantially-reduced training data. This finding highlights the practical utility of HD-MTD in scenarios where labeled data are scarce.

To assess whether HD-MTD generated virtual samples that preserved biologically-meaningful feature distributions, we conducted comprehensive quantitative comparisons between the original negative samples (n = 306) and the virtual negative samples generated using HD-MTD.

Three complementary aspects of distributional fidelity were evaluated (S4 Table):

(1) Univariate distribution similarity: The Kullback–Leibler (KL) divergence between original and synthetic distributions averaged 0.039 (range: 0.012–0.087) across all 18 features, with values close to zero indicating high similarity. Kolmogorov–Smirnov (KS) tests yielded *p* > 0.05 for all features, indicating no statistically significant differences between the original and synthetic samples.
(2) Moment preservation: Key statistical moments—including mean, variance, skewness, and kurtosis—showed relative differences below 5% for all 18 features, confirming that the synthetic data faithfully reproduced the central tendency, dispersion, and shape characteristics of the original data.
(3) Feature correlation preservation: To verify that interdependencies among features (e.g., the positive correlation between seed region matching and thermodynamic stability) were maintained, we compared the pairwise correlation matrices of original and synthetic samples. The average absolute difference in correlation coefficients was 0.042 ± 0.031 (S4 Table), indicating that feature correlation structures were well preserved without introducing artificial relationships.

Detailed results for each of the 18 features, including KL divergence, KS test *p*-values, moment comparisons, and feature correlation preservation metrics, are provided in S4 Table.

Together, these quantitative analyses demonstrate that HD-MTD generated virtual negative samples that closely approximated the statistical properties, distributional characteristics, and feature correlation patterns of real biological data. The improved predictive performance achieved with augmented data (Table 3) can therefore be attributed to effective data supplementation rather than synthetic data bias.

### Experimental validation: A proof-of-concept case study

To demonstrate the biological relevance of predictions generated by the model, we selected a top-ranked prediction—miR-8485 targeting JAK2—for experimental validation. It is important to note that this single-case validation is intended as a proof-of-concept to illustrate that the model can generate biologically plausible hypotheses, rather than as a statistical validation of the model’s overall predictive accuracy. The following dual-luciferase, cellular, and animal experiments were performed to evaluate the biological validity of this specific predicted interaction.

### Dual-luciferase assay

We performed a luciferase assay using miR-8485 and its corresponding target gene, Janus kinase 2 (JAK2) (predicted by the model in the previous section), to validate the effectiveness of the prediction model proposed in this study. The reagents used are listed in S1 Table.

**Fig 4:**
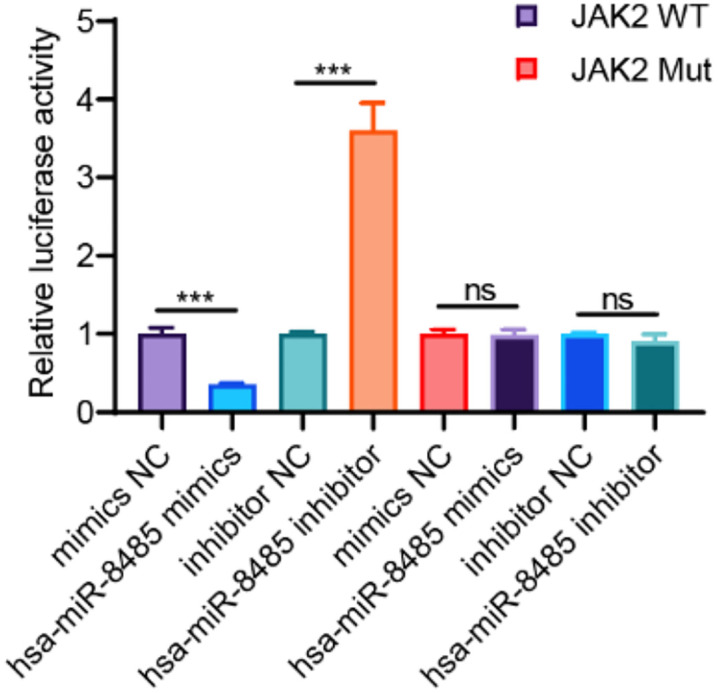
Dual-luciferase assay results.

An endotoxin-free mini plasmid extraction kit was used to perform endotoxin-free extraction of the constructed recombinant reporter gene plasmid pmirGLO-JAK2-WT/pmirGLO-JAK2-Mut. After resuscitation, HL-1 cells were seeded evenly into 24-well plates. Transfection was initiated when the cells reached approximately 80% confluency. The cell transfection procedure was performed according to pre-designed experimental groups (JAK2 gene was divided into wild-type (WT) and mutant-type, with miR-8485 treated with mimics and inhibitor, respectively). After 24 h of transfection, the cells were digested and collected. Cell lysis was performed using the dual-luciferase reporter assay kit. The lysed samples were analyzed using GloMax 20/20 luminometer to measure firefly and Renilla luciferase activities separately. Firefly luciferase/Renilla luciferase ratio was calculated, and statistical analysis was performed using GraphPad Prism software. The results are shown in Fig 4.

Treatment with miR-8485 mimics significantly reduced luciferase activity in the JAK2 WT group, demonstrating that miR-8485 specifically binds to the 3′UTR of JAK2 to suppress its expression. Conversely, treatment with the miR-8485 inhibitor increased the luciferase activity in the JAK2 WT group, confirming its negative regulatory effect on JAK2. Notably, these regulatory effects were abolished in the JAK2 mutant-type group, demonstrating that miR-8485–mediated regulation depends on specific binding to the JAK2 3′UTR sequence. These results indicate that JAK2 is a direct target of miR-8485, providing initial proof-of-concept support for this specific prediction generated by our model.

### Cellular experiments

**Fig 5:**
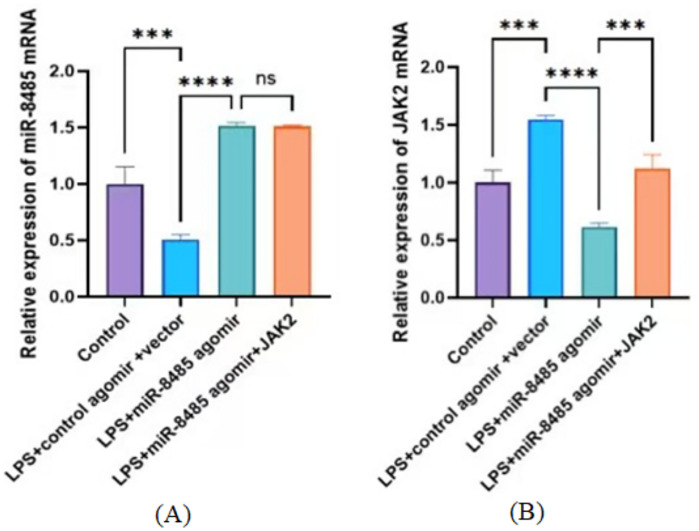
Comparison of (a) miR-8485 and (b) JAK2 expression in cell experiments.

Cellular experiments were performed to verify the validity of the proposed prediction model by investigating the targeted regulation of JAK2 by miR-8485. The main instruments and reagents are shown in S2 and S3 Tables. Cell culture was performed by thawing and passaging, which served as the basis for subsequent experiments. The cells were divided into groups as shown in Table 4. After treatment, RNA extraction, reverse transcription, and qPCR were performed sequentially, and the results are shown in Fig 5.

**Table 4:** Treatment groups for cellular experiments.

Group name	Treatment steps
Control Group (Group 1)	Cells were cultured in complete medium for 48 h, with no transfection and lipopolysaccharide (LPS) stimulation.
LPS + Control Agomir + Vector Group (Group 2)	Complete media was replaced with serum-free DMEM 2 h before transfection. The transfection complex, comprising Control Agomir (final concentration, 50 nM) + Vector (final concentration, 2 μg/well) + Exfect 2000 Transfection Reagent (Reagent:Nucleic acid = 1:2), was prepared and incubated at room temperature for 20 min and then added to the wells. After 24 h of transfection, the medium was replaced with complete medium containing 1 μg/mL LPS and cultured for an additional 24 h (total 48 h).
LPS + miR-8485 Agomir Group (Group 3)	Complete media was replaced with serum-free DMEM 2 h before transfection. Cells were transfected with miR-8485 Agomir (final concentration 50 nM). After 24 h of transfection, 1 μg/mL LPS was added, followed by culturing for 24 h.
LPS + miR-8485 Agomir + JAK2 Group (Group 4)	Complete media was replaced with serum-free DMEM 2 h before transfection. The transfection complex, comprising miR-8485 Agomir (final concentration, 50 nM) + JAK2 Overexpression Vector (final concentration, 2 μg/well) + Exfect 2000, was prepared and incubated at room temperature for 20 min and then added to the well. After 24 h of transfection, 1 μg/mL LPS was added to the media followed by culturing for 24 h.

As shown in Fig 5a, compared with the control group, miR-8485 expression was significantly decreased in the LPS-induced inflammatory model (Group 2), whereas it was significantly increased in the miR-8485 agomir (Group 3) group. No significant difference in miR-8485 expression was observed between Group 4 (JAK2 complementation) and Group 3, indicating successful overexpression of miR-8485, which was unaffected by JAK2 complementation.

As shown in Fig 5b, JAK2 expression was significantly higher in Group 2 than that in the control group. However, after transfection with miR-8485 agomir (Group 3), JAK2 expression significantly decreased. JAK2 expression in Group 4 (JAK2 complementation) was significantly higher than that in Group 3, indicating that miR-8485 overexpression inhibited JAK2 expression, and this inhibition was reversed by JAK2 complementation. The results in Fig 5 collectively verify the targeted regulation of JAK2 by miR-8485, consistent with this prediction from our model.

### Verification using animal experiments

Animal experiments were performed to further verify the validity of the proposed prediction model by investigating the targeted regulation of JAK2 by miR-8485. Mice aged 8–12 weeks and weighing 20–25 g (C57BL/6J, male, clean grade) were purchased from Beijing SiPeiFu Biotech Co., Ltd. (Certificate No.: 110324241105032918, License No.: SYXK(Ji)2018–001). Animals were housed in an environment with constant temperature (22 ± 2°C) and humidity (60%), and a 12-h light/dark cycle, with free access to food and water and were acclimatized for 1 week. All animal experiments in this study complied with the Animal Experiment Ethics Standards of Hebei Medical University.)

**Fig 6:**
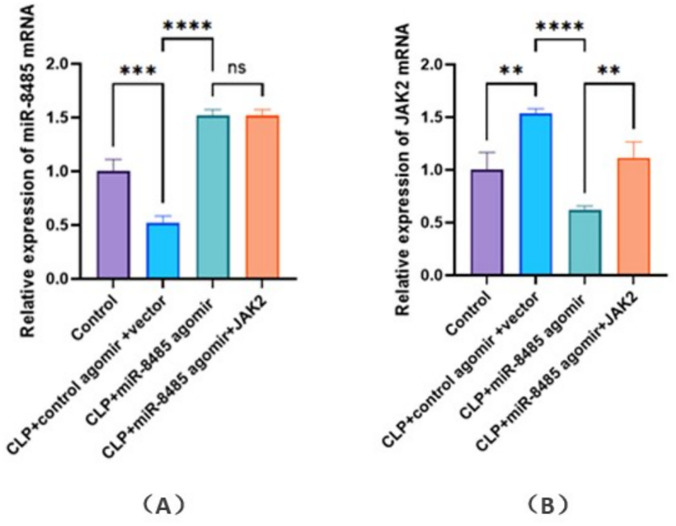
Comparison of (a) miR-8485 and (b) JAK2 expression in animal experiments.

The mice were divided into groups as shown in Table 5. Results of total RNA extraction from myocardial tissue, reverse transcription reaction, and qPCR detection, are shown in Fig 6.

**Table 5:** Treatment groups for the animal experiments.

Group name	Treatment steps
Control Group (Group 1)	Mice underwent anesthesia, laparotomy–cecum repositioning–abdominal closure only (without CLP), followed by tail vein injection of an equivalent volume of sterile PBS, and received standard postoperative warming care. Samples were collected 24 h later.
Cecal Ligation and Puncture (CLP) + Control Agomir + Vector Group (Group 2)	Within 1 h post-CLP, mice underwent tail vein injection of control agomir (8 optical density units) + empty vector (2 μg/mouse). Thereafter, mice received postoperative warming during recovery and had free access to food and water. Samples were collected 24 h later.
CLP + miR-8485 Agomir Group (Group 3)	Within 1 h post-CLP, mice underwent tail vein injection of miR-8485 agomir (8 optical density units). Thereafter, mice received standard postoperative care. Serum and myocardial tissue were collected under anesthesia 24 h later.
CLP + miR-8485 Agomir + JAK2 Group (Group 4)	Within 1 h post-CLP, mice underwent tail vein co-injection of miR-8485 agomir (8 optical density units) + JAK2 overexpression vector (2 μg/mouse). Thereafter, mice received postoperative warming care. Samples were collected for detection 24 h later.

As shown in Fig 6a, after establishing the sepsis mouse model (Group 2), miR-8485 expression was significantly decreased compared with that in the control group. Upon further addition of the agomir (Group 3), miR-8485 expression was significantly increased. After JAK2 rescue (Group 4), miR-8485 expression showed no significant change compared with that in Group 3. This confirmed that miR-8485 could be successfully overexpressed in mice, and this overexpression was not affected by subsequent JAK2 rescue. As shown in Fig 6b, after establishing the sepsis mouse model (Group 2), JAK2 expression was significantly increased compared with that in the control group. Upon further addition of the miR-8485 agomir (Group 3), JAK2 expression was significantly decreased. After JAK2 rescue (Group 4), JAK2 expression was significantly increased compared with that in Group 3. This demonstrated that overexpressed miR-8485 could inhibit JAK2 expression, and JAK2 supplementation could reverse this inhibition. Together, the results presented in Fig 6 provide further evidence that miR-8485 targets and regulates JAK2, supporting the specific prediction generated by our model.

## Discussion

In this study, we developed a class-conditional generative model for miRNA target gene prediction that differs fundamentally from conventional discriminative approaches and uses an HD-based sample expansion technique. Although many existing methods (e.g., DNNs, XGBoost) can produce probabilistic outputs, they typically do so by learning decision boundaries rather than explicitly modeling the joint distribution of features. In contrast, our approach explicitly learns the class-conditional joint distribution through a combination of GMM and R-V-copula. This generative framework offers unique advantages:

(1) it explicitly models dependencies among features rather than assuming independence or learning boundaries without capturing underlying distributions;
(2) it provides principled uncertainty quantification through posterior probabilities derived from Bayes’ rule; and
(3) HD-MTD enables the construction of virtual samples to effectively enhance prediction accuracy and address the problem of limited sample size.

The use of a GMM for marginal probability density estimation allowed us to capture complex, multimodal distributions of miRNA target features without relying on strong prior assumptions. This represents a substantial advantage over traditional parametric models, which often fail to accurately represent the underlying data structure inherent in biological systems [27]. The GMM-based approach provided more accurate probabilistic predictions, as evidenced by higher F1 scores and AUC values than those of other models, underscoring the importance of flexible modeling of feature distributions.

Furthermore, we introduced the R-V-copula to model dependencies among the 18 selected features. The R-V-copula outperformed other dependency modeling techniques in terms of AIC values, confirming its superior capability for handling high-dimensional dependencies. This is particularly important in miRNA target prediction, where features such as seed region matching and thermodynamic stability are often correlated and collectively influence targeting efficacy, an aspect often overlooked in conventional models.

Another key innovation of this study is the application of HD–MTD for sample expansion. By clustering samples based on statistical attributes and assigning optimal distribution types to each cluster, HD-MTD effectively generated virtual samples that improved model performance, particularly under limited data conditions. This approach mitigated the common issue of sample imbalance and enhanced the robustness of both parametric and non-parametric models, addressing a critical need in biomedical data science where labeled data are often scarce [28].

Experimental validation through dual-luciferase assays, cellular experiments, and animal studies confirmed that miR-8485 directly targeted JAK2, consistent with the model’s prediction. The marked reduction in luciferase activity and JAK2 expression following miR-8485 overexpression, with the reversal of this effect upon JAK2 supplementation, provides biological evidence supporting this specific predicted interaction (Figs 4–6), underscoring the utility of computational predictions in guiding experimental research as a hypothesis-generation tool.

Despite these advancements, this study had certain limitations. The model’s performance depended heavily on the quality and completeness of the feature set, and the current feature selection process, although optimized, may still overlook biologically-relevant attributes. Although HD-MTD improved sample balance, the generated virtual samples were based on statistical distributions and may not fully capture biological variability, a limitation common to virtual data generation methods [29].

All performance improvements reported in this study were supported by rigorous statistical testing. The 95% confidence intervals computed for all evaluation metrics (Tables 2 and 3) demonstrate the stability of the results across different data splits and random initializations. Paired bootstrap tests confirmed that the proposed model outperformed all baseline probabilistic models (*p* < 0.001) and achieved improvements over state-of-the-art deep learning methods (*p* < 0.05) (S5 Table). These results provide strong evidence that the observed performance gains were not due to chance.

Experimental validation was conducted on only one predicted interaction (miR-8485 targeting JAK2). Although dual-luciferase, cellular, and animal experiments confirmed the biological relevance of this prediction, a single positive case does not provide a statistical estimate of the model’s false-positive rate or its generalizability. Therefore, these findings should be interpreted as a proof-of-concept demonstration of the model’s utility for hypothesis generation rather than a comprehensive validation of its predictive accuracy. The training dataset (TarBase v8.0) was also relatively small and imbalanced. Although HD-MTD mitigated this issue, the synthetic samples may not fully capture the complexity of biological data.

Future work should address these limitations through several complementary directions. First, large-scale experimental validation on a randomly-selected cohort of predictions (e.g., 20–30 interactions) is needed to rigorously assess the model’s false- positive rate. Second, incorporating additional training data from high-throughput technologies and integrating more diverse biological features—such as spatial structure data or miRNA–mRNA interaction kinetics—could enhance prediction accuracy [30]. Third, exploring alternative synthetic sample generation strategies that better preserve biologically-meaningful distributions may improve the handling of imbalanced data.

## Conclusion

We established a robust miRNA target prediction framework that effectively integrated probabilistic modeling, feature dependency, and sample expansion. The proposed model demonstrated strong performance on benchmark datasets, and experimental validation of one predicted interaction (miR-8485 targeting JAK2) confirmed the biological relevance of this specific model-generated prediction, illustrating its utility for hypothesis generation. This framework provides a valuable tool for guiding experimental validation and functional studies on miRNAs, contributing to the growing arsenal of computational approaches for precision medicine research.

## Materials and methods

### Ethics

This study followed the 3R principles. The experimental protocol was approved by the Institutional Ethics Committee of the Fourth Hospital of Hebei Medical University (No. 20240032) and Ethics Committee of the Third Hospital of Hebei Medical University (No. 20221182), in compliance with GB/T 35892–2018. Surgical and procedural interventions were performed under anesthesia and analgesia with humane endpoints, and euthanasia was carried out in accordance with the AVMA Guidelines at the conclusion of the experiment. As this study involved only animal experiments (C57BL/6J mice) and commercial cell lines (HL-1 cells), and did not include any human participants, no informed consent was required.

### GMM

The GMM has multiple centers and can easily fit multi-peak or multi-valley distributions, allowing approximation of almost all distribution types. Therefore, although the GMM is a parametric model, it does not require strong prior assumptions, which effectively addresses the limitations of parametric models. Additionally, the GMM has a closed-form solution, providing strong interpretability of the data [31]. Therefore, in this study, probabilistic prediction was performed using the GMM.

The GMM PDF can be represented as follows [32]:


$$\begin{array}{c} P(x|\Theta )=\sum_{k=\text{1}}^{M}\gamma_{k}N(x|\vartheta_{k}) \end{array}$$ (9)

$$\begin{array}{c} N\left(x|\vartheta_{k}\right)=\frac{\text{1}}{{\left(\text{2}\pi \right)}^{d/\text{2}}{\left|L_{k}\right|}^{\text{1}/\text{2}}}exp[-\frac{\text{1}}{\text{2}}{\left(x-\mu_{k}\right)}^{T}{L_{k}}^{-\text{1}}(x-\mu_{k})] \end{array}$$ (10)

The dataset is denoted as *D* = $\left\{x_{\text{1}},x_{\text{2}},\cdots ,x_{n}\right\}$, where *M* is the number of components, and *d* is the dimensionality. $\mu_{k}$, $L_{k}$, and $\gamma_{k}$ denoted the mean, covariance matrix, and mixing coefficient of the k-th component, respectively. Accordingly, $\vartheta_{k}=(\mu_{k},L_{k})$. The number of components for the GMM was selected from {1, 2, 3, 4, 5} based on the average log-likelihood in cross-validation.

GMM parameter estimation can be represented as follows:

The GMM parameters ($\mu_{k}$, $L_{k}$, and $\gamma_{k}$) were estimated using the Expectation-Maximization (EM) algorithm, which can be divided into two steps:

E-step：Compute the posterior probability that each data point $x_{i}$ belongs to component k:


$$\begin{array}{c} \gamma_{ik}=\frac{\gamma_{k}N(x_{i}|\vartheta_{k})}{\sum_{j=\text{1}}^{M}\gamma_{j}N(x_{i}|\vartheta_{j})} \end{array}$$ (11)

M-step: Update the parameters using the responsibilities:


$$\begin{array}{c} \overset{new}{\underset{k}{\gamma }}=\frac{\text{1}}{n}\sum_{j=\text{1}}^{M}\gamma_{ik},\overset{new}{\underset{k}{\mu }}=\frac{\sum_{i=\text{1}}^{n}\gamma_{ik}\cdot x_{i}}{\sum_{i=\text{1}}^{n}\gamma_{ik}},\;\overset{new}{\underset{k}{L}}=\frac{\sum_{i=\text{1}}^{n}\gamma_{ik}(x_{i}-\overset{new}{\underset{k}{\mu }}){\left(x_{i}-\overset{new}{\underset{k}{\mu }}\right)}^{T}}{\sum_{i=\text{1}}^{n}\gamma_{ik}} \end{array}$$ (12)

The EM algorithm was initialized using k-means clustering and iterated until the change in log-likelihood was less than ${\text{1}\text{0}}^{-\text{4}}$ or a maximum of 100 iterations was reached. To avoid local optima, the EM algorithm was run 10 times with different random initializations, and the solution with the highest log-likelihood was selected.

### R-V-copula

The copula function is the most commonly used tool to establish dependencies between marginal PDFs [33]. To construct the predictive model, we adopted a class-conditional generative approach. Specifically, we built two separate R-V-copula models: one for the positive samples (miRNA target genes) and one for the negative samples (non-target genes). For each class cϵ{pos,neg}, we used a GMM to estimate the marginal PDFs of the 18 features, and an R-V-copula to capture dependencies among these features. This yielded two class-conditional joint PDFs, f(T|pos), and f(T|neg), where $T=(T_{\text{1}},T_{\text{2}},\cdots T_{\text{1}\text{8}})$ is the feature vector of a given miRNA-target gene pair.

(1) Construction of the basic structure of a regular vine

A nested tree $Tr=({Tr}_{\text{1}},\;{Tr}_{\text{2}},\ldots ,{Tr}_{d})$ was constructed, wherein each ${Tr}_{i}$ with *N* nodes had *E* edges, and *d* is the dimension of the input variables. Each edge is expressed as {*y, z|M*}, where *M* is the conditioning set and {*y, z, y ≠ z}* is the conditioned set. All elements, including *y, z,* and *M*, are nodes. In the first tree, *M* is an empty set.

Each edge *e =* {*y(e), z(e) | M(e)*} in the${\;T}_{i}$ tree depends on two edges,${\;e}_{\text{1}}=\{\text{y}(e_{\text{1}}),\;z(e_{\text{1}})|M(e_{\text{1}})\}$ and $e_{\text{2}}=\{y(e_{\text{2}}),\;z(e_{\text{2}})|M(e_{\text{2}})\}$, where $e_{\text{1}}$ and $e_{\text{2}}$ share a common node. Therefore, the relationship between the edges is as follows:


$$\begin{array}{c} M(e)=\text{S}(e_{\text{1}})\cap \text{S}(e_{\text{2}}) \end{array}$$ (13)

$$\begin{array}{c} e=\{y(e),\;z(e)|M(e)\} \end{array}$$ (14)

where S(·)={y(·), z(·)|M(·)} is the set of edges. For each edge e, under the given conditioning, the corresponding bivariate copula density functions are $P_{y(e),\;z(e)\;|\;M(e)}$.

Thus, $D_{M(e)}$, $D_{y(e)}$, and $D_{z(e)}$ represent the subsets of the *d*-dimensional vector *D* corresponding to the elements *M(e)*, *y*(*e*), and *z*(*e*)*,* respectively.

(2) Modeling the R-V-copula function

To avoid information leakage, all vine structure selection and copula family determination were performed within each cross-validation fold using only the training data.

First, the edge set ({*y, z|M*}) of each tree was determined. Considering both the accuracy and computational complexity of the model, edges with high dependency in the first few trees were selected. Dependency was measured using Kendall’s rank correlation coefficient [34]. We used the maximum spanning tree algorithm to select edges with the largest sum of absolute empirical Kendall rank correlation coefficients.

Second, we considered the choice of the binary copula corresponding to each edge. Additionally, we calculated the AIC for all candidate binary copula families [35]. The binary copula function with the smallest AIC was considered to have the best goodness-of-fit and was therefore selected for each edge. The AIC was obtained using the following formula:


$$\begin{array}{c} AIC=\text{2}d-\text{2}In(\hat{L}) \end{array}$$ (15)

where *d* is the number of parameters and $\hat{L}$ is the maximized likelihood of the sample set. The higher the value of *d*, more complex the model.

For copula parameter estimation, for each selected bivariate copula family, the copula parameters were estimated using maximum likelihood estimation (MLE). Given a set of observations$\overset{\text{n}}{\underset{\text{i}=\text{1}}{{\{(F}_{\text{c}}(T_{y(e)}|T_{M(e)}),\;F_{\text{c}}(T_{z(e)}|T_{M(e)})\}}}$for the two variables, the log-likelihood function is:


$$\begin{array}{c} L(\theta_{y(e),\;z(e)\;|\;M(e)})=\sum_{i=\text{1}}^{n}logP_{y(e),\;z(e)\;|\;M(e)}({\text{F}}_{\text{c}}({T_{y(e)}}^{\left(\text{i}\right)}|{T_{M(e)}}^{\left(i\right)},{\text{F}}_{\text{c}}({T_{z(e)}}^{\left(\text{i}\right)}|{T_{M(e)}}^{\left(i\right)});\theta_{y(e),\;z(e)\;|\;M(e)}) \end{array}$$ (16)

Where $\theta_{y(e),\;z(e)\;|\;M(e)}$ denotes the copula parameters for edge *e*. The MLE $\hat{\theta }$ was obtained by numerical optimization using the sequential quadratic programming (SQP) algorithm implemented in the VineCopula package.

For samples belonging to a given class cϵ{pos,neg}, the class-conditional joint probability density function of the 18 features can be expressed as:


$$\begin{array}{c} f_{c}(T_{\text{1}},\;T_{\text{2}},\;\cdots ,T_{d})=[\prod_{i=\text{1}}^{d}f_{c,i}(T_{i})]\cdot [\prod_{t=\text{1}}^{d-\text{1}}\prod_{e\in E_{t}}P_{y(e),\;z(e)\;|\;M(e)}(F_{c}(T_{y(e)}|T_{M(e)}),{\text{F}}_{\text{c}}(T_{z(e)}|T_{M(e)}))] \end{array}$$ (17)

$$\begin{array}{c} {\text{F}}_{\text{c}}({\text{T}}_{\text{y}(\text{e})}|{\text{T}}_{\text{M}(\text{e})})=\frac{\partial {\text{P}}_{y(\text{e}),\;z(\text{e})\;|\text{ M}(\text{e})}({\text{F}}_{\text{c}}({\text{T}}_{\text{y}(\text{e})}|{\text{T}}_{\text{D}(\text{e})}),{\text{F}}_{\text{c}}({\text{T}}_{\text{z}(\text{e})}|{\text{T}}_{\text{D}(\text{e})}))}{\partial {\text{F}}_{\text{c}}({\text{T}}_{\text{z}(\text{e})}|{\text{T}}_{\text{D}(\text{e})})} \end{array}$$ (18)

Where $f_{c,i}(T_{i})$ is the marginal density of the *i*-th feature for class c, and $F_{c}(\cdot |\cdot )$ are the class-specific conditional distribution functions. $P_{y(e),\;z(e)\;|\;M(e)}$is the bivariate copula density for the pair (y(e), z(e)) conditioned on the set M(e).

Finally, prediction was performed using Bayes’ rule. For a new instance with a feature vector $T=(T_{\text{1}},T_{\text{2}},\cdots T_{\text{1}\text{8}})$, the posterior probability of being a positive target was computed using:


$$\begin{array}{c} P(pos|T)=\frac{f(T|pos)\cdot P(pos)}{f(T|pos)\cdot P(pos)+f(T|neg)\cdot P(neg)} \end{array}$$ (19)

Where, f(T|pos) and f(T|neg)  are the class-conditional joint densities estimated by the GMM and R-V-copula models, and P(pos) and P(neg) are the prior probabilities estimated from the training data. Based on the class distribution in our dataset (831 positive and 306 negative samples), the priors were set to P(pos)=831/1137 ≈ 0.731, and P(neg)=306/1137 ≈ 0.269.

The final deterministic prediction was obtained by thresholding the posterior probability at 0.5: if P(pos|T)>0.5, the instance was classified as a positive target; otherwise, it was classified as negative.

### Sample data expansion technique

In this study, we used HD-MTD for sample data expansion, which was conducted as follows:

#### Step 1: Selection of data distribution features.

Based on statistical theory, the data distribution types were determined by central tendency, dispersion, and distribution shape. Eight key statistical attributes were selected to characterize the distribution types: kurtosis, skewness, mean, median, mode, range, variance, and standard deviation.

#### Step 2: Clustering of original data.

Based on the above eight attributes, the k-means algorithm was used to cluster the original data by grouping samples with similar attributes into the same cluster. We assumed that the original dataset contained *N* samples $X=\{X_{\text{1}},X_{\text{2}},\ldots ,X_{N}\}$, each with eight attributes. First, min–max normalization was conducted to map the values to the interval [0,1] using the following formula:


$$\begin{array}{c} {\text{x}}_{\text{new}}=\frac{{\text{x}}_{\text{i}}\;-\;{\text{x}}_{\text{min}}}{{\text{x}}_{\text{max}}\;-\;{\text{x}}_{\text{min}}} \end{array}$$ (20)

where $x_{min}$ is the minimum value of the sample data, and $x_{max}$ is the maximum value.

Second, the cluster centers were initialized. *M* initial cluster centers were selected: $C=\{C_{\text{1}},C_{\text{2}},\ldots ,C_{M}\}$.

The Euclidean distance to each cluster center was calculated for each sample $X_{i}$:


$$\begin{array}{c} \text{dis}({\text{X}}_{\text{i}},{\text{C}}_{\text{j}})=\sqrt{\sum_{\text{t}=\text{1}}^{\text{n}}\;({\text{X}}_{\text{it}}-{\text{C}}_{\text{jt}}{)}^{\text{2}}} \end{array}$$ (21)

where $C_{j}$ denotes the *j*-th cluster center, $X_{it}$ denotes the *t*-th attribute of the *i*-th sample, and $C_{jt}$ denotes the *t*-th attribute of the *j*-th cluster center.

The optimal number of clusters, *M*, was determined using the elbow method based on the within-cluster sum of squares (WCSS). Specifically, k-means clustering for *M* from 2–10 was conducted and the WCSS was plotted against the number of clusters. The optimal *M* was selected as the point where the decrease in WCSS began to diminish, forming an ‘elbow’ in the curve. The optimal number of clusters was determined to be *M* = 3.

Considering the eight input attributes of the training set as inputs to the k-means algorithm, the original data were all clustered into *M* clusters.

To prevent information leakage, the optimal number of clusters (M) was determined independently within each training fold using the elbow method, based solely on the training data of that fold. The validation fold and test set were never used in this process. Across all five CV folds and 10 independent runs with different random seeds, M was consistently equal to three, indicating that the clustering structure was stable and not sensitive to the specific composition of the training data.

#### Step 3: Assignment of corresponding distribution types to each cluster.

For each cluster obtained in Step 2, we aimed to select the optimal distribution that best characterized its data. To achieve this, we used log-likelihood as the goodness-of-fit measure. For a given cluster, we considered a set of candidate distribution families, including, but not limited to the normal, Weibull, gamma, and lognormal distributions. For each candidate distribution, we first estimated its parameters, θ, via maximum likelihood estimation based on the data points in that cluster.

The log-likelihood (LL) of the cluster data under the candidate distribution was computed as:


$$\begin{array}{c} LL=\sum_{j=\text{1}}^{N_{cluster}}log\;f(x_{j}|\theta ) \end{array}$$ (22)

where $f(x_{j}|\theta )$ is the PDF of the candidate distribution with the estimated parameters, θ. The LL quantifies how likely it is to observe the given cluster data under a specific distribution; a higher value indicates a better fit. For each cluster, we selected the candidate distribution that yielded the maximum log-likelihood as its optimal distribution type. This process was performed independently for each cluster. Crucially, to prevent data leakage, this entire selection process—including parameter estimation and log-likelihood calculation—was conducted within each CV fold using only the training data. The validation folds remained untouched during this step.

#### Step 4: Generation of virtual data.

The MTD method was used to generate virtual samples based on the corresponding distribution types assigned to different clusters, as described previously [23,24].

To prevent data leakage, HD-MTD was applied only to the training data. During CV, virtual samples were generated exclusively from each training fold, whereas the corresponding validation fold was left untouched. For final model evaluation, HD-MTD was applied only to the full training set (70% of the data), and the test set (30%) was never used in the generation process.

### Computational complexity

For GMM: For a dataset with *n* samples and *d* features, the expectation maximization (EM) algorithm for GMM had a complexity of O(n·d·δ·I), where δ is the number of mixture components and *I* is the number of EM iterations. In our experiments (*n* = 1137, *d* = 18, δ≤5 and I≈20−50), GMM estimation for a single feature was completed within seconds. Since this process was repeated for each of the 18 features and for both positive and negative classes, the total GMM estimation time was 2–3 minutes.

For R-V-copula: The vine structure selection using the maximum spanning tree algorithm had a complexity of $O(d^{\text{2}}logd)$ for each tree level. For *d* = 18, this step was negligible. Copula parameter estimation for each edge required O(n) operations for likelihood evaluation and numerical optimization. With approximately d(d−1)/2=153 edges in total, the entire vine construction and estimation process took 2–3 minutes on a standard desktop computer.

## Supporting information

S1 Table Main reagents used in the dual-luciferase assay. (DOCX)

S2 Table Main instruments used in cellular experiments. (DOCX)

S3 Table Main reagents used in cellular experiments. (DOCX)

S4 Table Comparison of statistical properties between original negative samples and synthetic samples generated using HD-MTD for all 18 features. (DOCX)

S5 Table Statistical significance of performance comparisons between the proposed and baseline models. (DOCX)

S1 File S1 Fig. Recombinant plasmid map of pmirGLO-JAK2-Mut. **S2 Fig.** Recombinant plasmid map of pmirGLO-JAK2-WT. **S3 Fig.** Relative luciferase activity. **S4 Fig.** Dual-luciferase reporter assay results for miR-8485 inhibitor. **S5 Fig.** miR-8485 mimic and inhibitor sequences. **S6 Fig.** Dual-luciferase reporter assay results for miR-8485 mimics. **S7 Fig.** Binding site of hsa-miR-8485 on JAK2 3′UTR. **S8 Fig.** JAK2 reporter gene detection report. **S1 Protocol.** JAK2 reporter gene plasmid construction protocol.
(ZIP)
