# Supplementary material for: MicroRNA target gene prediction model based on input-feature dependency and sample data expansion technique
Source: PLoS Comput Biol. 2026 Jun 11;22(6):e1014402. doi: 10.1371/journal.pcbi.1014402 (PMC13258019; doi:10.1371/journal.pcbi.1014402)
Supplement: S1 Table — (DOCX) [file pcbi.1014402.s001.docx]

Table S1. Main reagents used in dual luciferase assay.

| Reagent name | Manufacturer | Catalog number |
| --- | --- | --- |
| Dual Luciferase Reporter Assay Kit | Vazyme | DL101-01 |
| Exfect 2000 Transfection Reagent | Vazyme | T202-01 |
| Endotoxin-free Miniprep Plasmid Kit | Tiangen | DP118 |
| GloMax 20/20 Luminometer | Promega | / |
| Biosafety cabinet | Shangguang | SW-CJ-2D |
| Cell culture incubator | Suzhou Jiemei | CI-191C |
| Benchtop Low-Speed Centrifuge | Hunan Xiangyi | L400 |
| SpectraMax iD5 Multi-Mode Microplate Reader | Shanghai Shanpu | Read Max 1200 |
