# Supplementary material for: MicroRNA target gene prediction model based on input-feature dependency and sample data expansion technique
Source: PLoS Comput Biol. 2026 Jun 11;22(6):e1014402. doi: 10.1371/journal.pcbi.1014402 (PMC13258019; doi:10.1371/journal.pcbi.1014402)
Supplement: S2 Table — (DOCX) [file pcbi.1014402.s002.docx]

Table S2. Main instruments used in cellular experiments.

| Instrument name | Instrument model | Brand |
| --- | --- | --- |
| Pipette | —— | Eppendorf |
| Inverted Fluorescence Microscope | ICX41 | Ningbo Sunny |
| Ultra-clean Workbench | SW-CJ-2D | Shangguang |
| CO₂ Cell Incubator | CI-191C | Suzhou Jiemei |
| Desktop Low-speed Centrifuge | L400 | Hunan Xiangyi |
| Countess 3 | AMQAX2000 | Invitrogen |
| Countess Cell Counting Chamber Slides | C10283 | Invitrogen |
| 96-well Plate |  | KIRGEN |
| T25 Cell Culture Flask |  | KIRGEN |
| PCR Gene Gradient Amplifier | TC100-G | Dragon Lab |
| High-speed Refrigerated Centrifuge | D1524R | Dragon Lab |
| Digital Constant Temperature Water Bath | HH-2 | Changzhou Yuexin |
| NanoDro One/OneC Micro UV-Vis Spectrophotometer | 840-317400 | Thermo |
| StepOnePlus Real-time Fluorescent Quantitative PCR System | 4376600 | Thermo |
