# Supplementary material for: MicroRNA target gene prediction model based on input-feature dependency and sample data expansion technique
Source: PLoS Comput Biol. 2026 Jun 11;22(6):e1014402. doi: 10.1371/journal.pcbi.1014402 (PMC13258019; doi:10.1371/journal.pcbi.1014402)
Supplement: S3 Table — (DOCX) [file pcbi.1014402.s003.docx]

Table S3. Main reagents used in cellular experiments.

| Reagent name | Manufacturer | Catalog number |
| --- | --- | --- |
| DMEM Medium | Saiweier | G4515 |
| Fetal Bovine Serum (FBS) | Yikesai | FCS500 |
| Trypsin-EDTA Digestive Solution | Solarbio | T1300 |
| PBS | Saiweier Biology | G4202-500ML |
| Cell Counting Kit-8 | Solarbio | CA1210 |
| Trypan Blue stain 0.4% | Invitrogen | T1028 |
| Penicillin-Streptomycin Mixture (100×) | Solarbio | P1400 |
| TRNzol Universal Reagent | TIANGEN | DP424 |
| Anhydrous Ethanol | Baishi Chemical, Tianjin | 2023-11-15 |
| Isopropanol | Baishi Chemical, Tianjin | 2023-11-15 |
| Chloroform Substitute | ECOTOP, Guangzhou | ES-8522-100mL |
| FastKing One-Step Genomic DNA Removal cDNA First-Strand Synthesis Premix Reagent | TIANGEN | KR118 |
| SuperReal Fluorescent Quantitative Premix Reagent (Enhanced Version) | TIANGEN | FP205 |
| Enzyme-free Sterile Water | Solarbio | R1600 |
| RNAase Inhibitor | Solarbio | R8061 |
