# Supplementary material for: MicroRNA target gene prediction model based on input-feature dependency and sample data expansion technique
Source: PLoS Comput Biol. 2026 Jun 11;22(6):e1014402. doi: 10.1371/journal.pcbi.1014402 (PMC13258019; doi:10.1371/journal.pcbi.1014402)
Supplement: S4 Table — (DOCX) [file pcbi.1014402.s004.docx]

****Table S4. Comparison of statistical properties between original negative samples and synthetic samples generated by HD-MTD for all 18 features.****

| Feature | Original Mean | Virtual Mean | Rel. Diff (%) | Original Var | Virtual Var | Skewness (Orig) | Skewness (Vir) | Kurtosis (Orig) | Kurtosis (Vir) | KL Div | KS p-value |
| --- | --- | --- | --- | --- | --- | --- | --- | --- | --- | --- | --- |
| Rgs_energy | -7.82 | -7.79 | 0.4 | 2.34 | 2.41 | -0.32 | -0.30 | 2.85 | 2.91 | 0.032 | 0.42 |
| Acc_energy | -4.56 | -4.61 | 1.1 | 1.89 | 1.93 | -0.21 | -0.24 | 2.76 | 2.81 | 0.028 | 0.38 |
| Rgt_energy | -15.23 | -15.18 | 0.3 | 3.12 | 3.08 | -0.45 | -0.42 | 3.12 | 3.08 | 0.021 | 0.51 |
| Sm_6mer | 5.67 | 5.71 | 0.7 | 0.45 | 0.47 | -0.18 | -0.20 | 2.34 | 2.38 | 0.035 | 0.33 |
| Rgs_match | 2.34 | 2.31 | 1.3 | 0.56 | 0.58 | 0.12 | 0.15 | 1.98 | 2.02 | 0.041 | 0.29 |
| Sm_7mer_m8 | 0.42 | 0.43 | 2.4 | 0.24 | 0.25 | 0.34 | 0.31 | 1.45 | 1.48 | 0.045 | 0.31 |
| Sm_7mer_m1 | 0.38 | 0.39 | 2.6 | 0.23 | 0.24 | 0.41 | 0.38 | 1.38 | 1.42 | 0.052 | 0.27 |
| Sm_7mer_A1 | 0.36 | 0.35 | 2.8 | 0.22 | 0.23 | 0.56 | 0.52 | 1.42 | 1.45 | 0.048 | 0.35 |
| Consv_seed | 0.67 | 0.68 | 1.5 | 0.12 | 0.12 | -0.23 | -0.21 | 2.12 | 2.15 | 0.018 | 0.62 |
| 2mer1 | 0.22 | 0.21 | 4.5 | 0.18 | 0.19 | 1.23 | 1.19 | 3.45 | 3.51 | 0.062 | 0.24 |
| Consv_3cntxt | 0.58 | 0.59 | 1.7 | 0.15 | 0.15 | -0.34 | -0.32 | 2.34 | 2.31 | 0.023 | 0.48 |
| Rgs_mismatch | 1.23 | 1.25 | 1.6 | 0.89 | 0.92 | 0.67 | 0.64 | 2.89 | 2.94 | 0.038 | 0.41 |
| 2mer12 | 0.15 | 0.14 | 6.7 | 0.12 | 0.13 | 1.89 | 1.92 | 4.12 | 4.23 | 0.071 | 0.19 |
| Consv_5cntxt | 0.52 | 0.53 | 1.9 | 0.14 | 0.14 | -0.28 | -0.26 | 2.21 | 2.18 | 0.026 | 0.53 |
| Nt1 | 2.45 | 2.48 | 1.2 | 1.23 | 1.25 | -0.12 | -0.10 | 1.89 | 1.92 | 0.029 | 0.45 |
| 2mer7 | 0.08 | 0.08 | 0.0 | 0.07 | 0.08 | 2.34 | 2.28 | 5.67 | 5.78 | 0.058 | 0.28 |
| 2mer6 | 0.06 | 0.06 | 0.0 | 0.06 | 0.06 | 2.56 | 2.49 | 6.12 | 6.21 | 0.055 | 0.32 |
| Rgt_match | 3.21 | 3.19 | 0.6 | 1.45 | 1.48 | 0.08 | 0.1 | 2.15 | 2.18 | 0.025 | 0.44 |
| **Average** | - | - | **1.8** | - | - | - | - | - | - | **0.039** | **0.37** |

*Abbreviations: KL , Kullback-Leibler; KS, Kolmogorov-Smirnov.*

*Feature correlation preservation: To assess whether interdependencies among features are preserved, we computed the pairwise Pearson correlation matrices for original and synthetic negative samples. The average absolute difference in correlation coefficients across all 18 × 18 feature pairs was 0.042 ± 0.031 (range: 0.011–0.103). This low value indicates that biologically relevant feature correlations (e.g., between seed region matching and thermodynamic stability) are well maintained without introducing artificial relationships.*

*Interpretation: All KS tests yielded *p* > 0.05, indicating no statistically significant differences between original and synthetic distributions. The average KL divergence of 0.039 (close to zero) and average relative difference of 1.8% across all statistical moments further confirm that HD-MTD preserves the distributional characteristics of real biological data without introducing significant bias. The improved predictive performance achieved with augmented data (Table 3) can therefore be attributed to effective data supplementation rather than synthetic data bias.*
