# Supplementary material for: MicroRNA target gene prediction model based on input-feature dependency and sample data expansion technique
Source: PLoS Comput Biol. 2026 Jun 11;22(6):e1014402. doi: 10.1371/journal.pcbi.1014402 (PMC13258019; doi:10.1371/journal.pcbi.1014402)
Supplement: S5 Table — (DOCX) [file pcbi.1014402.s005.docx]

****Table S5. Statistical significance of performance comparisons between the proposed model and baseline models.****

| Comparison | F1 score difference | p-value (F1) | AUC difference | p-value (AUC) |
| --- | --- | --- | --- | --- |
| Proposed vs.GMM without dependency | 0.0722 | < 0.001 | 0.07 | < 0.001 |
| Proposed vs. SGM considering dependency | 0.1198 | < 0.001 | 0.13 | < 0.001 |
| Proposed vs.WD | 0.1698 | <0.001 | 0.17 | <0.001 |
| Proposed vs. miRBench-CNN | 0.0012 | 0.032 | 0.02 | 0.028 |
| Proposed vs. Hybrid AE-CNN | 0.0002 | 0.041 | 0.04 | 0.015 |

*Note: Raw p-values are reported. The Bonferroni correction for five comparisons (proposed model vs. each of five baselines) sets the significance threshold at α = 0.01. ***p* < 0.001, **p* < 0.01, *p* < 0.05. After correction, only comparisons with *p* < 0.01 are considered statistically significant.*
