# Supplementary material for: MicroRNA target gene prediction model based on input-feature dependency and sample data expansion technique
Source: PLoS Comput Biol. 2026 Jun 11;22(6):e1014402. doi: 10.1371/journal.pcbi.1014402 (PMC13258019; doi:10.1371/journal.pcbi.1014402)
Supplement: S1 File — S2 Fig. Recombinant plasmid map of pmirGLO-JAK2-WT. S3 Fig. Relative luciferase activity. S4 Fig. Dual-luciferase reporter assay results for miR-8485 inhibitor. S5 Fig. miR-8485 mimic and inhibitor sequences. S6 Fig. Dual-luciferase reporter assay results for miR-8485 mimics. S7 Fig. Binding site of hsa-miR-8485 on JAK2 3′UTR. S8 Fig. JAK2 reporter gene detection report. S1 Protocol. JAK2 reporter gene plasmid construction protocol. (ZIP) [file pcbi.1014402.s006.zip › R2Dual luciferase assay-JAK2- miR-8485/Plasmid/S1 Protocol. JAK2 reporter gene plasmid construction protocol.pdf]

JAK2 Report Gene Plasmid Construction Protocol

Genetic Information

Species Name： Mice

Gene Name： JAK2

Template Source: Genomic Retrieval

Construction Information

Cloning Vector： pmirGLO

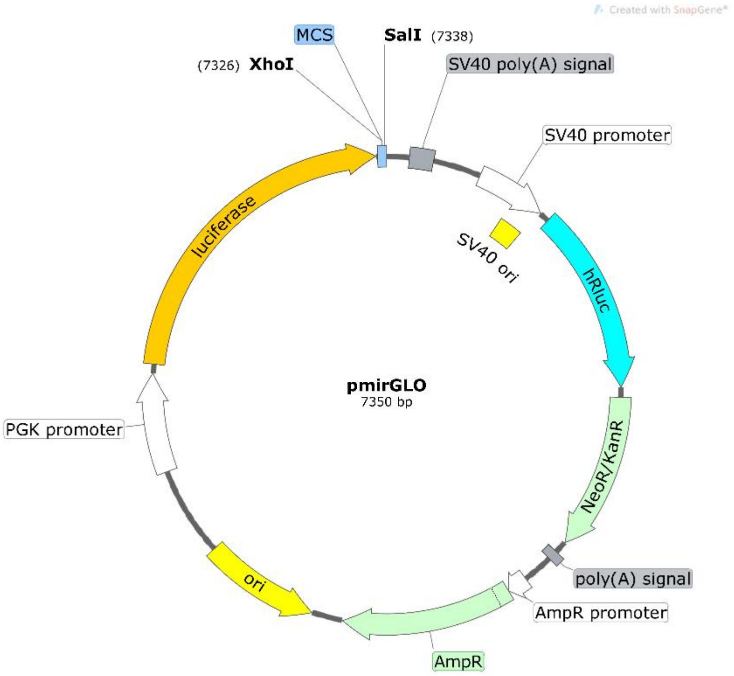

Cloning sites： Xho I / Sal I

Target sequence

JAK2 -WT (Length 306 bp, red mark indicates miRNA binding site.)

caagaatgccagtagaaaattcataacgtgtatctttaagaaaaatgagcatacatct

taaatcttttcaattaagtataaggggttgttcgttgttgtcatttgttatagtgctactcc

actttagacaccatagctaaaataaaatatggtgggttttgtgtgtgtgtgtgtgtgtg

tgtgtgtgtgtgtgtgtgtgttatttatacaaaacttaaaatacttgctgttttgattaaaa

agaaaatagtttcttactttatttttactggtatgttctactttttgaaagttgtactgaag

acttctgattttgggttgaagggaaggaaaaggaagaaatg

JAK2-Mut (length 345 bp)

caagaatgccagtagaaaattcataacgtgtatctttaagaaaaatgagcatacatct

taaatcttttcaattaagtataaggggttgttcgttgttgtcatttgttatagtgctactcc

actttagacaccatagctaaaataaaatatggtgggtttttatttatacaaaacttaaa

atacttgctgttttgattaaaaagaaaatagtttcttactttatttttactggtatgttctact

ttttgaaagttgtactgaagacttctgattttgggttgaagggaaggaaaaggaag

aaatg

Recombinant Plasmid Construction

Primary Components： pGK pro-Luc2-MCS-SV40 pro-Rluc

Recombinant Plasmid Map:

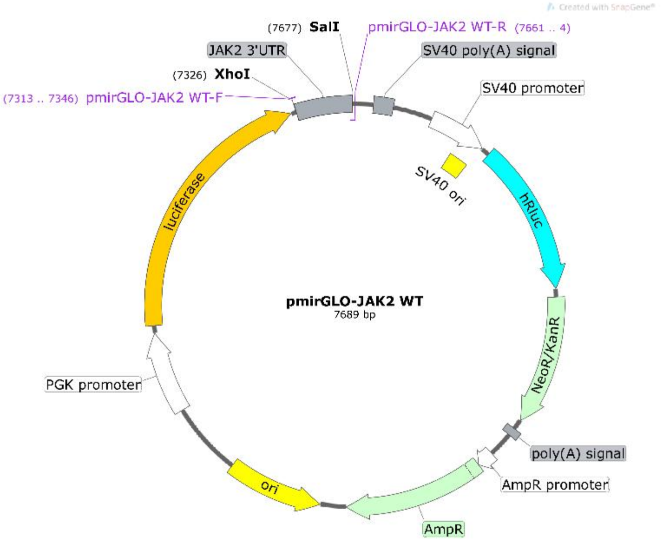

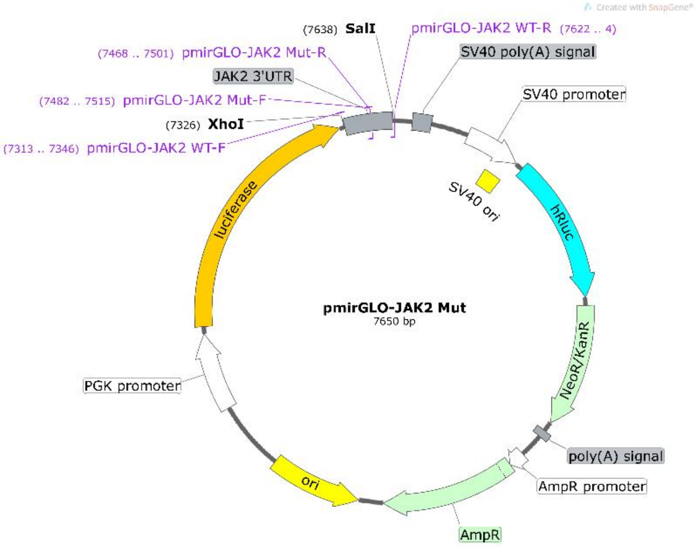

Primer Information：

Primer Name	Primer Sequence（5’-3’）
pmirGLO-JAK2 -WT-F	GAGCTCGCTAGCCTCGAGTGATAGAAGAGGTCCG
pmirGLO-JAK2 -WT-R	CATGCCTGCAGGTCGACTTATTACAGCAAACAC
pmirGLO-JAK2 -Mut-R	CATGCCTGCAGGTCGACTTATTATCGTGAACACAGCAACACAAG
