# Supplementary material for: MicroRNA target gene prediction model based on input-feature dependency and sample data expansion technique
Source: PLoS Comput Biol. 2026 Jun 11;22(6):e1014402. doi: 10.1371/journal.pcbi.1014402 (PMC13258019; doi:10.1371/journal.pcbi.1014402)
Supplement: S1 File — S2 Fig. Recombinant plasmid map of pmirGLO-JAK2-WT. S3 Fig. Relative luciferase activity. S4 Fig. Dual-luciferase reporter assay results for miR-8485 inhibitor. S5 Fig. miR-8485 mimic and inhibitor sequences. S6 Fig. Dual-luciferase reporter assay results for miR-8485 mimics. S7 Fig. Binding site of hsa-miR-8485 on JAK2 3′UTR. S8 Fig. JAK2 reporter gene detection report. S1 Protocol. JAK2 reporter gene plasmid construction protocol. (ZIP) [file pcbi.1014402.s006.zip › R2Dual luciferase assay-JAK2- miR-8485/Rep_gene_data/S4 Fig. Dual-luciferase reporter assay results for miR-8485 inhibitor.pdf]

3' UTR Reporter Gene (Luciferase) Assay

I.Experimental Objective:

microRNA regulates target gene expression primarily by binding to the 3' untranslated region (3' UTR) of target mRNA, leading to target mRNA degradation or inhibition of protein synthesis.

In our 3' UTR reporter gene system, the 3' UTR region of the target gene is constructed downstream of the luciferase reporter gene. By comparing changes in reporter gene expression following microRNA overexpression (monitored through alterations in luciferase activity), we can quantitatively reflect the inhibitory effect of microRNA on the target gene.

II.Experimental Information:

**Target Cells:**	HL-1
**Transfection System:**	24-well plate, 500 μl/well
**Assay Time:**	48 hours post-plasmid transfection
**Total Plasmid Co-Transfection Amount:**	3' UTR Luciferase plasmid 0.4 μg, miRNA inhibitor 100 pM (final concentration)

III. Explanation of Plasmid Names in the Detection System:

**miRNA-NC:**	microRNA control
**miRNA:**	hsa-miR-8485 inhibitor
**3' UTR WT:**	Target gene 3' UTR plasmid (JAK2-WT)
**3' UTR-Mutant:**	Target gene 3' UTR mutant plasmid (JAK2-Mut MRE mutation)

IV. Experimental Groups:

Group Number	Corresponding Group Name
**Experimental Group 1**	JAK2 WT+miRNA-NC
**Experimental Group 2**	JAK2 WT+hsa-miR-8485 inhibitor
**Experimental Group 3**	JAK2 Mutant+miRNA-NC
**Experimental Group 4**	JAK2 Mutant+hsa-miR-8485 inhibitor

V. Raw Data from Luciferase Assay

Assay Parameter	Experimental Group	Duplicate 1 Data	Duplicate 2 Data	Duplicate 3 Data
**Firefly luminescence**	Experimental Group 1	108550	123114	112727
	Experimental Group 2	432841	401110	370944
	Experimental Group 3	142592	159282	123626
	Experimental Group 4	141839	116787	136420

Assay Parameter	Experimental Group	Duplicate 1 Data	Duplicate 2 Data	Duplicate 3 Data
**Renilla luminescence**	Experimental Group 1	1153	1348	1130
	Experimental Group 2	1059	1277	1219
	Experimental Group 3	1300	1398	1079
	Experimental Group 4	1166	1292	1450

VI. Data Processing:

Firefly /Renilla luminescence: The ratio of Firefly Luciferase to Renilla Luciferase values within the same sample well, representing relative luciferase expression.

Firefly /Renilla luminescence fold change: In two groups transfected with the same luciferase plasmid, normalize the relative luciferase expression of the miRNA-NC group to 1.

The relative luciferase expression of the target miRNA group compared to the miRNA-NC group.

1. Firefly /Renilla luminescence

	Experimental Group	Duplicate 1 Data	Duplicate 2 Data	Duplicate 3 Data
**Firefly /Renilla luminescence**	Experimental Group 1	94.15	91.33	99.76
	Experimental Group 2	408.73	314.10	304.30
	Experimental Group 3	109.69	113.94	114.57
	Experimental Group 4	121.65	90.39	94.08

Mean and Deviation:

	Experimental Group	AVERAGE	STDEV.
**Firefly /Renilla luminescence**	Experimental Group 1	95.08	4.29
	Experimental Group 2	342.38	57.67
	Experimental Group 3	112.73	2.66
	Experimental Group 4	102.04	17.08

2. Firefly /Renilla luminescence fold change

	Experimental Group	Duplicate 1 Data	Duplicate 2 Data	Duplicate 3 Data
**Firefly /Renilla luminescence fold change**	Experimental Group 1	0.99	0.96	1.05
	Experimental Group 2	4.30	3.30	3.20
	Experimental Group 3	0.97	1.01	1.02
	Experimental Group 4	1.08	0.80	0.83

Mean and Deviation:

	Experimental Group	AVERAGE	STDEV.
**Firefly /Renilla luminescence fold change**	Experimental Group 1	1.00	0.05
	Experimental Group 2	3.60	0.61
	Experimental Group 3	1.00	0.02
	Experimental Group 4	0.91	0.15
