# Supplementary material for: MicroRNA target gene prediction model based on input-feature dependency and sample data expansion technique
Source: PLoS Comput Biol. 2026 Jun 11;22(6):e1014402. doi: 10.1371/journal.pcbi.1014402 (PMC13258019; doi:10.1371/journal.pcbi.1014402)
Supplement: S1 File — S2 Fig. Recombinant plasmid map of pmirGLO-JAK2-WT. S3 Fig. Relative luciferase activity. S4 Fig. Dual-luciferase reporter assay results for miR-8485 inhibitor. S5 Fig. miR-8485 mimic and inhibitor sequences. S6 Fig. Dual-luciferase reporter assay results for miR-8485 mimics. S7 Fig. Binding site of hsa-miR-8485 on JAK2 3′UTR. S8 Fig. JAK2 reporter gene detection report. S1 Protocol. JAK2 reporter gene plasmid construction protocol. (ZIP) [file pcbi.1014402.s006.zip › R2Dual luciferase assay-JAK2- miR-8485/Rep_gene_data/S5 Fig. miR-8485 mimic and inhibitor sequences.pdf]

mimic NC sense:
5′ - UUGUACUACACAAAAGUACUG-3′
mimic NC ansense:
5′ - GUACUUUUGUGUAGUACAAUU-3′
hsa-miR-8485 mimic sense:
5′ - CACACACACACACACACGUAU-3′
hsa-miR-8485 mimic Antisense:
5′ - ACGUGUGUGUGUGUGUGUGCA-3′

inhibitor NC:
5′ -CAGUACUUUUGUGUAGUACAA-3′
hsa-miR-8485 inhibitor:
5′ - AUACGUGUGUGUGUGUGUG-3′
