# Supplementary material for: MicroRNA target gene prediction model based on input-feature dependency and sample data expansion technique
Source: PLoS Comput Biol. 2026 Jun 11;22(6):e1014402. doi: 10.1371/journal.pcbi.1014402 (PMC13258019; doi:10.1371/journal.pcbi.1014402)
Supplement: S1 File — S2 Fig. Recombinant plasmid map of pmirGLO-JAK2-WT. S3 Fig. Relative luciferase activity. S4 Fig. Dual-luciferase reporter assay results for miR-8485 inhibitor. S5 Fig. miR-8485 mimic and inhibitor sequences. S6 Fig. Dual-luciferase reporter assay results for miR-8485 mimics. S7 Fig. Binding site of hsa-miR-8485 on JAK2 3′UTR. S8 Fig. JAK2 reporter gene detection report. S1 Protocol. JAK2 reporter gene plasmid construction protocol. (ZIP) [file pcbi.1014402.s006.zip › R2Dual luciferase assay-JAK2- miR-8485/Test_report/S8 Fig. JAK2 reporter gene detection report. .pdf]

JAK2 Reporter Gene Detection Report

Experimental Reagents and Instruments:

	Name	Supplier	Part Number
Laboratory Reagents	Dual Luciferase Reporter Assay Kit	Vazyme	DL101-01
	Exfect 2000 Transfection Reagent	Vazyme	T202-01
	Medium-Quantity Kit for Endotoxin-Free Plasmid DNA	Tiangen	DP118
Laboratory Instruments	GloMax 20/20 Luminometer	Promega	

Grouping Settings:

(Three biological replicates per group)

	JAK2-3' UTR WT (0.4 ug)	JAK2-3' UTR Mut (0.4 ug)	Mimics NC (50 pmol)	hsa-miR-8485 mimics (50 pmol)	Inhibitor NC (50 pmol)	hsa-miR-8485 inhibitor (50 pmol)
1	+		+			
2	+			+		
3	+				+	
4	+					+
5		+	+			
6		+		+		
7		+			+	
8		+				+

Plasmid Extraction：

Perform endotoxin-free extraction of the constructed reporter gene recombinant plasmids pmirGLO-JAK2-WT/pmirGLO-JAK2-Mut using the endotoxin-free plasmid mini/midipreparation kit provided by Tiangen Biotech Co., Ltd. (Extraction steps are detailed in the attached operating manual).

Cell Transfection：

Plate thawed, viable HL-1 cells uniformly into 24-well plates. Initiate transfection when cell confluence reaches approximately 80%. Employ Exfect 2000 Transfection Reagent from Novogene for transfection according to pre-designed groupings (refer to the appendix for specific procedures).

Plasmid Extraction：

Perform endotoxin-free extraction of the constructed reporter gene recombinant plasmids pmirGLO-JAK2-WT/pmirGLO-JAK2-Mut using the endotoxin-free plasmid mini/midipreparation kit provided by Tiangen Biotech Co., Ltd. (Extraction steps are detailed in the attached operating manual).

Luminescence Detection：

The luminescence values of firefly and Renilla were read separately using Promega's GloMax 20/20 Microplate Reader after sample digestion.

Data Processing：

After calculating the firefly luciferase/Renilla luciferase ratio, data analysis was performed using GraphPad Prism software (see attached Excel spreadsheet for analyzed data). The results are as follows：

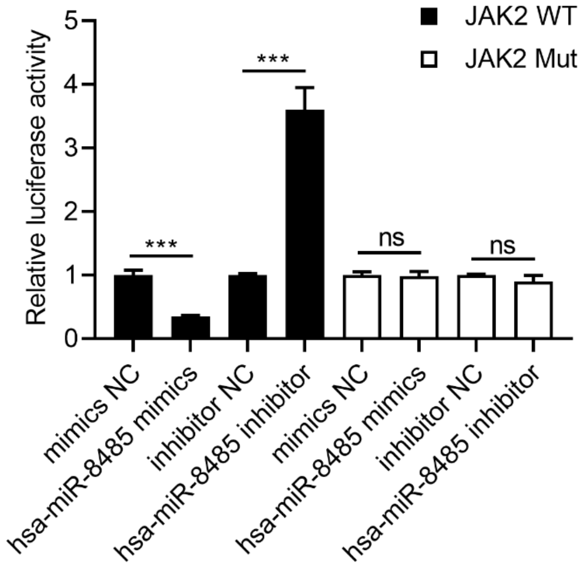

JAK2 WT
JAK2 Mut
Relative luciferase activity
0
1
2
3
4
5
***
***
ns
ns
mimics NC
hsa-miR-8485 mimics
inhibitor NC
hsa-miR-8485 inhibitor
mimics NC
hsa-miR-8485 mimics
inhibitor NC
hsa-miR-8485 inhibitor
