# Supplementary material for: MicroRNA target gene prediction model based on input-feature dependency and sample data expansion technique
Source: PLoS Comput Biol. 2026 Jun 11;22(6):e1014402. doi: 10.1371/journal.pcbi.1014402 (PMC13258019; doi:10.1371/journal.pcbi.1014402)
Supplement: S1 File — S2 Fig. Recombinant plasmid map of pmirGLO-JAK2-WT. S3 Fig. Relative luciferase activity. S4 Fig. Dual-luciferase reporter assay results for miR-8485 inhibitor. S5 Fig. miR-8485 mimic and inhibitor sequences. S6 Fig. Dual-luciferase reporter assay results for miR-8485 mimics. S7 Fig. Binding site of hsa-miR-8485 on JAK2 3′UTR. S8 Fig. JAK2 reporter gene detection report. S1 Protocol. JAK2 reporter gene plasmid construction protocol. (ZIP) [file S7 Fig. Binding site of hsa-miR-8485 on JAK2 3′UTR.pdf › R2Dual luciferase assay-JAK2- miR-8485/Site_mapping/S7 Fig. Binding site of hsa-miR-8485 on JAK2 3′UTR.pdf]

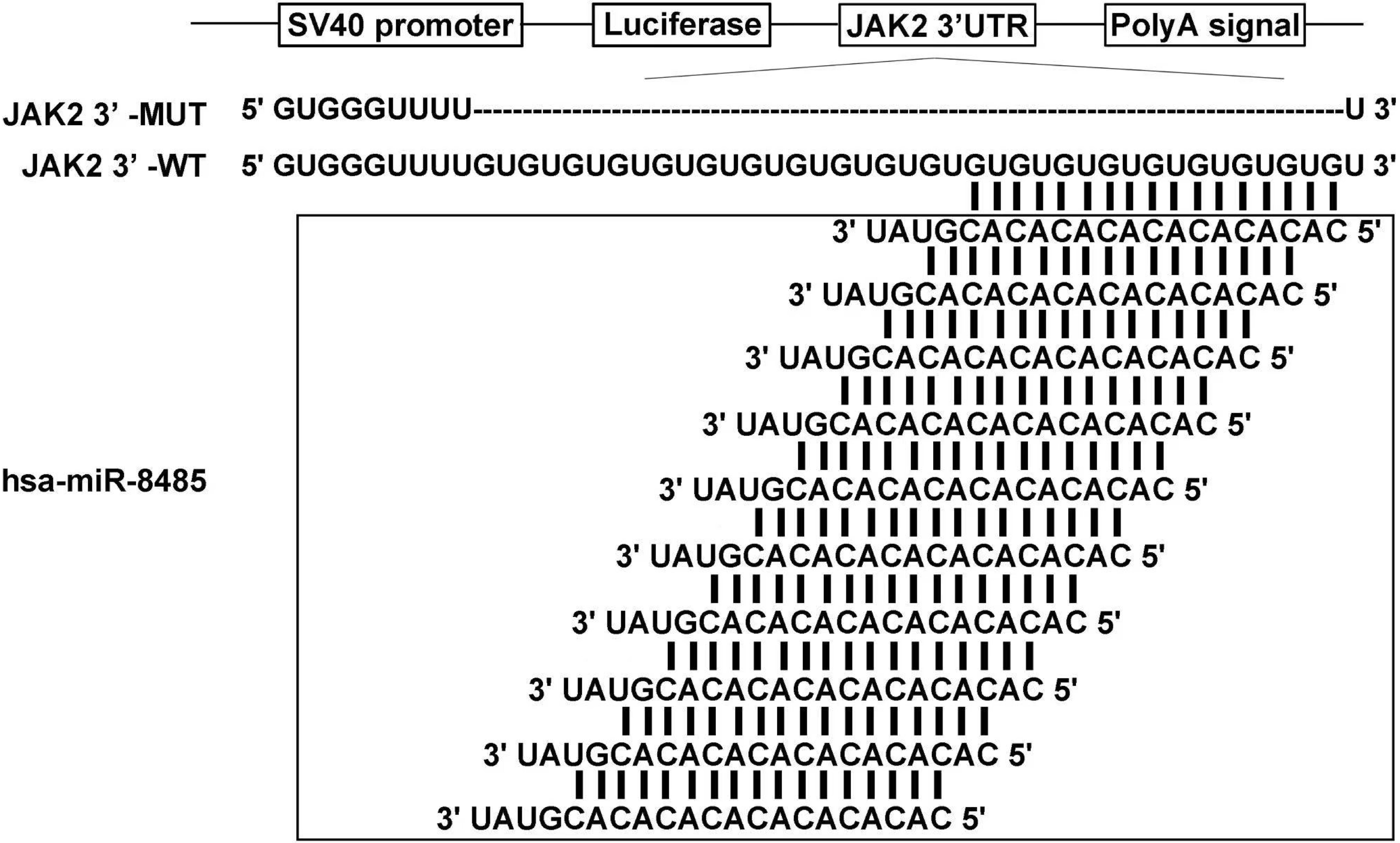

SV40 promoter
Luciferase
JAK2 3'UTR
PolyA signal
JAK2 3' -MUT
5' GUGGGUUUU--U 3'
JAK2 3' -WT
5' GUGGGUUUUGUGUGUGUGUGUGUGUGUGUGUGUGUGUGUGUGU 3'
hsa-miR-8485
3' UAUGCACACACACACACACAC 5'
