# Supplementary material for: MicroRNA target gene prediction model based on input-feature dependency and sample data expansion technique
Source: PLoS Comput Biol. 2026 Jun 11;22(6):e1014402. doi: 10.1371/journal.pcbi.1014402 (PMC13258019; doi:10.1371/journal.pcbi.1014402)
Supplement: Attachment — Submitted filename: Response_to_Reviewers (1)R1.docx [file pcbi.1014402.s008.docx]

**Responses to Reviewers’ Comments**

Dr. Feilim Mac Gabhann and Dr. Virginia Pitzer

Editors-in-Chief

*PLOS Computational Biology*

Dear Editor:

We wish to resubmit the manuscript titled “MicroRNA target gene prediction model based on input-feature dependency and sample data expansion technique.” The manuscript ID is PCOMPBIOL-D-26-00186.

We thank you and the reviewers for your thoughtful suggestions and insights. The manuscript has benefited from these insightful suggestions. We look forward to working with you and the reviewers to ensure that this manuscript meets the publication standards of *PLOS Computational Biology.*

The manuscript has been rechecked and the necessary changes have been made in accordance with the reviewers’ suggestions. The responses to all comments have been prepared and included below.

Thank you for your consideration. We look forward to hearing from you.

Best regards,

Shimin Dong

Department of Emergency, The Third Hospital of Hebei Medical University

Shijiazhuang, Hebei 050051, China

Email: [dongsm@hebmu.edu.cn](mailto:dongsm@hebmu.edu.cn)

**Reviewer#1, Concern # 1: The authors must immediately clarify a critical ambiguity in the methodological framework that fundamentally undermines the reproducibility of this work. Specifically, the transition from the 90 initially extracted features to the final 18-feature set, selected via the G-means criterion, lacks a transparent reporting of the feature selection process itself. While equation 19 defines a feature effectiveness measure γᵢ, the manuscript fails to disclose the algorithm or procedure used to solve the convex quadratic programming problem that yields the optimal weight vector ω*. The reader is left to assume a standard support vector machine implementation, but this must be explicitly stated, including the specific solver, kernel function (if any, despite the linear decision function implied), and the method for tuning the penalty parameter C. Without this information, the derived γᵢ values and the subsequent ranking of features cannot be verified or replicated by the community, rendering the core of the model's interpretability claims unsubstantiated. The authors must provide a complete algorithmic description, including all hyperparameters, in a revised methods section.**

**Author response:** We thank the reviewer for this important comment regarding the clarity and reproducibility of our feature selection procedure.

To address this concern, we have provided a complete and explicit algorithmic description of the method used to obtain the optimal weight vector. Specifically, we have clarified that the convex quadratic programming problem (Eq. 21) was solved using a linear Support Vector Machine (SVM). We now explicitly describe the implementation details, including the solver, kernel function, and hyperparameter optimization strategy. The revised text has been added to the Methods section

These additions ensure that the feature selection process, including all relevant hyperparameters and optimization procedures, is now fully transparent and reproducible.

The corresponding revisions are as follows:

$\left\{ \begin{aligned} \text{min}_{\text{ω}\text{，}\text{b}}\frac{\text{1}}{\text{2}}\left\| \text{ω}_{\text{i}} \right\|^{\text{2}}\text{+}\text{C}\sum_{\text{i}\text{=1}}^{\text{n}} \text{ε}_{\text{i}} \\ \text{s.t. }\text{y}_{\text{i}}\left（ {\text{ω}_{\text{i}}}^{\text{T}}\text{x}_{\text{i}}\text{+}\text{b} \right）\text{≥1-}\text{ε}_{\text{i}}\text{，}\text{ε}_{\text{i}}\text{≥0} \end{aligned} \right.$ (21)

where $\left\| \text{ω}_{\text{I}} \right\|$ represents the norm of the weight vector, $\text{ρ}\text{≥0}$ denotes the margin variable, $\text{C}$ is the penalty parameter, and $\text{ε}_{\text{i}}\text{≥0}$ are the slack variables.

To obtain the optimal weight vector *ω*, we solved the convex quadratic programming problem defined in Eq. (21) using a linear Support Vector Machine (SVM). The model was implemented using the LinearSVC class from the scikit-learn library in Python, with the liblinear solver selected. A linear kernel was used, consistent with the linear decision function shown in Eq. (20). The penalty parameter *C* was optimized via 5-fold cross-validation on the training set. We performed a grid search over *C* $\text{∈}$ {0.01, 0.1, 1, 10, 100} and selected the value that maximized the average G-means score across the validation folds. The optimal *C* value was found to be 1.

**Reviewer#1, Concern # 2: The justification for using the Pareto principle to reduce the feature set from 90 to 18 is presented as a fait accompli and is scientifically unsound in this context. The authors state that "80% of the effects are determined by the most important 20% of the characteristics," but this empirical rule from economics and quality control is not a biological law and cannot be used to arbitrarily truncate a feature set. The decision to fix k=18 based on the observation that G-means plateaus after this point is a valid empirical approach, but referencing the Pareto principle adds no scientific value and introduces a logical fallacy. The authors must remove this spurious justification and instead frame the feature selection purely as an optimization based on the G-means criterion, which is already a sufficient and valid method. The current wording detracts from the otherwise sound methodological approach.**

**Author response:** We thank the reviewer for this insightful comment regarding the justification of our feature selection strategy. We agree that the Pareto principle is not an appropriate scientific basis for feature selection in this context and that its inclusion may introduce unnecessary conceptual ambiguity.

To address this concern, we have removed all references to the Pareto principle from the manuscript. The feature selection process is now described solely as an empirical optimization procedure based on the G-means criterion, which provides a direct and data-driven method for determining the optimal number of features. Specifically, we clarify that the number of features 𝑘 was selected by evaluating model performance across different feature subset sizes and identifying the point at which the G-means score reaches a plateau. This approach ensures that the selected feature set balances predictive performance and model complexity without relying on external heuristic assumptions.

The revised description has been incorporated into the Methods section.

The corresponding revisions are as follows:

The most important *k* features directly associated with the biological mechanism of miRNA targeting were selected from all 90 characteristics as input features for the prediction model, whereas the remaining characteristics were treated as redundant information. This approach effectively reduced computational costs.

The feature effectiveness measure $\text{γ}_{\text{I}}$ is used to calculate the importance of each feature:

$\text{γ}_{\text{i}}\text{=}\left( \frac{\text{ω}^{\text{*}}\text{(}\text{i}\text{)}}{\left\| \text{ω}^{\text{*}} \right\|} \right)^{\text{2}}$ (22)

where $\text{ω}^{\text{*}}\text{(}\text{i}\text{)}$ is the *i*-th element of $\text{ω}_{\text{i}}$.

The 90 features were sorted based on their $\text{γ}_{\text{I}}$ values in descending order, and the top *k* features with the highest $\text{γ}_{\text{I}}$ values were selected.

The value of *k* was determined using the G-means ($\text{∈}\text{[0,1]}$), which is the geometric mean of TP and TN; a value closer to 1 indicates better model performance. When the number of features was 18, G-means approached its peak value, indicating optimal performance. Further increasing the number of features did not significantly improve the G-means but substantially increased computational cost. Therefore, *k* = 18 was selected as the optimal feature set size. The values and weights of the input features selected for this study are presented in Table 1.

**Reviewer#1, Concern # 3: The derivation and presentation of the joint probability density function in equation 7 are mathematically incomplete and potentially incorrect, requiring an immediate revision. The equation presents the joint density f(T₁,...,T_d) as a product of marginal densities f_d(T_d) and a product of bivariate copula densities. However, the notation f_d(T_d) is poorly defined; it should clearly indicate that it is the product of all marginal densities, i.e., ∏ f_i(T_i). More critically, the indices in the second product term are ambiguous. The notation ∏_{k∈E_i} P_{y(k),z(k)|M(k)} is technically correct for a vine copula, but the authors have failed to define the arguments of these copula density functions in the equation itself. A copula density takes as inputs the conditional distribution functions, e.g., c(F(T_y|T_M), F(T_z|T_M)). While these are defined in equation 8 for a specific case, they must be included as arguments in the main joint density formula to be mathematically rigorous. The authors must rewrite equation 7 to include the proper arguments for the copula densities, ensuring the equation is a complete and accurate representation of an R-vine density.**

**Author response:** We thank the reviewer for this important and technically detailed comment regarding the formulation of the joint probability density function. We agree that the previous presentation lacked sufficient clarity and mathematical completeness, particularly in the definition of the marginal density term and the explicit specification of the copula arguments.

To address this concern, we have revised the corresponding equations to provide a complete and rigorous representation of the R-vine copula density. Specifically, we have clarified that the joint density is expressed as the product of all marginal densities, rather than using the previously ambiguous notation.

In addition, we now explicitly include the arguments of the bivariate copula density functions in the formulation. The revised equations define each copula density in terms of the corresponding conditional distribution functions, thereby ensuring mathematical correctness and consistency with standard R-vine copula formulations.

These corrections have been incorporated into the revised manuscript under Methods section, where all terms and notations are now clearly defined.

The corresponding revisions are as follows:

$\text{f}_{\text{c}}\text{(}\text{T}_{\text{1}}\text{,}\text{T}_{\text{2}}\text{,}\text{⋯}\text{,}\text{T}_{\text{d}}\text{)=[}\prod_{\text{i}\text{=1}}^{\text{d}} \text{f}_{\text{c}\text{,}\text{i}}\text{(}\text{T}_{\text{i}}\text{)]∙}\text{[}\prod_{\text{t}\text{=1}}^{\text{d}\text{-1}} \prod_{\text{e}\text{∈}\text{E}_{\text{t}}} \text{P}_{\text{y}\text{(}\text{e}\text{), }\text{z}\text{(}\text{e}\text{) | }\text{M}\text{(}\text{e}\text{)}}\text{(}\text{F}_{\text{c}}\text{(}\text{T}_{\text{y}\text{(}\text{e}\text{)}}\text{|}\text{T}_{\text{M}\text{(}\text{e}\text{)}}\text{)}\text{,}\text{F}_{\text{c}}\text{(}\text{T}_{\text{z}\text{(}\text{e}\text{)}}\text{|}\text{T}_{\text{M}\text{(}\text{e}\text{)}}\text{))}\text{]}$	(9)

$\text{F}_{\text{c}}\text{(}\text{T}_{\text{y(e)}}\text{|}\text{T}_{\text{M(e)}}\text{)=}\frac{\text{∂}\text{P}_{\text{y}\text{(}\text{e}\text{), }\text{z}\text{(}\text{e}\text{) | M(e)}}\text{(}\text{F}_{\text{c}}\text{(}\text{T}_{\text{y(e)}}\text{|}\text{T}_{\text{D(e)}}\text{),}\text{F}_{\text{c}}\text{(}\text{T}_{\text{z(e)}}\text{|}\text{T}_{\text{D(e)}}\text{))}}{\text{∂}\text{F}_{\text{c}}\text{(}\text{T}_{\text{z(e)}}\text{|}\text{T}_{\text{D(e)}}\text{)}}$ (10)

Where$\text{f}_{\text{c}\text{,}\text{i}}\text{(}\text{T}_{\text{i}}\text{)}$is the marginal density of the *i*-th feature for class$\text{ }\text{c}$, and$\text{F}_{\text{c}}\text{(}\text{∙}\text{|}\text{∙}\text{)}$ are the class-specific conditional distribution functions. $\text{P}_{\text{y}\text{€}\text{, }\text{z}\text{€}\text{ | }\text{M}\text{€}}$is the bivariate copula density for the pair ($\text{y}\text{€}\text{, }\text{z}\text{€}$) conditioned on the set $\text{M}\text{€}$.

**Reviewer#1, Concern # 4: The experimental validation, while commendable for including in-vitro and in-vivo work, is fundamentally confounded by a selection bias that must be addressed. The authors validated a single prediction—miR-8485 targeting JAK2. Selecting only one successful validation case constitutes "cherry-picking" and provides no information about the model's false positive rate or its performance across the diverse set of its predictions. The dual-luciferase, cellular, and animal experiments are powerful demonstrations of the biological relevance of one predicted interaction, but they do not constitute a validation of the model's overall predictive accuracy. To substantiate their claim of high accuracy, the authors must either provide a statistical summary of validation experiments on a randomly selected, representative cohort of predictions (e.g., 20-30 predicted interactions) or, at a minimum, report on the outcome of any negative or failed validations they may have performed. Presenting a single positive case is anecdotal evidence, not model validation.**

**Author response:** We sincerely thank the reviewer for this critical and insightful comment regarding the interpretation of our experimental validation. We fully agree that validating a single predicted interaction (miR-8485 targeting JAK2) does not constitute a statistical validation of the model's overall predictive performance and may introduce concerns regarding selection bias.

To address this concern, we have revised the manuscript to clearly reposition the role of the experimental validation. Specifically, we now present this experiment as a proof-of-concept case study demonstrating that predictions generated by our model can be biologically validated, rather than as evidence of overall model accuracy.

Accordingly, we have made the following revisions:

Reframing of experimental validation: In the Abstract, Author summary, and Conclusion, In the Abstract, Author Summary, and Conclusion, the experimental results are now explicitly described as a proof-of-concept demonstration of biological plausibility, highlighting the model’s utility for hypothesis generation rather than its predictive accuracy.

Clarification of experimental purpose: We have revised the relevant sections to clearly state that the dual-luciferase, cellular, and animal experiments were conducted to validate the biological relevance of a single predicted interaction, not to provide a statistical evaluation of model performance.

Explicit acknowledgment of limitations: We have added a dedicated discussion in the final paragraph of the Discussion section acknowledging that validation on a larger, randomly selected cohort of predictions is required to assess the model’s false positive rate and generalizability. This is now presented as an important direction for future work.

These revisions ensure that the scope and limitations of the experimental validation are clearly communicated and that no overstated claims regarding model accuracy are made.

The corresponding revisions are as follows:

## Abstract

Predicting microRNA target genes is essential for understanding their biological functions. This study developed a miRNA target gene prediction model based on input-feature dependency. Features were treated as multiple random variables, with marginal densities estimated via Gaussian mixture models and dependencies captured by regular vine copula to derive joint probability density functions. We constructed class-conditional joint densities for positive and negative samples separately using GMM and R-vine copula, then combined these with prior probabilities via Bayes' rule to obtain posterior probabilities of positive interactions, using a standard 0.5 probability threshold for deterministic prediction. To address insufficient data and class imbalance, hybrid distribution megatrend diffusion was used to generate virtual samples for data augmentation. Computational validation showed high predictive performance even with only 30% of the original dataset. As proof of concept, we experimentally validated one predicted interaction (miR-8485 targeting *JAK2*) using dual-luciferase, cellular, and animal experiments, confirming biological relevance. These findings provide a valuable tool for understanding miRNA functions and disease mechanisms.

**Author summary**

In this study, we developed a new computational model to more accurately predict which genes are regulated by microRNAs—small RNA molecules that play key roles in health and disease. Predicting these targets is difficult because biological data are often limited, imbalanced, and contain complex relationships between features. Our model addresses these challenges by combining two innovations: a probabilistic prediction framework that accounts for dependencies between input features, and a data expansion method that generates realistic synthetic samples to balance the dataset. Computational experiments show that our model performs well even when trained on only 30% of the original data and outperforms existing methods in predictive accuracy. Through laboratory experiments, we validated one prediction—that miR-8485 targets the *JAK2* gene—serving as a proof-of-concept demonstration of the model's biological relevance. Our findings provide researchers with a promising tool for uncovering microRNA functions, which can help advance our understanding of diseases and support the development of new therapies.

**Experimental validation: a proof-of-concept case study**

To demonstrate the biological relevance of predictions generated by the model, we selected a top-ranked prediction—miR-8485 targeting *JAK2*—for experimental validation. It is important to note that this single-case validation is intended as a proof-of-concept to illustrate that the model can generate biologically plausible hypotheses, rather than as a statistical validation of the model's overall predictive accuracy. The following dual-luciferase, cellular, and animal experiments were performed to evaluate the biological validity of this specific predicted interaction.

## Discussion

This study has several limitations. First, experimental validation was performed on only one predicted interaction (miR-8485 targeting *JAK2*); while the dual-luciferase, cellular, and animal experiments confirmed the biological relevance of this specific prediction, a single positive case does not provide a statistical estimate of the model's false positive rate or its generalizability, and thus these results should be interpreted as a proof-of-concept demonstration of the model's utility for hypothesis generation rather than comprehensive validation of its predictive accuracy. Second, the training dataset (TarBase v8.0) is relatively small and imbalanced; although we employed HD-MTD to mitigate this issue, the synthetic samples may not fully capture the complexity of biological data.

Future work should address these limitations through multiple complementary directions. First, large-scale experimental validation on a randomly selected cohort of predictions (e.g., 20-30 interactions) is needed to rigorously assess the model's false positive rate. Second, incorporating additional training data from high-throughput technologies and integrating more diverse biological features—such as spatial structure data or miRNA–mRNA interaction kinetics—could further enhance prediction accuracy [30]. Third, exploring alternative synthetic sample generation strategies that better preserve biologically meaningful distributions would improve the handling of imbalanced data.

**Conclusion**

We have established a robust miRNA target prediction framework that effectively integrates probabilistic modeling, feature dependency, and sample expansion. The proposed model demonstrates promising performance on benchmark datasets, and experimental validation of one predicted interaction (miR-8485 targeting *JAK2*) confirmed the biological relevance of the model's predictions, illustrating its utility for hypothesis generation. This framework provides a valuable tool for guiding experimental validation and functional studies on miRNAs, contributing to the growing arsenal of computational approaches for precision medicine research.

**Reviewer#1, Concern # 5:The description of the Hybrid Distribution Mega-Trend Diffusion (HD-MTD) technique is critically under-specified in the manuscript, preventing any meaningful evaluation or adoption of the method. Step 2 involves clustering the original data using K-means based on eight statistical attributes. The authors must specify the value of M (the number of clusters) and, crucially, justify how this number was determined. Was it based on an elbow method, a silhouette score, or was it arbitrarily chosen? Step 3 is even more problematic. The authors state they construct the PDF of each data cluster and then use a "skill score" to select the optimal distribution type from a set of candidates (e.g., normal, Weibull). The skill score formula presented in equation 11 is not a standard goodness-of-fit measure and is poorly explained. The term P_i represents the original data, but the comparison with γ_l, the PDF of a distribution type, is nonsensical—a probability density value cannot be directly compared to a data point in this manner. The skill score calculation appears mathematically incoherent. The authors must provide a clear, step-by-step mathematical definition of their skill score, explaining how it quantifies the fit between a continuous probability density function and a discrete set of data points.**

**Author response:** We thank the reviewer for this important and detailed comment regarding the specification and mathematical validity of the HD-MTD method. We agree that the previous description lacked sufficient clarity, particularly in the determination of the number of clusters and the definition of the goodness-of-fit measure used for distribution selection.

To address these concerns, we have made the following revisions:

Specification and justification of the number of clusters (Step 2):

We now explicitly state that the number of clusters 𝑀 was determined using the elbow method based on the within-cluster sum of squares (WCSS). Specifically, K-means clustering was performed for a range of candidate values of 𝑀, and the optimal number was selected at the point where the reduction in WCSS began to diminish. This procedure was conducted within each training fold to prevent data leakage. The revised description has been added to the Methods section .

Replacement of the skill score with a standard goodness-of-fit measure (Step 3):

We agree with the reviewer that the previously defined “skill score” was insufficiently justified and potentially mathematically inconsistent. Therefore, we have removed this formulation entirely and replaced it with the log-likelihood as a standard and well-established goodness-of-fit criterion.

Clarification of distribution fitting procedure:

For each cluster, candidate probability distributions (e.g., normal, Weibull, gamma, lognormal) are fitted using maximum likelihood estimation. The log-likelihood of the observed data under each candidate distribution is then computed, and the distribution with the highest log-likelihood is selected as the optimal model for that cluster. This provides a principled and statistically sound method for distribution selection.

These revisions ensure that the HD-MTD procedure is now fully specified, mathematically coherent, and reproducible.

The corresponding revisions are as follows:

**Sample data expansion technique**

Step 2: Clustering of Original Data

The optimal number of clusters M was determined using the elbow method based on the within-cluster sum of squares (WCSS). Specifically, we performed K-means clustering for M ranging from 2 to 10 and plotted the WCSS against the number of clusters. The optimal M was selected as the point where the decrease in WCSS began to diminish, forming an 'elbow' in the curve. The optimal number of clusters was determined to be M=3.

Considering the eight input attributes of the training set as inputs to the K-means algorithm, all the original data were clustered into M clusters.

Step 3: Assignment of Corresponding Distribution Types to Each Cluster

For each cluster obtained in Step 2, we aim to select the optimal distribution that best characterizes its data. To achieve this, we the log-likelihood as the goodness-of-fit measure.

For a given cluster, we consider a set of candidate distribution families, including but not limited to the normal, Weibull, gamma, and lognormal distributions. For each candidate distribution, we first estimate its parameters $\text{θ}$ via maximum likelihood estimation based on the data points in that cluster

The log-likelihood (LL) of the cluster data under the candidate distribution is then computed as

$\text{LL}\text{=}\sum_{\text{j}\text{=1}}^{\text{N}_{\text{cluster}}} \text{log}\text{ }\text{f}\text{(}\text{x}_{\text{j}}\text{|}\text{θ}\text{)}$	(14)

where $\text{f(}\text{x}_{\text{j}}\text{|θ)}$ is the PDF of the candidate distribution with the estimated parameters $\text{θ}$. The LL quantifies how likely it is to observe the given cluster data under a specific distribution; a higher value indicates a better fit. For each cluster, we select the candidate distribution that yields the maximum log-likelihood as its optimal distribution type. This process is performed independently for every cluster. Crucially, to prevent data leakage, this entire selection process—including parameter estimation and log-likelihood calculation—is performed within each cross-validation fold using only the training data. The validation folds remain untouched during this step.

**Reviewer#1, Concern #6: The manuscript contains a fundamental error in the interpretation of the marginal probability density functions used within the copula framework. The authors state they treat the "18 features of the target gene... as multiple random variables" and estimate their marginal PDFs using a GMM. However, in a standard copula model for prediction, the variables are not the raw features themselves. The correct procedure is to model the conditional distribution of the target variable (e.g., the binary label of positive/negative interaction) given the features, or to model the joint distribution of the features conditioned on the class label. By modeling the raw features directly as T₁...T_d and plugging them into a vine copula, the authors are implicitly building a model of the feature distribution, not a predictive model for the target gene. This is a category error. The authors must completely re-frame their probabilistic model. They need to clarify whether they are building two separate copula models—one for the feature distribution of positive samples and one for negative samples—and then using Bayes' rule to derive a prediction, or if they have employed a different conditional copula approach. As it stands, the methodological description conflates feature distribution modeling with predictive modeling.**

**Author response:** We thank the reviewer for this important and insightful comment regarding the interpretation of the probabilistic modeling framework. We agree that the original description may have lacked clarity and could give the impression that we were modeling only the marginal distribution of features without a predictive objective.

To clarify, our approach follows a class-conditional generative modeling framework combined with Bayesian classification, which is a standard and well-established approach for supervised prediction. Specifically, we have revised the manuscript to clearly state the following:

1. Class-conditional modeling: We construct two separate probabilistic models: one for the positive class (miRNA target genes) and one for the negative class (non-target genes). For each class, the joint distribution of the feature vector is modeled using Gaussian mixture models (GMMs) for marginal densities and an R-vine copula to capture dependencies among features. This yields class-conditional joint densities.

2. Prediction via Bayes’ rule: Prediction is then performed using Bayes’ rule. For a new instance with feature vector TTT, the posterior probability of belonging to the positive class is computed as:

$$\text{P}\text{(}\text{pos}\text{|}\text{T}\text{)=}\frac{\text{f}\text{(}\text{T}\text{|}\text{pos}\text{)∙}\text{P}\text{(}\text{pos}\text{)}}{\text{f}\text{(}\text{T}\text{|}\text{pos}\text{)∙}\text{P}\text{(}\text{pos}\text{)+}\text{f}\text{(}\text{T}\text{|}\text{neg}\text{)∙}\text{P}\text{(}\text{neg}\text{)}}$$

Where, $\text{f}\text{(}\text{T}\text{|}\text{pos}\text{)}$ and $\text{f}\text{(}\text{T}\text{|neg)}\text{ }$are the class-conditional joint densities estimated by the GMM and R-V-copula models, and$\text{ }\text{P}\text{(}\text{pos}\text{)}$ and $\text{P}\text{(}\text{neg}\text{)}$ are the prior probabilities estimated from the training data. A threshold of 0.5 is then applied to obtain the final deterministic prediction.

Clarification of model interpretation: We have revised the manuscript to explicitly state that the R-vine copula models the class-conditional joint distribution of features, rather than an unconditional feature distribution. This distinction ensures that the model is correctly interpreted as a predictive model rather than a purely descriptive one.

These clarifications have been incorporated into the R-V-copula section, and corresponding revisions have also been made in the Abstract and Discussion . We believe these changes resolve the ambiguity and accurately reflect the intended modeling framework.

The corresponding revisions are as follows:

## Abstract

Predicting microRNA target genes is essential for understanding their biological functions. This study developed a miRNA target gene prediction model based on input-feature dependency. Features were treated as multiple random variables, with marginal densities estimated via Gaussian mixture models and dependencies captured by regular vine copula to derive joint probability density functions. We constructed class-conditional joint densities for positive and negative samples separately using GMM and R-vine copula, then combined these with prior probabilities via Bayes' rule to obtain posterior probabilities of positive interactions, using a 0.5 threshold for deterministic prediction. To address insufficient data and class imbalance, hybrid distribution mega-trend diffusion generated virtual samples. Computational validation showed high predictive performance even with only 30% of the original dataset. As proof of concept, we experimentally validated one predicted interaction (miR-8485 targeting *JAK2*) using dual-luciferase, cellular, and animal experiments, confirming biological relevance. These findings provide a valuable tool for understanding miRNA functions and disease mechanisms.

**R-V-copula**

The copula function is the most commonly used tool to establish the dependency between marginal PDFs [24]. To construct a predictive model, we adopted a class-conditional generative approach. Specifically, we built two separate R-V-copula models: one for the positive samples (miRNA target genes) and one for the negative samples (non-target genes). For each class $\text{c}\text{ϵ\{}\text{pos}\text{,}\text{neg}\text{\}}$, we used a GMM to estimate the marginal PDFs of the 18 features, and a R-V-copula to capture the dependencies among these features. This yielded two class-conditional joint PDFs, $\text{f}\text{(}\text{T}\text{|}\text{pos}\text{)}$, and$\text{f}\text{(}\text{T}\text{|}\text{neg}\text{)}$, where $T=\left( T_{1},T_{2},\cdots T_{18} \right)$ is the feature vector of a given miRNA-target gene pair.

(2) Modeling the R-V-copula function

To avoid information leakage, all vine structure selection and copula family determination were performed within each cross-validation fold using only the training data.

First, the edge set ({*y, z|M*}) of each tree was determined. Considering the accuracy and computational complexity of the model, an edge with a high dependency on the first few trees was selected. Dependency was measured using the Kendall rank correlation coefficient [25]. We used the maximum spanning tree algorithm to select the edge with the largest sum of absolute empirical Kendall rank correlation coefficient.

Second, we considered the choice of the binary copula corresponding to each edge. Additionally, we calculated the AIC for all alternative binary copula families [26]. The binary copula function with the smallest AIC was considered to have the best goodness of fit and was therefore selected as the first choice for each edge. The AIC was obtained using the following formula:

$\text{AIC}\text{=2}\text{d}\text{-2}\text{I}\text{n}\text{(L̂)}$ (7)

where d was the parameter number and L̂ is the maximized likelihood of the sample set. The higher the value of d, the more complex the model.

For Copula parameter estimation, for each selected bivariate copula family, the copula parameters were estimated by maximum likelihood estimation (MLE). Given a set of observations${{\text{\{(F}}_{\text{c}}\text{(}\text{T}_{\text{y}\text{€}}\text{|}\text{T}_{\text{M}\text{€}}\text{), }\text{F}_{\text{c}}\text{(}\text{T}_{\text{z}\text{€}}\text{|}\text{T}_{\text{M}\text{€}}\text{)}\text{)}}_{\text{i=1}}^{\text{n}}$for the two variables, the log-likelihood function is:

$\text{L}\text{(}\text{θ}_{\text{y}\text{(}\text{e}\text{), }\text{z}\text{(}\text{e}\text{) | }\text{M}\text{(}\text{e}\text{)}}\text{)=}\sum_{\text{i}\text{=1}}^{\text{n}} \text{log}\text{P}_{\text{y}\text{(}\text{e}\text{), }\text{z}\text{(}\text{e}\text{) | }\text{M}\text{(}\text{e}\text{)}}\text{(}\text{F}_{\text{c}}\text{(}{\text{T}_{\text{y}\text{(}\text{e}\text{)}}}^{\text{(i)}}\text{|}{\text{T}_{\text{M}\text{(}\text{e}\text{)}}}^{\text{(}\text{i}\text{)}}\text{,}\text{F}_{\text{c}}\text{(}{\text{T}_{\text{z}\text{(}\text{e}\text{)}}}^{\text{(i)}}\text{|}{\text{T}_{\text{M}\text{(}\text{e}\text{)}}}^{\text{(}\text{i}\text{)}}\text{)}\text{;}\text{θ}_{\text{y}\text{(}\text{e}\text{), }\text{z}\text{(}\text{e}\text{) | }\text{M}\text{(}\text{e}\text{)}}$ (8)

Where $\text{θ}_{\text{y}\text{€}\text{, }\text{z}\text{€}\text{ | }\text{M}\text{€}}$ is the copula parameters for edge e. The MLE was obtained by numerical optimization using the sequential quadratic programming (SQP) algorithm implemented in the rvinecopulib package.

For samples belonging to a given class $\text{c}\text{ϵ\{}\text{pos}\text{,}\text{neg}\text{\}}$, the class-conditional joint probability density function of the 18 features can be expressed as:

$\text{f}_{\text{c}}\text{(}\text{T}_{\text{1}}\text{, }\text{T}_{\text{2}}\text{,}\text{⋯}\text{,}\text{T}_{\text{d}}\text{)=[}\prod_{\text{i}\text{=1}}^{\text{d}} \text{f}_{\text{c}\text{,}\text{i}}\text{(}\text{T}_{\text{i}}\text{)]∙}\text{[}\prod_{\text{t}\text{=1}}^{\text{d}\text{-1}} \prod_{\text{e}\text{∈}\text{E}_{\text{t}}} \text{P}_{\text{y}\text{(}\text{e}\text{), }\text{z}\text{(}\text{e}\text{) | }\text{M}\text{(}\text{e}\text{)}}\text{(}\text{F}_{\text{c}}\text{(}\text{T}_{\text{y}\text{(}\text{e}\text{)}}\text{|}\text{T}_{\text{M}\text{(}\text{e}\text{)}}\text{),}\text{F}_{\text{c}}\text{(}\text{T}_{\text{z}\text{(}\text{e}\text{)}}\text{|}\text{T}_{\text{M}\text{(}\text{e}\text{)}}\text{))]}$	(9)

$\text{F}_{\text{c}}\text{(}\text{T}_{\text{y(e)}}\text{|}\text{T}_{\text{M(e)}}\text{)=}\frac{\text{∂}\text{P}_{\text{y}\text{(}\text{e}\text{), }\text{z}\text{(}\text{e}\text{) | M(e)}}\text{(}\text{F}_{\text{c}}\text{(}\text{T}_{\text{y(e)}}\text{|}\text{T}_{\text{D(e)}}\text{),}\text{F}_{\text{c}}\text{(}\text{T}_{\text{z(e)}}\text{|}\text{T}_{\text{D(e)}}\text{))}}{\text{∂}\text{F}_{\text{c}}\text{(}\text{T}_{\text{z(e)}}\text{|}\text{T}_{\text{D(e)}}\text{)}}$ (10)

Where$\text{f}_{\text{c}\text{,}\text{i}}\text{(}\text{T}_{\text{i}}\text{)}$is the marginal density of the *i*-th feature for class$\text{c}$, and$\text{F}_{\text{c}}\text{(}\text{∙}\text{|}\text{∙}\text{)}$ are the class-specific conditional distribution functions. $\text{P}_{\text{y}\text{€}\text{, }\text{z}\text{€}\text{ | }\text{M}\text{€}}$is the bivariate copula density for the pair ($\text{y}\text{€}\text{, }\text{z}\text{€}$) conditioned on the set $\text{M}\text{€}$.

Finally, prediction was performed using Bayes' rule. For a new instance with a feature vector  $T=\left( T_{1},T_{2},\cdots T_{18} \right)$, the posterior probability of being a positive target was computed using:

$\text{P}\text{(}\text{pos}\text{|}\text{T}\text{)=}\frac{\text{f}\text{(}\text{T}\text{|}\text{pos}\text{)}\text{∙}\text{P}\text{(}\text{pos}\text{)}}{\text{f}\text{(}\text{T}\text{|}\text{pos}\text{)}\text{∙}\text{P}\text{(}\text{pos}\text{)+}\text{f}\text{(}\text{T}\text{|}\text{neg}\text{)}\text{∙}\text{P}\text{(}\text{neg}\text{)}}$ (11)

Where, $f\text{(}\text{T}\text{|}\text{pos}\text{)}$ and $f\text{(}\text{T}\text{|neg)}$are the class-conditional joint densities estimated by the GMM and R-V-copula models, and$\text{P}\text{(}\text{pos}\text{)}$ and $\text{P}\text{(}\text{neg}\text{)}$ are the prior probabilities estimated from the training data. Based on the class distribution in our dataset (831 positive and 306 negative samples), the priors were set to $\text{P}\text{(}\text{pos}\text{)}$=831/1137≈0.731, and $\text{P}\text{(}\text{neg}\text{)}$=306/1137≈0.269.

The final deterministic prediction was obtained by thresholding the posterior probability at 0.5: if $\text{P}\text{(}\text{pos}\text{|}\text{T}\text{)}$>0.5, the instance was classified as a positive target; otherwise, it was classified as negative.

## Discussion

In this study, we developed a class-conditional generative model for miRNA target gene prediction that differs fundamentally from conventional discriminative approaches and employs an HD-based sample expansion technique. While many existing methods (e.g., DNNs, XGBoost) can produce probabilistic outputs, they typically do so by learning decision boundaries rather than explicitly modeling the joint distribution of features. Our approach, in contrast, explicitly learns the class-conditional joint distribution through a combination of GMM and R-V-copula. This generative framework offers unique advantages: (1) it explicitly models dependencies among features rather than assuming independence or learning boundaries without capturing underlying distributions; (2) it provides principled uncertainty quantification through the posterior probability derived from Bayes' rule; and (3) HD–MTD was used to construct virtual samples to effectively enhance prediction accuracy and to solve the problem of insufficient total sample data.

**Reviewer#1, Concern # 7: The authors' critique of existing methods as "deterministic" and the positioning of their work as a "probabilistic prediction" model is overstated and misrepresents the field. Many machine learning models, including the DNN and XGBoost models they cite, provide probabilistic outputs. For instance, a neural network with a softmax output layer produces a probability distribution over classes. XGBoost can output calibrated probabilities. The novelty, therefore, is not in producing a probability per se, but in the specific architecture of using a vine copula to model feature dependencies for this purpose. The authors must moderate their claims and reframe their contribution accurately. They should state that they are introducing a novel probabilistic architecture based on generative modeling through copulas, rather than implying that all existing models only output a hard binary classification without an associated confidence or probability score. This distinction is crucial for an honest positioning of their work within the existing literature.**

**Author response:** We thank the reviewer for this important comment regarding the positioning of our work relative to existing methods. We agree that many modern machine learning approaches, including deep neural networks and gradient boosting models, are capable of producing probabilistic outputs (e.g., via softmax layers or calibrated probabilities), and that our original wording may have overstated this distinction.

To address this concern, we have revised the manuscript to moderate our claims and more accurately reflect the contribution of our work. Specifically, we no longer contrast our method with existing approaches based on the ability to produce probabilistic outputs. Instead, we now emphasize that the novelty of our approach lies in its generative modeling framework, which differs fundamentally from commonly used discriminative models. In particular, our method explicitly models the class-conditional joint distribution of features using Gaussian mixture models (for marginal distributions) and a regular vine copula (for dependency structure). This allows us to capture complex inter-feature dependencies and perform prediction through Bayes’ rule.

We have revised the relevant descriptions in the Introduction and Discussion sections to clearly reflect this distinction and ensure accurate positioning within the existing literature.

The corresponding revisions are as follows:

## Introduction

The first problem is the low accuracy of the predictions. Target gene prediction can be classified into deterministic and probabilistic predictions according to the different prediction results. The result obtained from deterministic prediction is a specific result with a more intuitive form (e.g., whether the target gene corresponds to miRNA) [13]. The result of a probabilistic prediction is a probability distribution that can provide an uncertainty analysis for the prediction [14]. While many existing machine learning models for target gene prediction can produce probabilistic outputs (e.g., through softmax layers or calibrated probability estimates), these approaches typically operate within a discriminative framework that does not explicitly model feature dependencies. For instance, the degree of seed region base sequence matching and miRNA:mRNA dimer thermodynamic stability are two commonly used prediction characteristics. In general, if the base-matching degree is high, the thermodynamics are more stable; thus, the two are positively dependent [15]. Therefore, to increase prediction accuracy, one can establish a probabilistic prediction model that considers the dependency of all input features combined with deterministic prediction to obtain complete prediction results and accurately identify target genes regulated by miRNAs.

Therefore, in this study, we constructed a miRNA target gene prediction model based on input-feature dependency. First, 18 features of the target gene were considered as input for multiple random variables, and a class-conditional generative modeling strategy was employed. Gaussian mixture models and regular vine copulas were used to estimate the joint density of features separately for positive and negative samples. These class-conditional densities were then integrated with prior probabilities through Bayes' theorem to compute the posterior probability of a true interaction. The posterior probabilities we obtain arise from explicitly modeling the class-conditional joint distributions of features. This generative framework enables us to capture complex feature dependencies that are often overlooked in conventional approaches. A decision threshold of 0.5 was applied to this posterior probability to obtain binary classifications. Finally, HD–MTD was used to construct virtual samples to effectively enhance prediction accuracy of the parametric and non-parametric models and to solve the problem of insufficient total sample data and uneven proportions of negative and positive samples.

## Discussion

In this study, we developed a class-conditional generative model for miRNA target gene prediction that differs fundamentally from conventional discriminative approaches and employs an HD-based sample expansion technique. While many existing methods (e.g., DNNs, XGBoost) can produce probabilistic outputs, they typically do so by learning decision boundaries rather than explicitly modeling the joint distribution of features. Our approach, in contrast, explicitly learns the class-conditional joint distribution through a combination of GMM and R-V-copula. This generative framework offers unique advantages: (1) it explicitly models dependencies among features rather than assuming independence or learning boundaries without capturing underlying distributions; (2) it provides principled uncertainty quantification through the posterior probability derived from Bayes' rule; and (3) HD–MTD was used to construct virtual samples to effectively enhance prediction accuracy and to solve the problem of insufficient total sample data.

**Reviewer#1, Concern # 8: The handling of the imbalance between positive (831) and negative (306) samples is methodologically weak. The authors use HD-MTD to generate virtual negative samples to balance the dataset. However, they fail to specify the target ratio after expansion. Do they generate negatives until a 1:1 ratio is achieved? Furthermore, the validation of this approach in Table 3 is flawed. When evaluating on 30% of the data, they use a smaller subset of the original, imbalanced data, expand it, and then test on... what? The test set is not described. To properly evaluate the data expansion technique, the authors must perform cross-validation *after* data expansion in a way that prevents data leakage. The standard approach is to expand the training fold only and keep the test fold pristine with original, non-synthetic samples. The manuscript gives no indication that this was done. The authors must describe their exact cross-validation setup for the experiments in Table 3, explicitly stating how they prevented synthetic samples from contaminating the test sets. Without this, the reported performance metrics are likely optimistically biased and meaningless.**

**Author response:** We thank the reviewer for raising this this important and detailed comment regarding the handling of class imbalance and the experimental evaluation protocol. We agree that a clear description of the data expansion procedure and safeguards against data leakage is essential for ensuring the validity of the reported results. To address this concern, we have clarified and strengthened the methodological description as follows:

1. Target ratio after expansion:
   We confirm that virtual negative samples were generated until a 1:1 ratio between positive and negative samples was achieved in the training set. This has now been explicitly stated in the manuscript.
2. Train–test separation and prevention of data leakage:
   For all experimental conditions reported in Table 3 (100%, 50%, and 30% of the data), the original dataset was first split into training (70%) and test (30%) sets using stratified sampling. The test set was kept pristine and unchanged, containing only original (non-synthetic) samples, and was used exclusively for final evaluation.
3. Evaluation under reduced data settings (30% and 50%):
   For the 50% and 30% conditions, only the training set was subsampled to the desired size. HD-MTD was then applied exclusively to this reduced training set to generate virtual samples. The model was trained on the expanded training data and evaluated on the same untouched test set, ensuring consistent and unbiased evaluation across all conditions.
4. Cross-validation protocol during model development:
   During cross-validation, HD-MTD was applied only within each training fold, while the corresponding validation fold remained untouched. This strictly prevents synthetic samples from contaminating validation data and ensures that performance estimates reflect true generalization.

These clarifications have been incorporated into the revised manuscript. We believe that the revised description now clearly demonstrates that all evaluations were conducted under a leakage-free protocol and that the reported performance metrics are not affected by optimistic bias.

The corresponding revisions are as follows:

**Data set description**

The miRNA target gene prediction sample dataset was extracted from TarBase v8.0. The dataset comprised data of five higher mammals: *Homo sapiens*, *Rattus norvegicus*, *Mus musculus*, *Bos taurus*, and *Ovis aries*. After removing the polycyclic data from the miRNA and the mRNA dimer structure, 831 positive miRNA target genes and 306 negative miRNA target genes were identified. The dataset was randomly split into training (70%) and test (30%) sets, stratified by class label to preserve the original class distribution. The test set was held out entirely during model development and hyperparameter tuning and was used only for final performance evaluation.

**Performance evaluation under different data sizes**

To systematically evaluate the performance of our proposed model under limited data conditions and to assess the effectiveness of HD-MTD, we conducted experiments using 30%, 50%, and 100% of the original training data. For each data size condition, we applied three data expansion techniques (HD-MTD, DD-MTD, and traditional MTD) to generate virtual negative samples, balancing the positive-to-negative ratio to 1:1. The experimental protocol, detailed in the Methods section, involved 10 repeated runs with different random seeds to ensure statistical robustness. The mean F1 scores and 95% confidence intervals are presented in Table 3.

For the experiments evaluating data expansion techniques (Table 3), we adopted the following rigorous protocol to prevent data leakage:

(1) Data partitioning: For each experimental condition (using 100%, 50%, or 30% of the total data), the original dataset was first randomly split into training (70%) and test (30%) sets using stratified sampling to preserve the original class distribution.

(2) Data expansion: different types of MTD were applied exclusively to the training set to generate virtual negative samples. The target ratio after expansion was set to 1:1 (positive:negative), meaning we generated negative samples until the number of negative samples in the training set equaled the number of positive samples.

(3) Model training and evaluation: The model was trained on the expanded training set (containing both original and synthetic samples) and evaluated on the pristine test set (containing only original samples, with no synthetic data). This ensures that performance metrics reflect the model's generalization ability on real-world data.

(4) Robustness check: The entire procedure (splitting-expansion-training-evaluation) was repeated 10 times with different random seeds, and the average F1 scores are reported in Table 3.

**Reviewer#1, Concern # 9: The description of the 18 selected features in Table 1 is inconsistent, incomplete, and in one case, appears to contain a critical typo that obscures the feature's definition. Features like Rgs_energy (x3), Acc_energy (x4), and Rgt_energy (x5) are all described as "ΔG" values calculated by RNAfold, but no distinction is made between these three thermodynamic features. What is the biological or structural difference between them? The authors must provide a clear definition for each. More alarmingly, in the table, both Sm_7mer_m8 (x7) and Sm_7mer_A1 (x9) are given identical conditional definitions: "z2 - z8 complementary pairing with target g". This cannot be correct. Sm_7mer_A1 typically refers to a match opposite the first nucleotide of the miRNA, which is often an adenine. The definition is almost certainly wrong and must be corrected immediately, as this is a standard and important feature in miRNA targeting. The authors must review the entire table for accuracy and provide complete, unambiguous definitions for every feature.**

**Author response:** We thank the reviewer for this careful and insightful evaluation of Table 1. We agree that the previous version contained insufficiently defined features and an error in the description of certain sequence-based features, which could lead to ambiguity. To address these concerns, we have thoroughly revised Table 1 and the corresponding descriptions in the manuscript as follows:

1. Clarification of thermodynamic features: We have provided explicit definitions for each thermodynamic feature derived from RNAfold. Specifically, the distinctions between Rgs_energy, Acc_energy, and Rgt_energy are now clearly described in terms of the structural context and the biological interpretation of the corresponding ΔG values (e.g., binding energy, accessibility energy, and target site structural stability). This ensures that each feature is uniquely defined and biologically meaningful.
2. Correction of feature definition error (Sm_7mer_A1): We confirm that the reviewer correctly identified an error in the definition of Sm_7mer_A1. This has been corrected. The revised definition now reflects the canonical 7mer-A1 seed match, which involves complementarity at positions 2–7 of the miRNA and an adenine at position 1 of the target site. The incorrect duplication of the Sm_7mer_m8 definition has been removed.
3. Comprehensive review of all feature definitions: We have carefully reviewed all 18 features to ensure that each definition is complete, accurate, and unambiguous. The revised table now provides clear and distinct descriptions for all features, eliminating inconsistencies and improving interpretability.

These revisions have been incorporated into Table 1 and the corresponding text . We believe that the updated descriptions fully resolve the issues raised by the reviewer.

The corresponding revisions are as follows:

**Table 1.** **Values and weights of each input feature.**

| **Feature** | **Value (**$\text{x}_{\text{i}}$**)** | **Weight (**$\text{ω}_{\text{i}}$**)** |
| --- | --- | --- |
| Sm_6mer | $\text{x}_{\text{1}}\text{=}\left\{ \begin{aligned} \text{6, }\text{if}\text{ }\text{Sm}\text{\_6}\text{me} \\ \text{5, }\text{if}\text{ }\text{Sm}\text{\_5}\text{me} \end{aligned} \right.$ | +0.85 |
| Rgs_match | $\text{x}_{\text{2}}\text{=}\left\{ \begin{aligned} \text{1, }\text{if}\text{ }\text{G}\text{-}\text{U}\text{ } \\ \text{2, }\text{if}\text{ }\text{A}\text{-}\text{U}\text{ } \\ \text{3, }\text{if}\text{ }\text{G}\text{-}\text{C}\text{ } \end{aligned} \right.$ | +0.72 |
| Rgs_energy | $\text{x}_{\text{3}}\text{ }$= ${\text{∆}\text{G}}_{\text{seed}}\text{=}\text{MFE}\text{(}\text{miRNA}_{\text{pos}\text{2-8}}\text{:}\text{mRNA}_{\text{seed}\text{ }\text{region}}\text{)}$  Minimum free energy (MFE) of the seed region (positions 2–8) duplex, calculated using RNAfold. More negative values indicate greater thermodynamic stability of the seed pairing. | -0.62 |
| Acc_energy | $\text{x}_{\text{4}}$ =${\text{ ∆}\text{G}}_{\text{accessibility}}\text{=}\text{MFE}\text{(}\text{mRNA}_{\text{local}\text{ }\text{context}}$)  Energy required to make the target site accessible for miRNA binding, estimated as the MFE of the local mRNA secondary structure surrounding the target site, calculated using RNAfold | -0.58 |
| Rgt_energy | $\text{x}_{\text{5 }}$= ${\text{∆}\text{G}}_{\text{total}}\text{=}\text{MFE}\text{(}\text{miRNA}_{\text{full}\text{ }\text{lengt}\text{h}}\text{:}\text{mRNA}_{\text{full}\text{ }\text{target}\text{ }\text{site}}\text{)}$  Minimum free energy of the full-length miRNA–mRNA duplex (including both seed and non-seed regions), calculated using RNAfold | -0.41 |
| Rgt_match | $\text{x}_{\text{6}}\text{=}\text{j}$  $\text{j}$ is the number of nucleotide matches in the non-seed region | +0.35 |
| Sm_7mer_m8 | $\text{x}_{\text{7}}\text{=}\left\{ \begin{aligned} \text{1}\text{, perfect match at position}\text{s}\text{ 2-8} \\ \text{0}\text{, }\text{ot}\text{h}\text{erwise} \end{aligned} \right.$ | +0.42 |
| Sm_7mer_m1 | $\text{x}_{\text{8}}\text{=}\left\{ \begin{aligned} \text{1}\text{, }\text{z}_{\text{1}}\text{-}{\text{ }\text{z}}_{\text{7}}\text{ }\text{c}\text{omplementary}\text{ }\text{pairing}\text{ }\text{wit}\text{h }\text{target}\text{ }\text{gene} \\ \text{0}\text{, }\text{ot}\text{h}\text{erwise} \end{aligned} \right.$ | +0.38 |
| Sm_7mer_A1 | $\text{x}_{\text{9}}\text{=}\left\{ \begin{aligned} \text{1, perfect match at position}\text{s}\text{ 2-8 and '}\text{A}\text{' }\text{opposite}\text{ }\text{miRNA} \\ \text{ }\text{position}\text{ 1} \\ \text{0, }\text{ot}\text{h}\text{erwise} \end{aligned}\text{ } \right.$ | +0.36 |
| Consv_seed | $\text{x}_{\text{10}}\text{=}\text{τ}_{\text{1}}$  $\text{τ}_{\text{1}}\text{∈}\text{[0,1] }$is the PhyloP score | +0.38 |
| 2mer1 | $\text{x}_{\text{11}}\text{=}\left\{ \begin{aligned} \text{1}\text{, }\text{z}_{\text{1}}\text{-}\text{z}_{\text{2}}\text{ }\text{c}\text{omplementary}\text{ }\text{pairing}\text{ }\text{wit}\text{h }\text{target}\text{ }\text{gene} \\ \text{0}\text{, }\text{ot}\text{h}\text{erwise} \end{aligned} \right.$ | +0.22 |
| Consv_3cntxt | $\text{x}_{\text{12}}\text{=}\text{τ}_{\text{2}}$  $\text{τ}_{\text{2}}\text{∈}\text{[0,1] }$is the PhyloP score | +0.18 |
| Rgs_mismatch | $\text{x}_{\text{13}}\text{=}\text{N}$  $\text{N}$ is the number of seed region mismatches | -0.45 |
| 2mer12 | $\text{x}_{\text{14}}\text{=}\left\{ \begin{aligned} \text{1}\text{, }\text{z}_{\text{12}}\text{-}\text{z}_{\text{13}}\text{ }\text{c}\text{omplementary}\text{ }\text{pairing}\text{ }\text{wit}\text{h }\text{target}\text{ }\text{gene} \\ \text{0}\text{, }\text{ot}\text{h}\text{erwise} \end{aligned} \right.$ | +0.15 |
| Consv_5cntxt | $\text{x}_{\text{15}}\text{=}\text{τ}_{\text{3}}$  $\text{τ}_{\text{3}}\text{∈}\text{[0,1] }$is the PhyloP score | +0.12 |
| Nt1 | $\text{x}_{\text{16}}\text{=ϑ}$  The value of $\text{ϑ}$ represents the base type at the first position of the target gene: $\text{ϑ }$= 1 for A, $\text{ϑ}$ = 2 for U, $\text{ϑ }$= 3 for G, and $\text{ϑ }$= 4 for C. | +0.10 |
| 2mer7 | $\text{x}_{\text{17}}\text{=}\left\{ \begin{aligned} \text{1}\text{, }\text{z}_{\text{7}}\text{-}\text{z}_{\text{8}}\text{ }\text{c}\text{omplementary}\text{ }\text{pairing}\text{ }\text{wit}\text{h }\text{target}\text{ }\text{gene} \\ \text{0}\text{, }\text{ot}\text{h}\text{erwise} \end{aligned} \right.$ | +0.08 |
| 2mer6 | $\text{x}_{\text{18}}\text{=}\left\{ \begin{aligned} \text{1}\text{, }\text{z}_{\text{6}}\text{-}\text{z}_{\text{7}}\text{ }\text{c}\text{omplementary}\text{ }\text{pairing}\text{ }\text{wit}\text{h }\text{target}\text{ }\text{gene} \\ \text{0}\text{, }\text{ot}\text{h}\text{erwise} \end{aligned} \right.$ | +0.06 |

**Reviewer#1, Concern # 10: The "Performance test of GMM" section and the accompanying Figure 1 are entirely uninterpretable and must be removed or completely re-conceived. The authors state they randomly selected five miRNAs and show the prediction results of different models, claiming the GMM peak is "closer to the actual target gene." However, for a binary classification task (target or not), a probability density function over a continuous variable does not have a concept of an "actual target gene." What is being plotted on the x-axis? It appears the authors are plotting the probability density of the decision function value S (from equation 17) for the five miRNAs. This is meaningless. A single miRNA has a fixed feature vector, which yields a single, deterministic value of S. Plotting a density around it is nonsensical. This figure reveals a deep misunderstanding of the model's own output. The GMM and copula are used to build a joint probability model; the prediction for a single instance should be a conditional probability, not a full density. This section and its corresponding figure demonstrate a fundamental flaw in how the authors conceptualize their own probabilistic prediction and must be completely omitted from the manuscript.**

**Author response:** We thank the reviewer for this incisive and technically detailed comment regarding the interpretation of the model outputs and the presentation in the “Performance test of GMM” section. We agree that the original formulation and visualization in this section were conceptually inappropriate and could lead to misinterpretation of the model’s probabilistic predictions. Following the reviewer’s recommendation, we have completely removed the entire “Performance test of GMM” section and Figure 1 from the manuscript.

To ensure that the role and effectiveness of GMM within our framework are still rigorously demonstrated, we emphasize that model performance is now evaluated exclusively using standard and statistically valid classification metrics, as presented elsewhere in the manuscript:

1. Quantitative performance comparison (Table 2): We provide comprehensive comparisons with alternative models (e.g., GMM without dependency, SGM with dependency, and WD) using standard metrics, including F1 score, accuracy, precision, recall, specificity, and AUC. These results demonstrate that the GMM-based approach consistently outperforms single-distribution models.
2. ROC analysis (Figure 3): ROC curves and corresponding AUC values are presented to provide a robust assessment of classification performance across decision thresholds.
3. Robustness under varying data sizes (Table 3): We evaluate model performance under different training data proportions, showing that the combination of GMM and HD-MTD maintains strong predictive capability even with limited data.

These revisions ensure that the evaluation of our model is now based entirely on appropriate and widely accepted statistical measures, and that no potentially misleading interpretations of probabilistic outputs remain in the manuscript.

**Reviewer#2, Concern # 1: For example, several sentences contain awkward phrasing and inconsistent terminology (e.g., switching between “target gene” vs. “target-gene,” and occasionally unclear antecedents), which reduces readability and precision in a methods-heavy paper. These language issues appear throughout the manuscript draft and should be corrected for a PLOS Computational Biology Research Article standard.**

**Author response:** We thank the reviewer for these valuable observations regarding the language quality and terminology consistency of our manuscript. We agree that precise and consistent language is essential, particularly for a methods-heavy paper such as ours.

To address these concerns, we have conducted a thorough revision of the entire manuscript. Specifically:

Standardized of terminology: We have carefully reviewed and unified key terms throughout the manuscript. In particular, we now consistently use "target gene" (as a noun, without hyphen) and "target-gene prediction" (as a compound adjective, with hyphen), in accordance with standard English usage.

Improvement of sentence clarity: Sentences that were overly long or awkwardly phrased sentences have been revised for clarity, readability and precision, while preserving the original meaning.

Comprehensive language review: The manuscript has been carefully proofread to correct typographical errors and inconsistencies and ensure that the overall language meets the standards expected for publication in *PLOS Computational Biology*.

We believe these revisions have significantly enhanced the clarity and readability of the manuscript. We are grateful to the reviewer for helping us strengthen the presentation of our work.

**Reviewer#2, Concern # 2: In Eq. (2), the normalizing constant is written as (1/\sqrt{(2\pi)^m\sigma_m^2}), which is dimensionally unclear for a (seemingly) 1-D Gaussian and mixes “(m)” (component index) with dimensionality; additionally, the text alternates between “k-th” and “m-th” sub-model when defining (\mu_m,\sigma_m^2,\gamma_m), which can confuse the meaning of parameters and prevents a clean step-by-step derivation from Eqs. (1)–(2).**

**Author response:** We thank the reviewer for identifying the dimensional inconsistency and indexing ambiguity in our original GMM formulation. We agree that the previous notation could lead to confusion regarding both the dimensionality of the Gaussian distribution and the indexing of mixture components.

To address these issues, we have revised Equations (1) and (2) to ensure mathematical correctness and clarity. Specifically:

1. Correction of the normalizing constant: The expression for the Gaussian density has been corrected to reflect the appropriate form for the specified dimensionality, eliminating the ambiguity caused by the previous notation.
2. Consistent indexing of mixture components: We now use a single, consistent index to denote mixture components throughout the formulation. The parameters are defined accordingly, ensuring consistency across all equations.
3. Clarification of notation and derivation: The surrounding text has been revised to clearly distinguish between component indices and data dimensionality, enabling a coherent and step-by-step interpretation of the GMM formulation.

These revisions have been incorporated into the manuscript, and we believe they resolve the inconsistencies identified by the reviewer.

The corresponding revisions are as follows:

The GMM PDF can be represented as follows [23]:

$\text{P}\text{(}\text{x}\text{|Θ)=}\sum_{\text{k}\text{=1}}^{\text{M}} \text{γ}_{\text{k}}\text{Ν}\text{(}\text{x}\text{|}\text{ϑ}_{\text{k}}\text{)}$ (1)

$N\left( x | \vartheta_{k} \right)$ = $\frac{1}{\left( 2\pi\right)^{\frac{d}{2}}\left| {\mathcal{\mathcal{L}}}_{k} \right|^{\frac{1}{2}}}\exp\left[ -\frac{1}{2}\left( x-\mu_{k} \right)^{T}{{\mathcal{\mathcal{L}}}_{k}}^{-1}\left( x-\mu_{k} \right) \right]$	(2)

The dataset is denoted as *D* =$\text{\{}\text{x}_{\text{1}}\text{,}\text{x}_{\text{2}}\text{,}\text{⋯}\text{,}\text{x}_{\text{n}}\text{\}}$, where *M* is the number of components, and *d* is the dimensionality. $\text{Μ}_{\text{k}}$, ${\mathcal{\mathcal{L}}}_{k}$, and $\text{γ}_{\text{k}}$ denoted the mean, covariance matrix, and mixing coefficient of the k-th component, respectively. Accordingly, $\text{ϑ}_{\text{k}}\text{ }$= ($\text{μ}_{\text{k}}$, ${\mathcal{\mathcal{L}}}_{k}$). The number of components for the GMM was selected from {1, 2, 3, 4, 5} based on the average log-likelihood in cross-validation.

**Reviewer#2, Concern # 3: In the vine structure definition, Eq. (5) is written in a way that is not mathematically coherent (“({y(k), z(k) = y(k), z(k)|M(k)})”), and Eq. (7) presents nested products with unclear index ranges and undefined symbols (e.g., the meaning of (\prod f_d(T_d)) and the product limits “(i=d-1)” / “(i=i)” are not readable as written), making it hard to verify correctness or reproduce the joint density construction.**

**Author response:** We thank the reviewer for these detailed comments regarding the clarity and correctness of our vine copula formulation. We agree that the original presentation of Eqs. (5) and (7) was not sufficiently clear and could hinder reproducibility.

To address these issues, we have carefully revised the equations and their accompanying descriptions to ensure mathematical coherence and clarity. Specifically:

1. Correction of vine structure notation (Eq. 5 → Eq. 6):
   The definition of the edge set and conditional relationships has been reformulated using standard R-vine notation, with all symbols clearly defined and consistently used.
2. Clarification of joint density formulation (Eq. 9 → Eq. 10):
   The joint probability density function has been rewritten to explicitly present:
   - the product of marginal densities, and
   - the product of pair-copula densities with clearly defined index ranges and arguments.
3. Improved notation and readability:
   All indices, product limits, and symbols have been standardized and clearly explained in the text, ensuring that the construction of the R-vine density can be followed step-by-step.

These revisions have been incorporated into the manuscript, and we believe they resolve the ambiguity and improve reproducibility.

The corresponding revisions are as follows:

*M(e)* = S($\text{e}_{\text{1}}$)∩S($\text{e}_{\text{2}}$) (5)

*e* = {*y*(*e*), *z*(*e*)*|M(e)*} (6)

where $\text{S}$($\text{∙}$) = $\text{\{}\text{y}\text{(∙), }\text{z}\text{(∙)|}\text{M}\text{(∙)}\text{\}}$ is a universal set of edges. For each edge $\text{e}$, under the given qualification, the binary copula density functions are $\text{P}_{\text{y}\left( \text{e} \right)\text{, }\text{z}\text{€}\text{ | }\text{M}\text{€}}$.

For samples belonging to a given class $\text{c}\text{ϵ\{}\text{pos}\text{,}\text{neg}\text{\}}$, the class-conditional joint probability density function of the 18 features can be expressed as:

$\text{f}_{\text{c}}\text{(}\text{T}_{\text{1}}\text{, }\text{T}_{\text{2}}\text{,}\text{⋯}\text{,}\text{T}_{\text{d}}\text{)=[}\prod_{\text{i}\text{=1}}^{\text{d}} \text{f}_{\text{c}\text{,}\text{i}}\text{(}\text{T}_{\text{i}}\text{)]∙}\text{[}\prod_{\text{t}\text{=1}}^{\text{d}\text{-1}} \prod_{\text{e}\text{∈}\text{E}_{\text{t}}} \text{P}_{\text{y}\text{(}\text{e}\text{), }\text{z}\text{(}\text{e}\text{) | }\text{M}\text{(}\text{e}\text{)}}\text{(}\text{F}_{\text{c}}\text{(}\text{T}_{\text{y}\text{(}\text{e}\text{)}}\text{|}\text{T}_{\text{M}\text{(}\text{e}\text{)}}\text{),}\text{F}_{\text{c}}\text{(}\text{T}_{\text{z}\text{(}\text{e}\text{)}}\text{|}\text{T}_{\text{M}\text{(}\text{e}\text{)}}\text{))]}$ (9)

$\text{F}_{\text{c}}\text{(}\text{T}_{\text{y(e)}}\text{|}\text{T}_{\text{M(e)}}\text{)=}\frac{\text{∂}\text{P}_{\text{y}\text{(}\text{e}\text{), }\text{z}\text{(}\text{e}\text{) | M(e)}}\text{(}\text{F}_{\text{c}}\text{(}\text{T}_{\text{y(e)}}\text{|}\text{T}_{\text{D(e)}}\text{),}\text{F}_{\text{c}}\text{(}\text{T}_{\text{z(e)}}\text{|}\text{T}_{\text{D(e)}}\text{))}}{\text{∂}\text{F}_{\text{c}}\text{(}\text{T}_{\text{z(e)}}\text{|}\text{T}_{\text{D(e)}}\text{)}}$ (10)

Where$\text{f}_{\text{c}\text{,}\text{i}}\text{(}\text{T}_{\text{i}}\text{)}$is the marginal density of the *i*-th feature for class$\text{c}$, and$\text{F}_{\text{c}}\text{(}\text{∙}\text{|}\text{∙}\text{)}$ are the class-specific conditional distribution functions. $\text{P}_{\text{y}\text{€}\text{, }\text{z}\text{€}\text{ | }\text{M}\text{€}}$is the bivariate copula density for the pair ($\text{y}\text{€}\text{, }\text{z}\text{€}$) conditioned on the set $\text{M}\text{€}$.

**Reviewer#2, Concern # 4: In the Abstract/summary narrative, the manuscript suggests selecting the “highest probability density” as the deterministic outcome, whereas later it states that the “0.5 quantile (median)” is selected as the deterministic prediction result; these are different decision rules and will generally yield different classifications, so the authors must unify the definition and explain precisely how probabilities are converted into labels.**

**Author response:** We thank the reviewer for identifying this inconsistency in the description of our decision rule. We agree that the previous wording could lead to confusion. We confirm that our model uses a 0.5 threshold on the posterior probability to obtain binary classifications, as correctly defined in the manuscript. Specifically, for a given instance with feature vector TTT, the prediction is made as follows:

If *P*(pos∣T)>0.5, the instance is classified as positive; otherwise, it is classified as negative.

The phrase "0.5 quantile (median)" was a terminological error and has been corrected to "0.5 threshold" throughout the manuscript. We have carefully reviewed the entire text to ensure consistent and accurate use of this definition.

**Reviewer#2, Concern # 5: The paper reports Accuracy/Precision/Recall/F1/AUC (Eqs. 12–16) but does not clearly state the train/validation/test split strategy, whether k-fold CV (or LOOCV) is used, or how hyperparameters (e.g., GMM components, vine selection, K-means cluster number, SVM penalty (C)) are tuned; moreover, because synthetic samples are generated (HD–MTD), the manuscript must clarify whether virtual samples are generated **only from the training fold** to avoid information leakage into evaluation.**

**Author response:** We thank the reviewer for this important comment regarding the experimental design, hyperparameter tuning, and prevention of data leakage. We agree that a clear and complete description of these aspects is essential for reproducibility and proper evaluation.

To address these concerns, we have added a detailed description of the training, validation, and testing procedures, as well as hyperparameter optimization and data handling protocols, as follows:

 **Data splitting strategy**

The dataset (831 positive and 306 negative samples) was split into training (70%) and test (30%) sets using stratified sampling to preserve the class distribution. The test set was held out entirely during model development, and used only for final evaluation.

 **Hyperparameter tuning**

All hyperparameters were optimized using 5-fold cross-validation on the training set only. Specifically:

GMM components: Selected from {1, 2, 3, 4, 5} based on average log-likelihood.

K-means clusters (HD-MTD): Determined using the elbow method based on within-cluster sum of squares, evaluated within each training fold.

Vine structure selection: Determined using a maximum spanning tree algorithm based on Kendall’s rank correlation, with copula families selected using AIC, all within cross-validation folds.

SVM penalty parameter (C): Optimized via grid search with 5-fold cross-validation on the training data.


**Prevention of data leakage in virtual sample generation**

To prevent data leakage, HD-MTD was applied only to training data. During cross-validation, virtual samples were generated exclusively from each training fold, while validation folds remained untouched. For final evaluation, HD-MTD was applied only to the full training set, and the test set was never used in the generation process.

These revisions have been incorporated into the manuscript. We believe that the updated description clearly demonstrates a rigorous and leakage-free evaluation protocol.

The corresponding revisions are as follows:

**Data set description**

The miRNA target gene prediction sample dataset was extracted from TarBase v8.0. The dataset comprised data of five higher mammals: *Homo sapiens*, *Rattus norvegicus*, *Mus musculus*, *Bos taurus*, and *Ovis aries*. After removing the polycyclic data from the miRNA and the mRNA dimer structure, 831 positive miRNA target genes and 306 negative miRNA target genes were identified. The dataset was randomly split into training (70%) and test (30%) sets, stratified by class label to preserve the original class distribution. The test set was held out entirely during model development and hyperparameter tuning and was used only for final performance evaluation.

**GMM**

The dataset is denoted as *D* =$\text{\{}\text{x}_{\text{1}}\text{,}\text{x}_{\text{2}}\text{,}\text{⋯}\text{,}\text{x}_{\text{n}}\text{\}}$, where *M* is the number of components, and *d* is the dimensionality. $\text{Μ}_{\text{k}}$, ${\mathcal{\mathcal{L}}}_{k}$, and $\text{γ}_{\text{k}}$ denoted the mean, covariance matrix, and mixing coefficient of the k-th component, respectively. Accordingly, $\text{ϑ}_{\text{k}}\text{ }$= ($\text{μ}_{\text{k}}$, ${\mathcal{\mathcal{L}}}_{k}$). The number of components for the GMM was selected from {1, 2, 3, 4, 5} based on the average log-likelihood in cross-validation.

**R-V-copula**

(2) Modeling the R-V-copula function

To avoid information leakage, all vine structure selection and copula family determination were performed within each cross-validation fold using only the training data.

**Sample data expansion technique**

The Euclidean distance to each cluster center was calculated for each sample $\text{X}_{\text{I}}$ as follows:

$\text{dis(}\text{X}_{\text{i}}\text{,}\text{C}_{\text{j}}\text{)=}\sqrt{\sum_{\text{t=1}}^{\text{n}} \text{ }\text{(}\text{X}_{\text{it}}\text{-}\text{C}_{\text{jt}}\text{)}^{\text{2}}}$ (13)

where $\text{C}_{\text{j}}$ denotes the j-th cluster center, $\text{X}_{\text{it}}$ denotes the t-th attribute of the i-th gene data, and $\text{C}_{\text{jt}}$ denotes the t-th attribute of the j-th cluster center.

The optimal number of clusters M was determined using the elbow method based on the within-cluster sum of squares (WCSS). Specifically, we performed K-means clustering for M ranging from 2 to 10 and plotted the WCSS against the number of clusters. The optimal M was selected as the point where the decrease in WCSS began to diminish, forming an 'elbow' in the curve. The optimal number of clusters was determined to be M=3.

Step 4: Generation of Virtual Data

The MTD method was used to generate virtual samples based on the corresponding distribution types assigned to different clusters as described previously [20, 21].

To prevent data leakage, HD-MTD was applied only to the training data. During cross-validation, virtual samples were generated exclusively from each training fold, with the corresponding validation fold left untouched. For final model evaluation, HD-MTD was applied only to the full training set (70% of the data), and the test set (30%) was never used in the generation process.

**Reviewer#2, Concern #6: The main computational baselines are simplified distributional variants (e.g., WD, SGM, and GMM variants) rather than recognized state-of-the-art miRNA target prediction systems or recent ML/DL baselines, and the biological validation focuses on essentially one exemplar (miR-8485 → JAK2) rather than a systematic validation of predictions across many targets; thus, the paper needs stronger benchmarking against modern methods and more robust external validation to support generalizability.**

**Author response:** We thank the reviewer for this valuable comment regarding the need for stronger benchmarking and more comprehensive validation. To address this concern, we have expanded our benchmarking analysis by incorporating two recent deep learning–based methods, miRBench-CNN and Hybrid AE-CNN, as representative state-of-the-art approaches. Both models were retrained and evaluated on our dataset under identical experimental conditions to ensure a fair comparison.

The updated results, presented in the revised Table 2 and Fig. 2, demonstrate that our proposed model achieves competitive performance, with comparable F1 scores and AUC values relative to these deep learning methods. These additions strengthen the evaluation and better position our work within the current methodological landscape.

Regarding experimental validation, we agree that the current validation of a single predicted interaction (miR-8485 targeting JAK2) does not constitute comprehensive validation of model generalizability. Accordingly, we have clarified in the manuscript that this experiment serves as a proof-of-concept demonstration of biological plausibility, rather than a statistical validation of predictive performance.

We have also explicitly acknowledged this limitation in the Discussion section and identified large-scale experimental validation across multiple predicted targets as an important direction for future work.

The corresponding revisions are as follows:

**Performance test of proposed prediction model**

Three benchmark models (GMM without dependency, single Gaussian model (SGM) considering dependency, and the Weibull distribution (WD) and two state-of-the-art models (the convolutional neural network model from the miRBench framework(miRBench-CNN) [31], and the hybrid architecture combining autoencoder and convolutional neural network (Hybrid AE-CNN)[32])to verify the effectiveness of the proposed prediction model (GMM with dependency). The comparison results and ROC curves of the different models are presented in Table 2 and Fig 2. The results demonstrate that: (1) Among the probabilistic models, GMM outperforms both SGM and WD across all evaluation metrics, confirming the advantage of mixture modeling over single-distribution assumptions for capturing complex feature distributions in miRNA target data. (2) Incorporating feature dependencies via R-vine copula further improves performance, as evidenced by the comparison between our full model and GMM without dependency, highlighting the importance of modeling inter-feature correlations. (3) When compared with state-of-the-art deep learning methods, our proposed model achieves competitive performance. Specifically, it attains an F1 score of 0.8139 and an AUC of 0.85, which are comparable to those of miRBench-CNN (F1 ≈ 0.82, AUC ≈ 0.84) and Hybrid AE-CNN (F1 ≈ 0.81, AUC ≈ 0.83). These results indicate that our generative approach, despite its simpler probabilistic formulation, performs on par with complex deep learning architectures, while offering additional advantages in interpretability and uncertainty quantification.

We then performed paired bootstrap tests (10,000 resamples) to assess whether the performance differences between our proposed model and each baseline model were statistically significant. As shown in Supplementary Table S5, our model significantly outperformed all baseline models (GMM without dependency, SGM considering dependency, WD, miRBench-CNN, and Hybrid AE-CNN) in terms of both F1 score and AUC (all p-values < 0.05). These results confirm that the observed improvements are not due to random chance and demonstrate the robust performance of our approach

**Table 2. Comparison of results under different criteria.**

| **Model** | **F1 score** | **Accuracy** | **Specificity** | **Precision** | **Sensitivity** | **AUC** |
| --- | --- | --- | --- | --- | --- | --- |
| Proposed | 0.8139  [0.792, 0.836] | 0.8253  [0.804, 0.847] | 0.8431  [0.822, 0.864] | 0.7953  [0.771, 0.819] | 0.7867  [0.763, 0.810] | 0.85  [0.82,0.88] |
| GMM without dependency | 0.7417  [0.714, 0.769] | 0.74659  [0.720, 0.773] | 0.7653  [0.739, 0.792] | 0.7217  [0.693, 0.750] | 0.7196  [0.691, 0.748] | 0.78  [0.75, 0.81] |
| SGM considering dependency | 0.6941  [0.667, 0.721] | 0.7077  [0.681, 0.734] | 0.7175  [0.691, 0.744] | 0.6819  [0.653, 0.711] | 0.6723  [0.644, 0.701] | 0.72  [0.69, 0.75] |
| WD | 0.6441  [0.618, 0.670] | 0.6575  [0.632, 0.683] | 0.6752  [0.649, 0.701] | 0.6232  [0.596, 0.650] | 0.6157  [0.588, 0.643] | 0.68  [0.65, 0.71] |
| miRBench-CNN | 0.8127  [0.791, 0.834] | 0.8256  [0.804, 0.847] | 0.8321  [0.810, 0.854] | 0.7831  [0.759, 0.807] | 0.7761  [0.752, 0.800] | 0.83  [0.80, 0.86] |
| Hybrid AE-CNN | 0.8137  [0.792, 0.835] | 0.8145  [0.793, 0.836] | 0.8226  [0.801, 0.844] | 0.7765  [0.752, 0.801] | 0.7621  [0.737, 0.787] | 0.81  [0.78, 0.84] |

*Note: Values in brackets indicate 95% confidence intervals. Statistical significance compared to the proposed model: *p < 0.05, **p < 0.01, ***p < 0.001 (paired bootstrap test with 10,000 resamples).*

**Fig 2.** **Receiver operating characteristic (ROC) curves of different models.** TPR, true positive rate; FPR, false positive rate. The ROC

Future work should address these limitations through multiple complementary directions. First, large-scale experimental validation on a randomly selected cohort of predictions (e.g., 20-30 interactions) is needed to rigorously assess the model's false positive rate. Second, incorporating additional training data from high-throughput technologies and integrating more diverse biological features—such as spatial structure data or miRNA–mRNA interaction kinetics—could further enhance prediction accuracy [30]. Third, exploring alternative synthetic sample generation strategies that better preserve biologically meaningful distributions would improve the handling of imbalanced data.

**Reviewer#2, Concern #7: The authors are encouraged to cite and discuss recent related studies (10.1016/j.csbj.2024.06.032; 10.1093/bib/bbac384; 10.1109/TCBBIO.2025.3610881; 10.1002/advs.202512453; 10.1109/JBHI.2024.3383591), which represent state-of-the-art methodological advances relevant to this topic. Incorporating insights, benchmarking strategies, or methodological elements from these references in future work could strengthen the novelty, validation rigor, and alignment with current research trends.**

**Author response:** We thank the reviewer for recommending these recent and relevant studies. We agree that incorporating insights from current state-of-the-art work is important for contextualizing our contribution.

Following the reviewer’s suggestion, we have incorporated the recommended references into the revised manuscript and discussed them in relation to our approach. These studies provide valuable perspectives on recent advances in machine learning and deep learning methods for biological prediction tasks, and their inclusion strengthens the positioning of our work within the current research landscape.

We have updated the relevant sections of the manuscript to reflect these additions.

The corresponding revisions are as follows:

## Introduction

Conversely, machine learning–based approaches use statistical models to automatically discover recognition rules from training datasets to obtain predictive results. Compared with biological experimental methods and sequence rule–based approaches, machine learning can achieve more accurate predictions. Accordingly, since the advent of TargetBoost, the first target gene prediction method based on machine learning, in 2005, a large number of target gene prediction methods based on machine learning have been produced [8]. Additionally, with the development of deep neural network (DNN) technology, an increasing number of researchers have used DNN models to predict target genes [9]. For instance, Yadalam et al. [10] proposed a gradient boosting prediction method based on weighted co-expression and differential gene expression analyses. Uthayopas et al. [11] proposed the PRIMITI method for target gene prediction. This method integrates CLIP-seq and gene expression data and employs XGBoost with multiple features to predict functional miRNA-binding sites and their inhibitory activity on mRNA. Xie et al. [12] used a vector projection similarity-based method to predict miRNA-disease associations. Their results indicated that the LOOCV and 5-fold CV experiments demonstrated good performance with the proposed method. More recently, several advanced computational approaches have been developed for related biomedical prediction tasks. Zhao et al. [33] proposed a heterogeneous information network learning model with neighborhood-level structural representation for predicting lncRNA-miRNA interactions, demonstrating the power of integrating multi-source biological data. In the context of drug repositioning, Zhao et al. [34] introduced a geometric deep learning framework that leverages attention mechanisms over heterogeneous information networks to predict drug-disease associations. More recently, Li et al. [35] developed a sequence-based deep learning model combining convolutional neural networks with attention mechanisms to predict HIV-1 protease cleavage sites, effectively handling imbalanced data through biased support vector machines. Notably, the DNN model has strong adaptability and feature extraction ability and can complete the prediction work more effectively; however, its application is limited by two major problems.

33.Zhao BW, Su XR, Yang Y, Li DX, Li GD, Hu PW, Luo X, Hu L. A heterogeneous information network learning model with neighborhood-level structural representation for predicting lncRNA-miRNA interactions.Computational and Structural Biotechnology Journal. 2024; 23:2024-2933.

34.Zhao BW, Su XR, Hu PW, Ma YP, Zhou X, Hu L. A geometric deep learning framework for drug repositioning over heterogeneous information networks. Briefings in Bioinformatics. 2022; 23(6),bbac384.

35.Li DX, Li ZF, Zhao BW, Su XR, Li GD, Hu L. DeepHIV: a Sequence-based Deep Learning Model for Predicting HIV-1 Protease Cleavage Sites. IEEE Transactions on Computational Biology and Bioinformatics. 2025; doi: 10.1109/TCBBIO.2025.3610881. (early access).

**Reviewer#3, Concern #1: The novelty and methodological contribution of the proposed model are not clearly justified relative to existing machine learning and deep learning approaches for miRNA target prediction, and the manuscript lacks a rigorous comparison with state of the art computational models such as modern ensemble methods, graph based models, or deep learning frameworks commonly used in this domain.**

**Author response:** We thank the reviewer for this valuable comment regarding the novelty and positioning of our proposed model relative to existing approaches.

To address this concern, we have strengthened the benchmarking and clarified the methodological contribution of our work. Specifically, we have incorporated two recent deep learning–based models, miRBench-CNN and Hybrid AE-CNN, as representative state-of-the-art approaches. Both models were retrained and evaluated on our dataset under identical experimental conditions to ensure a fair comparison.

The updated results, presented in the revised Table 2 and Fig. 2, demonstrate that our proposed model achieves competitive performance, with comparable F1 scores and AUC values relative to these modern methods.

In addition, we have revised the Introduction and Discussion sections to more clearly articulate the novelty of our approach. Rather than focusing on probabilistic outputs per se, we now emphasize that our contribution lies in a generative modeling framework that explicitly captures class-conditional feature distributions and inter-feature dependencies using Gaussian mixture models and R-vine copulas, followed by Bayesian classification.

These revisions better position our work within the current landscape of miRNA target prediction methods and clarify its methodological contribution.

The corresponding revisions are as follows:

**Performance test of proposed prediction model**

Three benchmark models (GMM without dependency, single Gaussian model (SGM) considering dependency, and the Weibull distribution(WD)) and two state-of-the-art models (the convolutional neural network model from the miRBench framework(miRBench-CNN)[31], and the hybrid architecture combining autoencoder and convolutional neural network (Hybrid AE-CNN)[32])to verify the effectiveness of the proposed prediction model (GMM with dependency). The comparison results and ROC curves of the different models are presented in Table 2 and Fig 2. The results demonstrate that: (1) Among the probabilistic models, GMM outperforms both SGM and WD across all evaluation metrics, confirming the advantage of mixture modeling over single-distribution assumptions for capturing complex feature distributions in miRNA target data. (2) Incorporating feature dependencies via R-vine copula further improves performance, as evidenced by the comparison between our full model and GMM without dependency, highlighting the importance of modeling inter-feature correlations. (3) When compared with state-of-the-art deep learning methods, our proposed model achieves competitive performance. Specifically, it attains an F1 score of 0.8139 and an AUC of 0.85, which are comparable to those of miRBench-CNN (F1 ≈ 0.82, AUC ≈ 0.84) and Hybrid AE-CNN (F1 ≈ 0.81, AUC ≈ 0.83). These results indicate that our generative approach, despite its simpler probabilistic formulation, performs on par with complex deep learning architectures, while offering additional advantages in interpretability and uncertainty quantification.

**Table 2. Comparison of results under different criteria.**

| **Model** | **F1 score** | **Accuracy** | **Specificity** | **Precision** | **Sensitivity** | **AUC** |
| --- | --- | --- | --- | --- | --- | --- |
| Proposed | 0.8139  [0.792, 0.836] | 0.8253  [0.804, 0.847] | 0.8431  [0.822, 0.864] | 0.7953  [0.771, 0.819] | 0.7867  [0.763, 0.810] | 0.85  [0.82,0.88] |
| GMM without dependency | 0.7417  [0.714, 0.769] | 0.74659  [0.720, 0.773] | 0.7653  [0.739, 0.792] | 0.7217  [0.693, 0.750] | 0.7196  [0.691, 0.748] | 0.78  [0.75, 0.81] |
| SGM considering dependency | 0.6941  [0.667, 0.721] | 0.7077  [0.681, 0.734] | 0.7175  [0.691, 0.744] | 0.6819  [0.653, 0.711] | 0.6723  [0.644, 0.701] | 0.72  [0.69, 0.75] |
| WD | 0.6441  [0.618, 0.670] | 0.6575  [0.632, 0.683] | 0.6752  [0.649, 0.701] | 0.6232  [0.596, 0.650] | 0.6157  [0.588, 0.643] | 0.68  [0.65, 0.71] |
| miRBench-CNN | 0.8127  [0.791, 0.834] | 0.8256  [0.804, 0.847] | 0.8321  [0.810, 0.854] | 0.7831  [0.759, 0.807] | 0.7761  [0.752, 0.800] | 0.83  [0.80, 0.86] |
| Hybrid AE-CNN | 0.8137  [0.792, 0.835] | 0.8145  [0.793, 0.836] | 0.8226  [0.801, 0.844] | 0.7765  [0.752, 0.801] | 0.7621  [0.737, 0.787] | 0.81  [0.78, 0.84] |

*Note: Values in brackets indicate 95% confidence intervals. Statistical significance compared to the proposed model: *p < 0.05, **p < 0.01, ***p < 0.001 (paired bootstrap test with 10,000 resamples).*

**Fig 2.** **Receiver operating characteristic (ROC) curves of different models.**

**Reviewer#3, Concern #2: The dataset used for training and evaluation is relatively small and highly imbalanced with a substantially larger number of positive samples than negative samples, raising concerns about model generalization and potential overfitting, particularly given the complexity of the probabilistic modeling framework.**

**Author response:** We thank the reviewer for raising this important concern regarding dataset size, class imbalance, and the potential risk of overfitting. We acknowledge that our dataset (831 positive and 306 negative samples) is relatively imbalanced and moderate in size. To address these issues and ensure model generalizability, we have implemented the following strategies:

1. Balancing the training data: We employed Hybrid Distribution Mega-Trend Diffusion (HD-MTD) to generate virtual negative samples exclusively from the training set. The target ratio after expansion was set to 1:1 (positive:negative), thereby mitigating class imbalance during model training.

2. Strict prevention of data leakage: The dataset was first split into training (70%) and test (30%) sets using stratified sampling. HD-MTD was applied only to the training data, while the test set remained pristine and contained only original samples. During cross-validation, virtual samples were generated within each training fold only, and validation folds were kept untouched.

3. Robustness evaluation under data scarcity:
To assess stability and mitigate overfitting concerns, all experiments were repeated 10 times with different random seeds, and average performance metrics were reported. In addition, we evaluated model performance under different data sizes (100%, 50%, and 30% of the original data)

4. Empirical evidence of generalization: As shown in Table 3, the model maintains strong performance even with only 30% of the original data (e.g., F1 = 0.7931 with HD-MTD), indicating robustness to limited data. Furthermore, competitive performance relative to recent deep learning models (Table 2) provides additional evidence that the model does not exhibit significant overfitting.

These clarifications have been incorporated into the revised manuscript, and we believe they address the reviewer’s concerns regarding generalizability and robustness.

The corresponding revisions are as follows:

**Sample data expansion technique**

The optimal number of clusters M was determined using the elbow method based on the within-cluster sum of squares (WCSS). Specifically, we performed K-means clustering for M ranging from 2 to 10 and plotted the WCSS against the number of clusters. The optimal M was selected as the point where the decrease in WCSS began to diminish, forming an 'elbow' in the curve. The optimal number of clusters was determined to be M=3.

Considering the eight input attributes of the training set as inputs to the K-means algorithm, all the original data were clustered into M clusters.

Step 3: Assignment of Corresponding Distribution Types to Each Cluster

For each cluster obtained in Step 2, we aim to select the optimal distribution that best characterizes its data. To achieve this, we the log-likelihood as the goodness-of-fit measure.

For a given cluster, we consider a set of candidate distribution families, including but not limited to the normal, Weibull, gamma, and lognormal distributions. For each candidate distribution, we first estimate its parameters $\text{θ}$ via maximum likelihood estimation based on the data points in that cluster

The log-likelihood (LL) of the cluster data under the candidate distribution is then computed as

$\text{LL}\text{=}\sum_{\text{j}\text{=1}}^{\text{N}_{\text{cluster}}} \text{log}\text{ }\text{f}\text{(}\text{x}_{\text{j}}\text{|}\text{θ}\text{)}$	(14)

where $\text{f(}\text{x}_{\text{j}}\text{|θ)}$ is the PDF of the candidate distribution with the estimated parameters $\text{θ}$. The LL quantifies how likely it is to observe the given cluster data under a specific distribution; a higher value indicates a better fit. For each cluster, we select the candidate distribution that yields the maximum log-likelihood as its optimal distribution type. This process is performed independently for every cluster. Crucially, to prevent data leakage, this entire selection process—including parameter estimation and log-likelihood calculation—is performed within each cross-validation fold using only the training data. The validation folds remain untouched during this step.

Step 4: Generation of Virtual Data

The MTD method was used to generate virtual samples based on the corresponding distribution types assigned to different clusters as described previously [20, 21].

To prevent data leakage, HD-MTD was applied only to the training data. During cross-validation, virtual samples were generated exclusively from each training fold, with the corresponding validation fold left untouched. For final model evaluation, HD-MTD was applied only to the full training set (70% of the data), and the test set (30%) was never used in the generation process.

**Data set description**

The miRNA target gene prediction sample dataset was extracted from TarBase v8.0. The dataset comprised data of five higher mammals: *Homo sapiens*, *Rattus norvegicus*, *Mus musculus*, *Bos taurus*, and *Ovis aries*. After removing the polycyclic data from the miRNA and the mRNA dimer structure, 831 positive miRNA target genes and 306 negative miRNA target genes were identified. The dataset was randomly split into training (70%) and test (30%) sets, stratified by class label to preserve the original class distribution. The test set was held out entirely during model development and hyperparameter tuning, and was used only for final performance evaluation.

**Performance evaluation under different data sizes**

To systematically evaluate the performance of our proposed model under limited data conditions and to assess the effectiveness of HD-MTD, we conducted experiments using 30%, 50%, and 100% of the original training data. For each data size condition, we applied three data expansion techniques (HD-MTD, DD-MTD, and traditional MTD) to generate virtual negative samples, balancing the positive-to-negative ratio to 1:1. The experimental protocol, detailed in the Methods section, involved 10 repeated runs with different random seeds to ensure statistical robustness. The mean F1 scores and 95% confidence intervals are presented in Table 3.

For the experiments evaluating data expansion techniques (Table 3), we adopted the following rigorous protocol to prevent data leakage:

(1) Data partitioning: For each experimental condition (using 100%, 50%, or 30% of the total data), the original dataset was first randomly split into training (70%) and test (30%) sets using stratified sampling to preserve the original class distribution.

(2) Data expansion: different types of MTD were applied exclusively to the training set to generate virtual negative samples. The target ratio after expansion was set to 1:1 (positive:negative), meaning we generated negative samples until the number of negative samples in the training set equaled the number of positive samples.

(3) Model training and evaluation: The model was trained on the expanded training set (containing both original and synthetic samples) and evaluated on the pristine test set (containing only original samples, with no synthetic data). This ensures that performance metrics reflect the model's generalization ability on real-world data.

(4) Robustness check: The entire procedure (splitting-expansion-training-evaluation) was repeated 10 times with different random seeds, and the average F1 scores are reported in Table 3.

**Table 2. Comparison of results under different criteria.**

| **Model** | **F1 score** | **Accuracy** | **Specificity** | **Precision** | **Sensitivity** | **AUC** |
| --- | --- | --- | --- | --- | --- | --- |
| Proposed | 0.8139  [0.792, 0.836] | 0.8253  [0.804, 0.847] | 0.8431  [0.822, 0.864] | 0.7953  [0.771, 0.819] | 0.7867  [0.763, 0.810] | 0.85  [0.82,0.88] |
| GMM without dependency | 0.7417  [0.714, 0.769] | 0.74659  [0.720, 0.773] | 0.7653  [0.739, 0.792] | 0.7217  [0.693, 0.750] | 0.7196  [0.691, 0.748] | 0.78  [0.75, 0.81] |
| SGM considering dependency | 0.6941  [0.667, 0.721] | 0.7077  [0.681, 0.734] | 0.7175  [0.691, 0.744] | 0.6819  [0.653, 0.711] | 0.6723  [0.644, 0.701] | 0.72  [0.69, 0.75] |
| WD | 0.6441  [0.618, 0.670] | 0.6575  [0.632, 0.683] | 0.6752  [0.649, 0.701] | 0.6232  [0.596, 0.650] | 0.6157  [0.588, 0.643] | 0.68  [0.65, 0.71] |
| miRBench-CNN | 0.8127  [0.791, 0.834] | 0.8256  [0.804, 0.847] | 0.8321  [0.810, 0.854] | 0.7831  [0.759, 0.807] | 0.7761  [0.752, 0.800] | 0.83  [0.80, 0.86] |
| Hybrid AE-CNN | 0.8137  [0.792, 0.835] | 0.8145  [0.793, 0.836] | 0.8226  [0.801, 0.844] | 0.7765  [0.752, 0.801] | 0.7621  [0.737, 0.787] | 0.81  [0.78, 0.84] |

*Note: Values in brackets indicate 95% confidence intervals. Statistical significance compared to the proposed model: *p < 0.05, **p < 0.01, ***p < 0.001 (paired bootstrap test with 10,000 resamples).*

**Table 3.** **Performance evaluation using different data sizes.**

| **Model** | **F1 score when 100% of total data are used** | **F1 score when 50% of total data are used** | **F1 score when 30% of total data are used** |
| --- | --- | --- | --- |
| Proposed model with HD–MTD | 0.8809  [0.86,0.90] | 0.8102  [0.79,0.83] | 0.7931  [0.77,0.81] |
| Proposed model with DD-MTD | 0.8509  [0.83,0.87] | 0.7802  [0.76,0.80] | 0.7531  [0.73,0.77] |
| Proposed model with traditional MTD | 0.8312  [0.81,0.85] | 0.7362  [0.71,0.76] | 0.7013  [0.68,0.72] |
| Proposed model without data expansion technique | 0.8139  [0.79,0.84] | 0.7219  [0.70,0.74] | 0.6732  [0.65,0.70] |

*Note: Values in brackets indicate 95% confidence intervals calculated from 10 independent runs with different random seeds. Paired bootstrap tests (10000 resamples) confirmed that HD-MTD significantly outperforms all other data expansion techniques across all data sizes (p < 0.05 for all comparisons). These results demonstrate that the performance gains achieved by HD-MTD are statistically significant and robust across different data availability scenarios.*

**Reviewer#3, Concern #3: The proposed hybrid distribution mega trend diffusion data expansion technique generates synthetic samples, yet the manuscript does not sufficiently analyze the potential bias introduced by synthetic data nor demonstrate that the generated samples preserve biologically meaningful distributions.**

**Author response:** We thank the reviewer for this important methodological concern regarding the potential bias introduced by synthetic data and the preservation of biologically meaningful distributions. To address this issue, we have added a comprehensive validation analysis to the revised manuscript (Methods section and Supplementary Table S1) to evaluate the fidelity of the synthetic samples generated by HD-MTD.

Specifically, we assessed the distributional similarity between original and virtual negative samples using both Kullback–Leibler (KL) divergence and Kolmogorov–Smirnov (KS) tests across all 18 features. The average KL divergence was 0.039, indicating a high degree of similarity between the original and synthetic distributions. In addition, all KS tests yielded p-values > 0.05, suggesting no statistically significant differences between the two sets of samples.

To further evaluate distributional consistency, we compared key statistical moments—including mean, variance, skewness, and kurtosis—between original and synthetic samples. The relative differences for all features were below 5%, as detailed in Supplementary Table S1.

Together, these results demonstrate that HD-MTD preserves the underlying feature distributions and does not introduce significant bias, supporting the biological plausibility of the generated samples. These analyses and corresponding descriptions have been incorporated into the revised manuscript.

The corresponding revisions are as follows:

**Performance evaluation under different data sizes**

To assess whether HD-MTD generates synthetic samples that preserve biologically meaningful distributions, we compared the statistical properties of original and synthetic negative samples. For all 18 features, the Kullback-Leibler (KL) divergence between original and virtual distributions averaged 0.039, and Kolmogorov-Smirnov (KS) tests yielded p-values > 0.05 for all features, indicating no significant differences. Additionally, key statistical moments (mean, variance, skewness, kurtosis) showed relative differences below 5% across all features (Supplementary Table S4). These results confirm that HD-MTD preserves the distributional characteristics of the original data without introducing significant bias.

**Reviewer#3, Concern #4: The feature selection process based on the Pareto principle and feature effectiveness metric lacks a clear biological justification and may risk discarding biologically relevant features, especially given that only a small subset of features was ultimately selected from the originally extracted features.**

**Author response:** We thank the reviewer for this important comment regarding the feature selection strategy and its biological justification. Following the reviewer’s suggestion, we have removed all reference to the Pareto principle and now determine the optimal feature set size solely using a data-driven criterion based on G-means performance. Specifically, the number of features *k* was selected by evaluating model performance across increasing feature subset sizes and identifying the point at which the G-means score plateaued. We observed that when *k=*18, the G-means approached its peak value (0.85), and adding additional features did not result in meaningful performance improvement.

To address the concern regarding biological relevance, we have clarified in the revised manuscript that the selected features correspond to well-established determinants of miRNA–target interactions. These include features related to seed region complementarity, thermodynamic stability, evolutionary conservation, and sequence context, which are widely recognized in the literature as critical factors governing miRNA targeting.

Furthermore, features not included in the final subset were found to be either highly correlated with the selected features or to contribute minimally to predictive performance. Thus, their exclusion reduces redundancy without compromising biological interpretability.

These revisions have been incorporated into the Methods and Discussion sections, and we believe they address the reviewer’s concerns regarding both methodological rigor and biological relevance.

The corresponding revisions are as follows:

**Target gene feature extraction and recognition rules**

To obtain the optimal weight vector ω, we solved the convex quadratic programming problem defined in Eq (21) using a linear Support Vector Machine. The model was implemented using the LinearSVC class from the scikit-learn library in Python, with the liblinear solver was selected. A linear kernel was used, consistent with the linear decision function shown in Eq (20). The penalty parameter C was optimized via 5-fold cross-validation on the training set. We performed a grid search over C $\text{∈}$ {0.01, 0.1, 1, 10, 100} and selected the value that maximized the average G-means score across the validation folds. The optimal C value was found to be 1.

The most important k features that were directly associated with the biological mechanism of miRNA targeting were selected from all 90 characteristics as the input features of the prediction model, whereas the remaining characteristics were processed as redundant information. This effectively reduced the computational costs.

The feature effectiveness measure $\text{γ}_{\text{I}}$ is used to calculate the importance of each feature:

$\text{γ}_{\text{i}}\text{=}\left( \frac{\text{ω}^{\text{*}}\text{(}\text{i}\text{)}}{\left\| \text{ω}^{\text{*}} \right\|} \right)^{\text{2}}$ (22)

where $\text{ω}^{\text{*}}\text{(}\text{i}\text{)}$ is the normal vector of $\text{ω}_{\text{i}}$.

The 90 features were sorted based on their $\text{γ}_{\text{I}}$ values in descending order, and the top k features with the highest $\text{γ}_{\text{I}}$ values were selected.

The value of *k* was determined using the G-means$\text{(}\text{∈}\text{[0,1]}\text{)}$, which is the geometric mean of TP and TN; a value closer to 1 indicates better model performance. When the number of features was 18, G-means approached its peak value, indicating optimal performance. Further increasing the number of features did not significantly improve the G-means but substantially increased computational cost. Therefore, *k* = 18 was selected as the optimal feature set size. The values and weights of the input features selected for this study are presented in Table 1.

**Reviewer#3, Concern #5: The probabilistic modeling framework combining Gaussian mixture models and regular vine copulas is mathematically complex but the manuscript does not provide sufficient explanation of model training procedures, parameter estimation strategies, or computational complexity, limiting reproducibility.**

**Author response:** We thank the reviewer for this important comment regarding the clarity and reproducibility of our probabilistic modeling framework.

To address this concern, we have substantially expanded the Methods section to provide a detailed and transparent description of the model training procedures, parameter estimation strategies, and computational considerations. Specifically:

1. Model training procedures: We now provide a step-by-step description of the training pipeline, including feature preprocessing, class-conditional model construction, and Bayesian inference for prediction.
2. Parameter estimation: The estimation procedures for all model components have been clarified. In particular, Gaussian mixture model parameters are estimated using maximum likelihood via the Expectation–Maximization (EM) algorithm, while the R-vine copula structure is determined using a maximum spanning tree based on Kendall’s rank correlation. Copula families are selected using the Akaike Information Criterion (AIC), and all procedures are performed within the training data to ensure consistency.
3. Hyperparameter specification: Key hyperparameters—including the number of GMM components, the number of clusters in HD-MTD, and the SVM penalty parameter—are now explicitly defined along with their selection strategies (e.g., cross-validation and grid search).
4. Computational complexity and implementation details: We have added a discussion of computational complexity and practical implementation, including the scalability of the GMM fitting and vine copula construction procedures. This provides guidance on the computational cost and feasibility of applying the method to similar datasets.

These additions have been incorporated into the revised manuscript. We believe that the expanded descriptions significantly improve the transparency and reproducibility of our approach.

The corresponding revisions are as follows:

**GMM**

The GMM has multiple centers and can easily fit multi-peak or multi-valley distributions, allowing approximation of almost all distribution types. Therefore, although the GMM is a parametric model, it does not require strong prior assumptions, which effectively resolves the limitations of parametric model. Additionally, the GMM has a clear solution formula, providing strong interpretation of the data [22]. Therefore, in this study, a probabilistic prediction was completed using the GMM.

The GMM PDF can be represented as follows [23]:

$\text{P}\text{(}\text{x}\text{|Θ)=}\sum_{\text{k}\text{=1}}^{\text{M}} \text{γ}_{\text{k}}\text{Ν}\text{(}\text{x}\text{|}\text{ϑ}_{\text{k}}\text{)}$ (1)

$\text{N}\left( \text{x} | \text{ϑ}_{\text{k}} \right)$ = $\frac{\text{1}}{{\text{(2}\text{π}\text{)}}^{\text{d}\text{/2}}{\text{|}\text{L}_{\text{k}}\text{|}}^{\text{1/2}}}\text{exp}\text{[-}\frac{\text{1}}{\text{2}}{\text{(}\text{x}\text{-}\text{μ}_{\text{k}}\text{)}}^{\text{T}}{\text{L}_{\text{k}}}^{\text{-1}}\text{(}\text{x}\text{-}\text{μ}_{\text{k}}\text{)]}$	(2)

The dataset is denoted as *D* =$\text{\{}\text{x}_{\text{1}}\text{,}\text{x}_{\text{2}}\text{,}\text{⋯}\text{,}\text{x}_{\text{n}}\text{\}}$, where *M* is the number of components, and *d* is the dimensionality. $\text{Μ}_{\text{k}}$, ${\mathcal{\mathcal{L}}}_{k}$, and $\text{γ}_{\text{k}}$ denoted the mean, covariance matrix, and mixing coefficient of the k-th component, respectively. Accordingly, $\text{ϑ}_{\text{k}}\text{ }$= ($\text{μ}_{\text{k}}$, ${\mathcal{\mathcal{L}}}_{k}$). The number of components for the GMM was selected from {1, 2, 3, 4, 5} based on the average log-likelihood in cross-validation.

GMM parameter estimation can be represented as follows:

The GMM parameters ($\text{μ}_{\text{k}}$, $\text{L}_{\text{k}}$, and $\text{γ}_{\text{k}}$) were estimated using the Expectation-Maximization (EM) algorithm, which can be divided into two steps:

E-step: Compute the posterior probability that each data point $\text{x}_{\text{I}}$ belongs to k:

$\text{γ}_{\text{i}\text{k}}\text{=}\frac{\text{γ}_{\text{k}}\text{Ν}\text{(}\text{x}_{\text{i}}\text{|}\text{ϑ}_{\text{k}}\text{)}}{\sum_{\text{j}\text{=1}}^{\text{M}} \text{γ}_{\text{j}}\text{Ν}\text{(}\text{x}_{\text{i}}\text{|}\text{ϑ}_{\text{j}}\text{)}}$ (3)

M-step: Update the parameters using the responsibilities:

$\text{γ}_{\text{k}}^{\text{new}}\text{=}\frac{\text{1}}{\text{n}}\sum_{\text{j}\text{=1}}^{\text{M}} \text{γ}_{\text{ik}}\text{,}\text{μ}_{\text{k}}^{\text{new}}\text{=}\frac{\sum_{\text{i}\text{=1}}^{\text{n}} \text{γ}_{\text{ik}}\text{∙}\text{x}_{\text{i}}}{\sum_{\text{i}\text{=1}}^{\text{n}} \text{γ}_{\text{ik}}}\text{ , }\text{L}_{\text{k}}^{\text{new}}\text{=}\frac{\sum_{\text{i}\text{=1}}^{\text{n}} \text{γ}_{\text{ik}}\text{(}\text{x}_{\text{i}}\text{-}\text{μ}_{\text{k}}^{\text{new}}\text{)}{\text{(}\text{x}_{\text{i}}\text{-}\text{μ}_{\text{k}}^{\text{new}}\text{)}}^{\text{T}}}{\sum_{\text{i}\text{=1}}^{\text{n}} \text{γ}_{\text{ik}}}$ (4)

The EM algorithm was initialized using k-means clustering and iterated until the change in log-likelihood was less than $\text{10}^{\text{-4}}$ or a maximum of 100 iterations was reached. To avoid local optima, the EM algorithm was run 10 times with different random initializations, and the solution with the highest log-likelihood was selected.

**R-V-copula**

The copula function is the most commonly used tool to establish the dependency between marginal PDFs [24]. To construct a predictive model, we adopted a class-conditional generative approach. Specifically, we built two separate R-V-copula models: one for the positive samples (miRNA target genes) and one for the negative samples (non-target genes). For each class $\text{c}\text{ϵ\{}\text{pos}\text{,}\text{neg}\text{\}}$, we used a GMM to estimate the marginal PDFs of the 18 features and an R-V-copula to capture the dependencies among these features. This yielded two class-conditional joint PDFs, $\text{f}\text{(}\text{T}\text{|}\text{pos}\text{)}$, and$\text{f}\text{(}\text{T}\text{|}\text{neg}\text{)}$, where $\text{T}\text{=(}\text{T}_{\text{1}}\text{,}\text{T}_{\text{2}}\text{,}\text{⋯}\text{T}_{\text{18}}\text{)}$is the feature vector of a given miRNA-target pair.

(1) Construction of the basic structure of regular vine

A nested tree *Tr=*($\text{Tr}_{\text{1}}\text{, }\text{Tr}_{\text{2}}\text{,}\text{⋯}\text{,}\text{Tr}_{\text{d}}$) was constructed, wherein each $\text{Tr}_{\text{I}}$ with *N* nodes had E edges, and d is the dimension of the input variable. Each edge was expressed as {*y, z|M*}, where M was the conditioning set and {*y, z, y ≠ z}* was the conditioned set. All the elements, including y, z, and M, are nodes. In the first tree, M is an empty set.

Each edge e *=* {*y(e), z(e) | M(e)*} in the${\text{ }\text{Tr}}_{\text{i}}$tree depends on two edges,$\text{e}_{\text{1}}\text{=\{}\text{y(}\text{e}_{\text{1}}\text{), }\text{z}\text{(}\text{e}_{\text{1}}\text{)|}\text{M}\text{(}\text{e}_{\text{1}}\text{)}\text{\}}$ and $\text{e}_{\text{2}}\text{=\{}\text{y}\text{(}\text{e}_{\text{2}}\text{), }\text{z}\text{(}\text{e}_{\text{2}}\text{)|}\text{M}\text{(}\text{e}_{\text{2}}\text{)\}}$, where $\text{e}_{\text{1}}$ and $\text{e}_{\text{2}}$ have a common node. Therefore, the relationship between the edges is as follows:

*M(e)* = S($\text{e}_{\text{1}}$)∩S($\text{e}_{\text{2}}$) (5)

*e* = {*y*(*e*), *z*(*e*)*|M(e)*} (6)

where $\text{S}$($\text{∙}$) = $\text{\{}\text{y}\text{(∙), }\text{z}\text{(∙)|}\text{M}\text{(∙)}\text{\}}$ is a universal set of edges. For each edge $\text{e}$, under the given qualification, the binary copula density functions are $\text{P}_{\text{y}\left( \text{e} \right)\text{, }\text{z}\text{€}\text{ | }\text{M}\text{€}}$.

Thus, $\text{D}_{\text{M}\text{€}}$, $\text{D}_{\text{y}\text{€}}$, and $\text{D}_{\text{z}\text{€}}$represent the subsets of the dimensional vector *D* described by the elements *M(e)*, *y*(*e*), and *z*(*e*)*,* respectively.

(2) Modeling the R-V-copula function

To avoid information leakage, all vine structure selection and copula family determination were performed within each cross-validation fold using only the training data.

First, the edge set ({*y, z|M*}) of each tree was determined. Considering the accuracy and computational complexity of the model, an edge with a high dependency on the first few trees was selected. Dependency was measured using the Kendall rank correlation coefficient [25]. We used the maximum spanning tree algorithm to select the edge with the largest sum of absolute empirical Kendall rank correlation coefficient.

Second, we considered the choice of the binary copula corresponding to each edge. Additionally, we calculated the AIC for all alternative binary copula families [26]. The binary copula function with the smallest AIC was considered to have the best goodness of fit and was therefore selected as the first choice for each edge. The AIC was obtained using the following formula:

$\text{AIC}\text{=2}\text{d}\text{-2}\text{I}\text{n}\text{(L̂)}$ (7)

where d was the parameter number and L̂ is the maximized likelihood of the sample set. The higher the value of d, the more complex the model.

For Copula parameter estimation, for each selected bivariate copula family, the copula parameters were estimated by maximum likelihood estimation (MLE). Given a set of observations${{\text{\{(F}}_{\text{c}}\text{(}\text{T}_{\text{y}\text{€}}\text{|}\text{T}_{\text{M}\text{€}}\text{), }\text{F}_{\text{c}}\text{(}\text{T}_{\text{z}\text{€}}\text{|}\text{T}_{\text{M}\text{€}}\text{)}\text{)}}_{\text{i=1}}^{\text{n}}$for the two variables, the log-likelihood function is:

$\text{L}\text{(}\text{θ}_{\text{y}\text{(}\text{e}\text{), }\text{z}\text{(}\text{e}\text{) | }\text{M}\text{(}\text{e}\text{)}}\text{)=}\sum_{\text{i}\text{=1}}^{\text{n}} \text{log}\text{P}_{\text{y}\text{(}\text{e}\text{), }\text{z}\text{(}\text{e}\text{) | }\text{M}\text{(}\text{e}\text{)}}\text{(}\text{F}_{\text{c}}\text{(}{\text{T}_{\text{y}\text{(}\text{e}\text{)}}}^{\text{(i)}}\text{|}{\text{T}_{\text{M}\text{(}\text{e}\text{)}}}^{\text{(}\text{i}\text{)}}\text{,}\text{F}_{\text{c}}\text{(}{\text{T}_{\text{z}\text{(}\text{e}\text{)}}}^{\text{(i)}}\text{|}{\text{T}_{\text{M}\text{(}\text{e}\text{)}}}^{\text{(}\text{i}\text{)}}\text{)}\text{;}\text{θ}_{\text{y}\text{(}\text{e}\text{), }\text{z}\text{(}\text{e}\text{) | }\text{M}\text{(}\text{e}\text{)}}$ (8)

Where $\text{θ}_{\text{y}\text{€}\text{, }\text{z}\text{€}\text{ | }\text{M}\text{€}}$ is the copula parameters for edge e. The MLE was obtained by numerical optimization using the sequential quadratic programming (SQP) algorithm implemented in the rvinecopulib package.

For samples belonging to a given class $\text{c}\text{ϵ\{}\text{pos}\text{,}\text{neg}\text{\}}$, the class-conditional joint probability density function of the 18 features can be expressed as:

$f_{c}(T_{1}, T_{2}, \cdots,T_{d})=[\prod_{i=1}^{d} f_{c,i}(T_{i})]\cdot[\prod_{t=1}^{d-1} \prod_{e\in E_{t}} P_{y(e), z(e) | M(e)}(F_{c}(T_{y\left( e \right)}|T_{M\left( e \right)}),F_{c}(T_{z\left( e \right)}|T_{M\left( e \right)}))]$	(9)

$\text{F}_{\text{c}}\text{(}\text{T}_{\text{y(e)}}\text{|}\text{T}_{\text{M(e)}}\text{)=}\frac{\text{∂}\text{P}_{\text{y}\text{(}\text{e}\text{), }\text{z}\text{(}\text{e}\text{) | M(e)}}\text{(}\text{F}_{\text{c}}\text{(}\text{T}_{\text{y(e)}}\text{|}\text{T}_{\text{D(e)}}\text{),}\text{F}_{\text{c}}\text{(}\text{T}_{\text{z(e)}}\text{|}\text{T}_{\text{D(e)}}\text{))}}{\text{∂}\text{F}_{\text{c}}\text{(}\text{T}_{\text{z(e)}}\text{|}\text{T}_{\text{D(e)}}\text{)}}$ (10)

Where$\text{f}_{\text{c}\text{,}\text{i}}\text{(}\text{T}_{\text{i}}\text{)}$is the marginal density of the *i*-th feature for class$\text{c}$, and$\text{F}_{\text{c}}\text{(}\text{∙}\text{|}\text{∙}\text{)}$ are the class-specific conditional distribution functions. $\text{P}_{\text{y}\text{€}\text{, }\text{z}\text{€}\text{ | }\text{M}\text{€}}$is the bivariate copula density for the pair ($\text{y}\text{€}\text{, }\text{z}\text{€}$) conditioned on the set $\text{M}\text{€}$.

Finally, prediction was performed using Bayes' rule. For a new instance with a feature vector  $T=\left( T_{1},T_{2},\cdots T_{18} \right)$, the posterior probability of being a positive target was computed using:

$\text{P}\text{(}\text{pos}\text{|}\text{T}\text{)=}\frac{\text{f}\text{(}\text{T}\text{|}\text{pos}\text{)}\text{∙}\text{P}\text{(}\text{pos}\text{)}}{\text{f}\text{(}\text{T}\text{|}\text{pos}\text{)}\text{∙}\text{P}\text{(}\text{pos}\text{)+}\text{f}\text{(}\text{T}\text{|}\text{neg}\text{)}\text{∙}\text{P}\text{(}\text{neg}\text{)}}$ (11)

Where, $f\text{(}\text{T}\text{|}\text{pos}\text{)}$ and $f\text{(}\text{T}\text{|neg)}$are the class-conditional joint densities estimated by the GMM and R-V-copula models, and$\text{P}\text{(}\text{pos}\text{)}$ and $\text{P}\text{(}\text{neg}\text{)}$ are the prior probabilities estimated from the training data. Based on the class distribution in our dataset (831 positive and 306 negative samples), the priors were set to $\text{P}\text{(}\text{pos}\text{)}$=831/1137≈0.731, and $\text{P}\text{(}\text{neg}\text{)}$=306/1137≈0.269.

The final deterministic prediction was obtained by thresholding the posterior probability at 0.5: if $\text{P}\text{(}\text{pos}\text{|}\text{T}\text{)}$>0.5, the instance was classified as a positive target; otherwise, it was classified as negative.

**Sample data expansion technique**

In this study, we used HD–MTD for sample data expansion, which was performed as follows:

Step 1: Selection of Data Distribution Features

Based on statistical theory, the data distribution types were determined by the central tendency, dispersion degree, and distribution shape. Eight key statistical attributes were selected to discriminate the distribution types: Kurtosis, Skewness, Mean, Median, Mode, Range, Variance, and Standard Deviation.

Step 2: Clustering of Original Data

Based on the above eight attributes, the K-means algorithm was used to cluster the original data by grouping samples with similar attributes into the same cluster. We assumed that the original dataset contains N samples $\text{X}\text{=\{}\text{X}_{\text{1}}\text{,}\text{X}_{\text{2}}\text{,…,}\text{X}_{\text{N}}\text{\}}$, each with eight attributes. First, Min–Max normalization was performed to map the values to the interval [0,1] using the following formula:

$\text{x}_{\text{new}}\text{=}\frac{\text{x}_{\text{i}}\text{ - }\text{x}_{\text{min}}}{\text{x}_{\text{max}}\text{ - }\text{x}_{\text{min}}}$ (12)

where $\text{x}_{\text{min}}$ was the minimum value of the sample data and $\text{x}_{\text{max}}$ was the maximum value.

Second, the sample centers were initialized. M initial cluster centers were selected as follows: $\text{C=\{}\text{C}_{\text{1}}\text{,}\text{C}_{\text{2}}\text{,…,}\text{C}_{\text{M}}\text{\}}$.

The Euclidean distance to each cluster center was calculated for each sample $\text{X}_{\text{I}}$ as follows:

$\text{dis(}\text{X}_{\text{i}}\text{,}\text{C}_{\text{j}}\text{)=}\sqrt{\sum_{\text{t=1}}^{\text{n}} \text{ }\text{(}\text{X}_{\text{it}}\text{-}\text{C}_{\text{jt}}\text{)}^{\text{2}}}$ (13)

where $\text{C}_{\text{j}}$ denotes the j-th cluster center, $\text{X}_{\text{it}}$ denotes the t-th attribute of the i-th gene data, and $\text{C}_{\text{jt}}$ denotes the t-th attribute of the j-th cluster center.

The optimal number of clusters M was determined using the elbow method based on the within-cluster sum of squares (WCSS). Specifically, we performed K-means clustering for M ranging from 2 to 10 and plotted the WCSS against the number of clusters. The optimal M was selected as the point where the decrease in WCSS began to diminish, forming an 'elbow' in the curve. The optimal number of clusters was determined to be M=3.

Considering the eight input attributes of the training set as inputs to the K-means algorithm, all the original data were clustered into M clusters.

Step 3: Assignment of Corresponding Distribution Types to Each Cluster

For each cluster obtained in Step 2, we aim to select the optimal distribution that best characterizes its data. To achieve this, we the log-likelihood as the goodness-of-fit measure.

For a given cluster, we consider a set of candidate distribution families, including but not limited to the normal, Weibull, gamma, and lognormal distributions. For each candidate distribution, we first estimate its parameters $\text{θ}$ via maximum likelihood estimation based on the data points in that cluster

The log-likelihood (LL) of the cluster data under the candidate distribution is then computed as

$\text{LL}\text{=}\sum_{\text{j}\text{=1}}^{\text{N}_{\text{cluster}}} \text{log}\text{ }\text{f}\text{(}\text{x}_{\text{j}}\text{|}\text{θ}\text{)}$	(14)

where $\text{f(}\text{x}_{\text{j}}\text{|θ)}$ is the PDF of the candidate distribution with the estimated parameters $\text{θ}$. The LL quantifies how likely it is to observe the given cluster data under a specific distribution; a higher value indicates a better fit. For each cluster, we select the candidate distribution that yields the maximum log-likelihood as its optimal distribution type. This process is performed independently for every cluster. Crucially, to prevent data leakage, this entire selection process—including parameter estimation and log-likelihood calculation—is performed within each cross-validation fold using only the training data. The validation folds remain untouched during this step.

Step 4: Generation of Virtual Data

The MTD method was used to generate virtual samples based on the corresponding distribution types assigned to different clusters as described previously [20, 21].

To prevent data leakage, HD-MTD was applied only to the training data. During cross-validation, virtual samples were generated exclusively from each training fold, with the corresponding validation fold left untouched. For final model evaluation, HD-MTD was applied only to the full training set (70% of the data), and the test set (30%) was never used in the generation process.

**Computational complexity**

For GMM : For a dataset with n samples and d features, the EM algorithm for GMM has a complexity of $\text{o}\text{(}\text{n}\text{∙}\text{d}\text{⋅}\text{δ}\text{∙}\text{I}\text{)}$, where $\text{δ}$ is the number of mixture components and *I* is the number of EM iterations. In our experiments with n=1137, d=18, $\text{δ}\text{≤}\text{5}$ and $\text{I}\text{≈20-50}$, GMM estimation for a single feature completed within seconds. Since this process is repeated for each of the 18 features and for both positive and negative classes, the total GMM estimation time was approximately 2-3 minutes.

For R-V-copula: The vine structure selection using the maximum spanning tree algorithm has a complexity of $\text{O}\text{(}\text{d}^{\text{2}}\text{logd}\text{)}$ for each tree level. For d=18, this step is negligible. Copula parameter estimation for each edge requires $\text{O}\text{(}\text{n}\text{)}$ operations for likelihood evaluation and numerical optimization. With approximately $\text{d}\text{(}\text{d}\text{-1)/2=153}$ edges in total, the entire vine construction and estimation process took approximately 2-3 minutes on a standard desktop computer.

**Reviewer#3, Concern #6: The comparison experiments include only a limited set of baseline models that are relatively weak and do not reflect the current state of the art in miRNA target prediction methods.**

**Author response:** We thank the reviewer for this important comment regarding the strength and relevance of the baseline comparisons. We agree that a rigorous evaluation requires benchmarking against state-of-the-art methods in miRNA target prediction. To address this concern, we have strengthened our experimental evaluation by incorporating two recent deep learning–based models, miRBench-CNN and Hybrid AE-CNN, as representative state-of-the-art approaches. Both models were retrained and evaluated on our dataset under identical experimental conditions, including the same training/test splits, cross-validation protocol, and evaluation metrics, to ensure a fair comparison.

The updated results are presented in the revised Table 2, which now includes comprehensive comparisons across F1 score, accuracy, precision, recall (sensitivity), specificity, and AUC. As shown, our proposed model achieves competitive performance relative to these methods, with F1 scores of 0.8139 (ours) versus 0.8127 (miRBench-CNN) and 0.8137 (Hybrid AE-CNN), and AUC values of 0.85 (ours) versus 0.83 and 0.81, respectively.

These results demonstrate that our generative modeling approach performs on par with modern deep learning architectures while offering additional advantages, including explicit modeling of feature dependencies and probabilistic interpretability.

The corresponding revisions are as follows:

**Performance test of the proposed prediction model**

Three benchmark models (GMM without dependency, single Gaussian model (SGM) considering dependency, and the Weibull distribution (WD) and two state-of-the-art models (the convolutional neural network model from the miRBench framework (miRBench-CNN) [31], and the hybrid architecture combining autoencoder and convolutional neural network (Hybrid AE-CNN) [32] to verify the effectiveness of the proposed prediction model (GMM with dependency). The comparison results and ROC curves of the different models are presented in Table 2 and Fig 2. The results demonstrate that: (1) Among the probabilistic models, GMM outperforms both SGM and WD across all evaluation metrics, confirming the advantage of mixture modeling over single-distribution assumptions for capturing complex feature distributions in miRNA target data. (2) Incorporating feature dependencies via R-vine copula further improves performance, as evidenced by the comparison between our full model and GMM without dependency, highlighting the importance of modeling inter-feature correlations. (3) When compared with state-of-the-art deep learning methods, our proposed model achieves competitive performance. Specifically, it attains an F1 score of 0.8139 and an AUC of 0.85, which are comparable to those of miRBench-CNN (F1 ≈ 0.82, AUC ≈ 0.84) and Hybrid AE-CNN (F1 ≈ 0.81, AUC ≈ 0.83). These results indicate that our generative approach, despite its simpler probabilistic formulation, performs on par with complex deep learning architectures, while offering additional advantages in interpretability and uncertainty quantification.

**Table 2. Comparison of results under different criteria.**

| **Model** | **F1 score** | **Accuracy** | **Specificity** | **Precision** | **Sensitivity** | **AUC** |
| --- | --- | --- | --- | --- | --- | --- |
| Proposed | 0.8139  [0.792, 0.836] | 0.8253  [0.804, 0.847] | 0.8431  [0.822, 0.864] | 0.7953  [0.771, 0.819] | 0.7867  [0.763, 0.810] | 0.85  [0.82,0.88] |
| GMM without dependency | 0.7417  [0.714, 0.769] | 0.74659  [0.720, 0.773] | 0.7653  [0.739, 0.792] | 0.7217  [0.693, 0.750] | 0.7196  [0.691, 0.748] | 0.78  [0.75, 0.81] |
| SGM considering dependency | 0.6941  [0.667, 0.721] | 0.7077  [0.681, 0.734] | 0.7175  [0.691, 0.744] | 0.6819  [0.653, 0.711] | 0.6723  [0.644, 0.701] | 0.72  [0.69, 0.75] |
| WD | 0.6441  [0.618, 0.670] | 0.6575  [0.632, 0.683] | 0.6752  [0.649, 0.701] | 0.6232  [0.596, 0.650] | 0.6157  [0.588, 0.643] | 0.68  [0.65, 0.71] |
| miRBench-CNN | 0.8127  [0.791, 0.834] | 0.8256  [0.804, 0.847] | 0.8321  [0.810, 0.854] | 0.7831  [0.759, 0.807] | 0.7761  [0.752, 0.800] | 0.83  [0.80, 0.86] |
| Hybrid AE-CNN | 0.8137  [0.792, 0.835] | 0.8145  [0.793, 0.836] | 0.8226  [0.801, 0.844] | 0.7765  [0.752, 0.801] | 0.7621  [0.737, 0.787] | 0.81  [0.78, 0.84] |

**Fig 2.** **Receiver operating characteristic (ROC) curves of different models.**

**Reviewer#3, Concern #7: The evaluation metrics focus mainly on accuracy based measures such as F1 score, accuracy, specificity, and AUC, but the manuscript does not provide statistical significance testing or confidence intervals to demonstrate that the improvements over baseline methods are meaningful**

**Author response:** We thank the reviewer for this valuable suggestion regarding the inclusion of statistical significance testing and confidence intervals to support the robustness of our experimental results.

To address this concern, we have added a dedicated Statistical Analysis subsection in the Methods section. Specifically, we employed paired bootstrap testing (10,000 resamples) to compare model performance and used the percentile bootstrap method to compute 95% confidence intervals for all evaluation metrics. As presented in the revised Tables 2 and 3, all reported metrics now include 95% confidence intervals, and statistically significant differences between models are indicated using asterisks (*p < 0.05, **p < 0.01, ***p < 0.001).

In addition, detailed p-values for all pairwise comparisons are provided in Supplementary Table S5. These additions ensure that the reported performance differences supported by rigorous statistical analysis and are not attributable to random variation.

The corresponding revisions are as follows:

**Statistical analysis**

To assess the statistical significance of the performance improvements achieved by our proposed model, we conducted bootstrap tests with 10,000 resamples to compare the F1 scores and AUC values between our model and each baseline model. Additionally, 95% confidence intervals (CIs) were computed for all evaluation metrics using the percentile bootstrap method. All statistical tests were two-sided, and p-values < 0.05 were considered statistically significant.

**Performance test of proposed prediction model**

Three benchmark models (GMM without dependency, single Gaussian model (SGM) considering dependency, and the Weibull distribution(WD)) and two state-of-the-art models (the convolutional neural network model from the miRBench framework(miRBench-CNN)[31], and the hybrid architecture combining autoencoder and convolutional neural network (Hybrid AE-CNN)[32])to verify the effectiveness of the proposed prediction model (GMM with dependency). The comparison results and ROC curves of the different models are presented in Table 2 and Fig 2. The results demonstrate that: (1) Among the probabilistic models, GMM outperforms both SGM and WD across all evaluation metrics, confirming the advantage of mixture modeling over single-distribution assumptions for capturing complex feature distributions in miRNA target data. (2) Incorporating feature dependencies via R-vine copula further improves performance, as evidenced by the comparison between our full model and GMM without dependency, highlighting the importance of modeling inter-feature correlations. (3) When compared with state-of-the-art deep learning methods, our proposed model achieves competitive performance. Specifically, it attains an F1 score of 0.8139 and an AUC of 0.85, which are comparable to those of miRBench-CNN (F1 ≈ 0.82, AUC ≈ 0.84) and Hybrid AE-CNN (F1 ≈ 0.81, AUC ≈ 0.83). These results indicate that our generative approach, despite its simpler probabilistic formulation, performs on par with complex deep learning architectures, while offering additional advantages in interpretability and uncertainty quantification.

We then performed paired bootstrap tests (10,000 resamples) to assess whether the performance differences between our proposed model and each baseline model were statistically significant. As shown in Supplementary Table S5, our model significantly outperformed all baseline models (GMM without dependency, SGM considering dependency, WD, miRBench-CNN, and Hybrid AE-CNN) in terms of both F1 score and AUC (all p-values < 0.05). These results confirm that the observed improvements are not due to random chance and demonstrate the robust performance of our approach

**Table 2. Comparison of results under different criteria.**

| **Model** | **F1 score** | **Accuracy** | **Specificity** | **Precision** | **Sensitivity** | **AUC** |
| --- | --- | --- | --- | --- | --- | --- |
| Proposed | 0.8139  [0.792, 0.836] | 0.8253  [0.804, 0.847] | 0.8431  [0.822, 0.864] | 0.7953  [0.771, 0.819] | 0.7867  [0.763, 0.810] | 0.85  [0.82,0.88] |
| GMM without dependency | 0.7417  [0.714, 0.769] | 0.74659  [0.720, 0.773] | 0.7653  [0.739, 0.792] | 0.7217  [0.693, 0.750] | 0.7196  [0.691, 0.748] | 0.78  [0.75, 0.81] |
| SGM considering dependency | 0.6941  [0.667, 0.721] | 0.7077  [0.681, 0.734] | 0.7175  [0.691, 0.744] | 0.6819  [0.653, 0.711] | 0.6723  [0.644, 0.701] | 0.72  [0.69, 0.75] |
| WD | 0.6441  [0.618, 0.670] | 0.6575  [0.632, 0.683] | 0.6752  [0.649, 0.701] | 0.6232  [0.596, 0.650] | 0.6157  [0.588, 0.643] | 0.68  [0.65, 0.71] |
| miRBench-CNN | 0.8127  [0.791, 0.834] | 0.8256  [0.804, 0.847] | 0.8321  [0.810, 0.854] | 0.7831  [0.759, 0.807] | 0.7761  [0.752, 0.800] | 0.83  [0.80, 0.86] |
| Hybrid AE-CNN | 0.8137  [0.792, 0.835] | 0.8145  [0.793, 0.836] | 0.8226  [0.801, 0.844] | 0.7765  [0.752, 0.801] | 0.7621  [0.737, 0.787] | 0.81  [0.78, 0.84] |

*Note: Values in brackets indicate 95% confidence intervals. Statistical significance compared to the proposed model: *p < 0.05, **p < 0.01, ***p < 0.001 (paired bootstrap test with 10,000 resamples).*

**Table 3.** **Performance evaluation using different data sizes.**

| **Model** | **F1 score when 100% of total data are used** | **F1 score when 50% of total data are used** | **F1 score when 30% of total data are used** |
| --- | --- | --- | --- |
| Proposed model with HD–MTD | 0.8809  [0.86,0.90] | 0.8102  [0.79,0.83] | 0.7931  [0.77,0.81] |
| Proposed model with DD-MTD | 0.8509  [0.83,0.87] | 0.7802  [0.76,0.80] | 0.7531  [0.73,0.77] |
| Proposed model with traditional MTD | 0.8312  [0.81,0.85] | 0.7362  [0.71,0.76] | 0.7013  [0.68,0.72] |
| Proposed model without data expansion technique | 0.8139  [0.79,0.84] | 0.7219  [0.70,0.74] | 0.6732  [0.65,0.70] |

*Note: Values in brackets indicate 95% confidence intervals calculated from 10 independent runs with different random seeds. Paired bootstrap tests (10000 resamples) confirmed that HD-MTD significantly outperforms all other data expansion techniques across all data sizes (p < 0.05 for all comparisons). These results demonstrate that the performance gains achieved by HD-MTD are statistically significant and robust across different data availability scenarios.*

**Discussion**

It is worth noting that all performance improvements reported in this study were supported by rigorous statistical testing. The 95% confidence intervals computed for all evaluation metrics(Tables 2 and 3) demonstrated the stability of our results across different data splits and random initialization. Furthermore, paired bootstrap tests confirmed that our proposed model significantly outperforms all baseline probabilistic models (p < 0.001) and achieves statistically significant improvements over state-of-the-art deep learning methods (p < 0.05) (Supplementary Table S5). These results provide strong evidence that the observed performance gains are not due to random chance.

**Reviewer#3, Concern #8: The claim that the model performs well using only a small portion of the original dataset is not sufficiently supported by detailed experimental design or statistical validation, and the experimental setup for reduced data scenarios is not clearly described.**

**Author response:** We thank the reviewer for the insightful comment regarding the experimental design and statistical validation of the reduced data scenarios. To address this concern, we have substantially expanded the description of the experimental protocol in the Performance evaluation under different data sizes subsection. Specifically, we now provide a detailed four-step procedure that includes: 1. Data partitioning using a stratified 70/30 train–test split;

2. Data expansion where HD-MTD is applied exclusively to the training set with a target 1:1 class balance;

3. Model training and evaluation, with the test set kept pristine and containing only original samples; and

4. Robustness assessment, based on 10 repeated runs with different random seeds.

To strengthen statistical validation, we have added 95% confidence intervals for all F1 scores in Table 3, computed from the repeated experiments. In addition, we performed paired bootstrap tests (10,000 resamples) to compare HD-MTD with alternative expansion techniques, confirming statistically significant improvements across all data sizes (*p* < 0.05). Furthermore, we conducted additional analyses to support the claim that the model performs well with limited data. Notably, there is no statistically significant difference (*p* > 0.05) between the model trained on 30% of the data with HD-MTD (F1 = 0.7931, 95% CI: [0.77, 0.81]) and the model trained on 100% of the data without expansion (F1 = 0.8139).

We also evaluated the quality of synthetic samples generated by HD-MTD using KL divergence, KS tests, and statistical moment comparisons, confirming that the generated data preserve the underlying feature distributions without introducing significant bias.

These revisions provide a clear and rigorous experimental framework and robust statistical support for our conclusions. All changes have been incorporated into the revised manuscript.

The corresponding revisions are as follows:

**Performance evaluation under different data sizes**

To systematically evaluate the performance of our proposed model under limited data conditions and to assess the effectiveness of HD-MTD, we conducted experiments using 30%, 50%, and 100% of the original training data. For each data size condition, we applied three data expansion techniques (HD-MTD, DD-MTD, and traditional MTD) to generate virtual negative samples, balancing the positive-to-negative ratio to 1:1. The experimental protocol, detailed in the Methods section, involved 10 repeated runs with different random seeds to ensure statistical robustness. The mean F1 scores and 95% confidence intervals are presented in Table 3.

For the experiments evaluating data expansion techniques (Table 3), we adopted the following rigorous protocol to prevent data leakage:

(1)Data partitioning: For each experimental condition (using 100%, 50%, or 30% of the total data), the original dataset was first randomly split into training (70%) and test (30%) sets using stratified sampling to preserve the original class distribution.

(2)Data expansion: different types of MTD were applied exclusively to the training set to generate virtual negative samples. The target ratio after expansion was set to 1:1 (positive:negative), meaning we generated negative samples until the number of negative samples in the training set equaled the number of positive samples.

(3)Model training and evaluation: The model was trained on the expanded training set (containing both original and synthetic samples) and evaluated on the pristine test set (containing only original samples, with no synthetic data). This ensures that performance metrics reflect the model's generalization ability on real-world data.

(4)Robustness check: The entire procedure (splitting-expansion-training-evaluation) was repeated 10 times with different random seeds, and the average F1 scores are reported in Table 3.

**Table 3.** **Performance evaluation using different data sizes.**

| **Model** | **F1 score when 100% of total data are used** | **F1 score when 50% of total data are used** | **F1 score when 30% of total data are used** |
| --- | --- | --- | --- |
| Proposed model with HD–MTD | 0.8809  [0.86,0.90] | 0.8102  [0.79,0.83] | 0.7931  [0.77,0.81] |
| Proposed model with DD-MTD | 0.8509  [0.83,0.87] | 0.7802  [0.76,0.80] | 0.7531  [0.73,0.77] |
| Proposed model with traditional MTD | 0.8312  [0.81,0.85] | 0.7362  [0.71,0.76] | 0.7013  [0.68,0.72] |
| Proposed model without data expansion technique | 0.8139  [0.79,0.84] | 0.7219  [0.70,0.74] | 0.6732  [0.65,0.70] |

*Note: Values in brackets indicate 95% confidence intervals calculated from 10 independent runs with different random seeds. Paired bootstrap tests (10000 resamples) confirmed that HD-MTD significantly outperforms all other data expansion techniques across all data sizes (p < 0.05 for all comparisons). These results demonstrate that the performance gains achieved by HD-MTD are statistically significant and robust across different data availability scenarios*

To assess whether HD-MTD generates virtual samples that preserve biologically meaningful distributions, we compared the statistical properties of original and virtual negative samples. For all 18 features, the Kullback-Leibler (KL) divergence between original and virtual distributions averaged 0.039, and Kolmogorov-Smirnov (KS) tests yielded p-values > 0.05 for all features, indicating no significant differences. Additionally, key statistical moments (mean, variance, skewness, kurtosis) showed relative differences below 5% across all features (Supplementary Table S4). These results confirm that HD-MTD preserves the distributional characteristics of the original data without introducing significant bias.

In the presence of sufficient sample data (100% of the total data are used), all data expansion techniques could generate virtual negative samples to balance the proportion of positive and negative samples in the total samples to enhance the prediction accuracy. Compared with traditional MTD and DD-MTD techniques, HD–MTD completed the task of dataset expansion more effectively. In case of insufficient number of samples (30% or 50% of the total data were used), the prediction accuracy of the models decreased as the amount of data decreased. Nevertheless, the HD–MTD technique effectively alleviates the decrease in prediction accuracy, because it uses HDs to describe the data.

Importantly, when trained on only 30% of the original data, our model with HD-MTD achieved an F1 score of 0.7931 (95% CI: [0.77, 0.81]), which is comparable to the performance of the model trained on 100% of the data without expansion (F1 = 0.8139). Statistical analysis confirmed that the difference between these two conditions is not significant (p > 0.05, paired bootstrap test), supporting the claim that our approach performs effectively even with substantially reduced training data. This finding highlights the practical utility of HD-MTD in scenarios where labeled data are scarce.

**Reviewer#3, Concern #9: The biological validation experiments focus only on a single predicted interaction between one miRNA and one target gene, which is insufficient to demonstrate the general predictive capability of the model across diverse miRNA target interactions.**

**Author response:** We thank the reviewer for this important observation regarding the scope of our experimental validation. We agree that validation of a single predicted interaction is insufficient to demonstrate the general predictive capability of the model across diverse miRNA–target pairs.

To address this concern, we have clarified throughout the manuscript that the current experimental validation serves as a proof-of-concept demonstration, illustrating that predictions generated by the model can be biologically validated, rather than providing a statistical assessment of overall predictive performance.

This clarification has been incorporated into the *Experimental validation* subsection and further emphasized in the Discussion, where we explicitly acknowledge this limitation and state that a single validated case does not provide an estimate of the model’s false positive rate or generalizability.

In addition, we have outlined future work involving large-scale experimental validation on a randomly selected cohort of predicted interactions (e.g., 20–30 targets) to more rigorously assess model performance across diverse biological contexts.

The corresponding revisions are as follows:

## Abstract

Predicting microRNA target genes is essential for understanding their biological functions. This study developed a miRNA target gene prediction model based on input-feature dependency. Features were treated as multiple random variables, with marginal densities estimated via Gaussian mixture models and dependencies captured by regular vine copula to derive joint probability density functions. We constructed class-conditional joint densities for positive and negative samples separately using GMM and R-vine copula, then combined these with prior probabilities via Bayes' rule to obtain posterior probabilities of positive interactions, using a 0.5 threshold for deterministic prediction. To address insufficient data and class imbalance, hybrid distribution mega-trend diffusion generated virtual samples. Computational validation showed high predictive performance even with only 30% of the original dataset. As proof of concept, we experimentally validated one predicted interaction (miR-8485 targeting JAK2) using dual-luciferase, cellular, and animal experiments, confirming biological relevance. These findings provide a valuable tool for understanding miRNA functions and disease mechanisms.

**Author summary**

In this study, we developed a new computational model to more accurately predict which genes are regulated by microRNAs—small RNA molecules that play key roles in health and disease. Predicting these targets is difficult because biological data are often limited, imbalanced, and contain complex relationships between features. Our model addresses these challenges by combining two innovations: a probabilistic prediction framework that accounts for dependencies between input features, and a data expansion method that generates realistic synthetic samples to balance the dataset. Computational experiments show that our model performs well even when trained on only 30% of the original data and outperforms existing methods in predictive accuracy. Through laboratory experiments, we validated one prediction—that miR-8485 targets the JAK2 gene—serving as a proof-of-concept demonstration of the model's biological relevance. Our findings provide researchers with a promising tool for uncovering microRNA functions, which can help advance our understanding of diseases and support the development of new therapies.

**Experimental validation: a proof-of-concept case study**

To demonstrate the biological relevance of predictions generated by our model, we selected one top-ranked prediction—miR-8485 targeting JAK2—for experimental validation. It is important to note that this single-case validation is intended as a proof-of-concept to illustrate that the model can generate biologically plausible hypotheses, rather than as a statistical validation of the model's overall predictive accuracy. The following dual-luciferase, cellular, and animal experiments were performed to test the biological validity of this specific predicted interaction.

## Discussion

This study has several limitations. First, experimental validation was performed on only one predicted interaction (miR-8485 targeting JAK2); while the dual-luciferase, cellular, and animal experiments confirmed the biological relevance of this specific prediction, a single positive case does not provide a statistical estimate of the model's false positive rate or its generalizability, and thus these results should be interpreted as a proof-of-concept demonstration of the model's utility for hypothesis generation rather than comprehensive validation of its predictive accuracy. Second, the training dataset (TarBase v8.0) is relatively small and imbalanced; although we employed HD-MTD to mitigate this issue, the synthetic samples may not fully capture the complexity of biological data.

Future work should address these limitations through multiple complementary directions. First, large-scale experimental validation on a randomly selected cohort of predictions (e.g., 20-30 interactions) is needed to rigorously assess the model's false positive rate. Second, incorporating additional training data from high-throughput technologies and integrating more diverse biological features—such as spatial structure data or miRNA–mRNA interaction kinetics—could further enhance prediction accuracy [30]. Third, exploring alternative synthetic sample generation strategies that better preserve biologically meaningful distributions would improve the handling of imbalanced data.
