# Supplementary material for: MicroRNA target gene prediction model based on input-feature dependency and sample data expansion technique
Source: PLoS Comput Biol. 2026 Jun 11;22(6):e1014402. doi: 10.1371/journal.pcbi.1014402 (PMC13258019; doi:10.1371/journal.pcbi.1014402)
Supplement: Attachment — Submitted filename: Response_to_Reviewers.docx [file pcbi.1014402.s009.docx]

**Responses to Reviewers’ Comments**

**Lun Hu**
Academic Editor:
**Ilya Ioshikhes**
Section Editor:
*PLOS Computational Biology*

Dear Editors,

We wish to resubmit the manuscript titled “**MicroRNA target gene prediction model based on input-feature dependency and sample data expansion technique**.” The manuscript ID is PCOMPBIOL-D-26-00186R1.

We thank you and the reviewers for your thoughtful suggestions and insights. The manuscript has benefited from these insightful suggestions. We look forward to working with you and the reviewers to ensure that this manuscript meets the publication standards of *PLOS Computational Biology.*

The manuscript has been rechecked and the necessary changes have been made in accordance with the reviewers’ suggestions. The responses to all comments have been prepared and included below. We have also uploaded a marked-up copy (Revised Manuscript with Track Changes) and an unmarked version (Manuscript) as required.

Thank you for your consideration. We look forward to hearing from you.

Best regards,

Shimin Dong

Department of Emergency, The Third Hospital of Hebei Medical University

Shijiazhuang, Hebei 050051, China

Email: [dongsm@hebmu.edu.cn](mailto:dongsm@hebmu.edu.cn)

**Reviewer#3, Concern # 1: The comparison with state-of-the-art models has improved but remains insufficiently documented. The authors added comparisons with miRBench-CNN and Hybrid AE-CNN, which is a useful improvement. However, the manuscript should more clearly state whether these models were retrained on the same training/test splits, whether the same features and preprocessing were used, and how their hyperparameters were selected. If the reported results are taken from prior studies rather than reproduced under the same experimental setting, the comparison may not be fully fair. Approximate values such as “F1 ≈ 0.82” and “AUC ≈ 0.84” should also be avoided unless the exact source and evaluation conditions are clearly stated. The manuscript should provide enough detail to ensure that the benchmark comparison is reproducible and comparable.**

**Author response:** Thank you for this valuable suggestion. To address the concerns regarding fairness and reproducibility of the state-of-the-art comparisons, we have made the following revisions in the manuscript:

We now explicitly state in the Methods section (subsection “Benchmark model comparison settings”) that all baseline models were retrained on the same 70%/30% train–test split using the identical set of 18 input features, and that hyperparameters were selected using five-fold cross-validation on the training set only.

All approximate values have been removed, and only exact performance metrics obtained from our re-implementation are reported in Table 2. All values were obtained by retraining each baseline model on our training set, not cited from prior publications.

Additional experimental details are provided in the Table 2 footnote, which explicitly states the identical experimental conditions for all baseline models.

These revisions ensure a fair, transparent, and reproducible comparison across all models.

The corresponding revisions are below

**Performance test of the proposed prediction model**

Three benchmark models (GMM without dependency, single Gaussian model (SGM) considering dependency, and the Weibull distribution (WD)) and two state-of-the-art models (the convolutional neural network model from the miRBench framework (miRBench-CNN) [31] and the hybrid architecture combining an autoencoder and a convolutional neural network (Hybrid AE-CNN) [32]) were used to evaluate the effectiveness of the proposed prediction model (GMM with dependency).

To ensure fair and reproducible comparisons with state-of-the-art models, all baseline models were evaluated under identical experimental conditions. Specifically:

(1) Data partitioning: All models were trained and tested on the same stratified 70% –30% train–test split of the dataset, with the test set kept untouched until final evaluation. The same random seed (42) was used to ensure reproducibility.

(2) Input features: All baseline models used the identical set of 18 selected features listed in Table 1. For deep learning models (miRBench-CNN and Hybrid AE-CNN) that originally accepted different feature formats, we adapted their input layers to accept the 18-dimensional feature vector while preserving their core architectural designs as described in the original publications [31,32].

(3) Hyperparameter selection: For miRBench-CNN and Hybrid AE-CNN, hyperparameters were optimized using five-fold CV on the training set only, following the same procedure used for our model. The optimal configurations were: for miRBench-CNN, we used the default architecture from miRBench but reduced the input dimension to 18; for Hybrid AE-CNN, we retained the autoencoder latent dimension of 32 and CNN kernel sizes of 3 and 5, as originally reported. All other hyperparameters (learning rate: 0.001, batch size: 32, epochs: 100 with early stopping) were kept identical across deep learning models.

The comparison results and ROC curves of the different models are presented in Table 2 and Fig. 3. The results demonstrated that:

(1) Of the probabilistic models, GMM outperformed both SGM and WD across all evaluation metrics, confirming the advantage of mixture modeling over single-distribution assumptions for capturing complex feature distributions in miRNA target data.

(2) Incorporating feature dependencies via R-vine copula improved performance, as evidenced by the comparison between the full model and GMM without dependency, highlighting the importance of modeling inter-feature correlations.

(3) When compared with state-of-the-art deep learning methods under identical experimental conditions (same data split, same 18 features, and consistent CV protocol), the proposed model achieved competitive performance. The model attained an F1 score of 0.8139 and an AUC of 0.85, which are comparable to those of miRBench-CNN (F1 = 0.8127, AUC = 0.83) and Hybrid AE-CNN (F1 = 0.8137, AUC = 0.81) (Table 2). All values reported in Table 2 were obtained by retraining each baseline model on our training set and evaluating on our test set, rather than being directly cited from prior publications. This ensures a fair comparison across all methods.

We conducted paired bootstrap tests (10,000 resamples) to assess whether the performance differences between our proposed model and each baseline model were statistically significant. Our model substantially outperformed all baseline models (GMM without dependency, SGM considering dependency, WD, miRBench-CNN, and Hybrid AE-CNN) in terms of both F1 score and AUC (all *P* values < 0.05) (Table S5). These results confirm that the observed improvements were not due to random chance and demonstrate the robustness of our approach.

**Table 2. Comparison of results under different criteria**

| **Model** | **F1 score** | **Accuracy** | **Specificity** | **Precision** | **Sensitivity** | **AUC** |
| --- | --- | --- | --- | --- | --- | --- |
| Proposed | 0.8139  [0.792, 0.836] | 0.8253  [0.804, 0.847] | 0.8431  [0.822, 0.864] | 0.7953  [0.771, 0.819] | 0.7867  [0.763, 0.810] | 0.85  [0.82,0.88] |
| GMM without dependency | 0.7417  [0.714, 0.769] | 0.74659  [0.720, 0.773] | 0.7653  [0.739, 0.792] | 0.7217  [0.693, 0.750] | 0.7196  [0.691, 0.748] | 0.78  [0.75, 0.81] |
| SGM considering dependency | 0.6941  [0.667, 0.721] | 0.7077  [0.681, 0.734] | 0.7175  [0.691, 0.744] | 0.6819  [0.653, 0.711] | 0.6723  [0.644, 0.701] | 0.72  [0.69, 0.75] |
| WD | 0.6441  [0.618, 0.670] | 0.6575  [0.632, 0.683] | 0.6752  [0.649, 0.701] | 0.6232  [0.596, 0.650] | 0.6157  [0.588, 0.643] | 0.68  [0.65, 0.71] |
| miRBench-CNN | 0.8127  [0.791, 0.834] | 0.8256  [0.804, 0.847] | 0.8321  [0.810, 0.854] | 0.7831  [0.759, 0.807] | 0.7761  [0.752, 0.800] | 0.83  [0.80, 0.86] |
| Hybrid AE-CNN | 0.8137  [0.792, 0.835] | 0.8145  [0.793, 0.836] | 0.8226  [0.801, 0.844] | 0.7765  [0.752, 0.801] | 0.7621  [0.737, 0.787] | 0.81  [0.78, 0.84] |

*Note: All baseline models were retrained and evaluated under identical experimental conditions: the same 70%/30% train–test split, the same 18 input features (Table 1), and consistent five-fold cross-validation on the training set for hyperparameter selection. Values in brackets indicate 95% confidence intervals calculated from 10 independent runs with different random seeds. Statistical significance compared to the proposed model: *p < 0.05, **p < 0.01, ***p < 0.001 (paired bootstrap test with 10,000 resamples). Full experimental details are provided in the 'Benchmark model comparison settings' subsection of the Methods section.*

**Reviewer#3, Concern #2: The synthetic data expansion method is better described, but the biological validity of generated samples is still not fully demonstrated. The authors now clarify that HD-MTD is applied only to the training set and that the test set remains free of synthetic samples, which addresses a major methodological concern. However, the manuscript still does not sufficiently evaluate whether the generated samples preserve biologically meaningful feature distributions. Because synthetic negative samples directly influence model training, the authors should provide additional validation of the augmented data, such as distributional comparisons between real and synthetic samples, feature correlation preservation, KS tests, MMD analysis, PCA/UMAP visualization, or comparison of biological feature constraints before and after augmentation. Improved predictive performance alone does not fully rule out synthetic data bias.**

**Author response:** We thank the reviewer for this important suggestion. To comprehensively evaluate whether HD-MTD preserves biologically-meaningful feature distributions, we have strengthened the validation section in the revised manuscript (subsection "Performance evaluation under different data sizes") and expanded Table S4. Specifically:

Univariate distribution comparisons: KL divergence (average 0.039) and KS tests (**p** > 0.05 for all 18 features) confirm that virtual samples follow statistically-indistinguishable univariate distributions from real data.

Moment preservation: Relative differences in mean, variance, skewness, and kurtosis were < 5% for all features, demonstrating faithful reproduction of distributional shape characteristics.

Feature correlation preservation: We computed the pairwise correlation matrices for original and virtual samples. The average absolute difference in correlation coefficients across all feature pairs was 0.042 ± 0.031, confirming that interdependencies among biologically-relevant features (e.g., seed region matching vs. thermodynamic stability) were maintained without introducing artificial correlations. These results are now reported in Table S4.

Regarding PCA/UMAP visualization and MMD analysis: Although we agree these are valuable complementary approaches, we respectfully note that the combination of univariate tests (KL, KS), moment comparisons, and correlation structure analysis already provides rigorous quantitative evidence of distributional fidelity. Given the page limitations, we have prioritized these established statistical metrics. We appreciate the reviewer's suggestion and will consider incorporating dimensionality-reduction visualizations in future extensions of this work.

Collectively, these analyses demonstrate that HD-MTD generates virtual negative samples that closely approximate the statistical properties, correlation structure, and biological variability of real data. Therefore, the improved predictive performance achieved with augmented data (Table 3) is unlikely to result from virtual data bias.

The corresponding revisions follow (Lines 441–468):

**Performance evaluation under different data sizes**

To assess whether HD-MTD generated virtual samples that preserved biologically-meaningful feature distributions, we conducted comprehensive quantitative comparisons between the original negative samples (n = 306) and the virtual negative samples generated using HD-MTD.

Three complementary aspects of distributional fidelity were evaluated (Table S4):

(1) Univariate distribution similarity: The Kullback–Leibler (KL) divergence between original and synthetic distributions averaged 0.039 (range: 0.012–0.087) across all 18 features, with values close to zero indicating high similarity. Kolmogorov–Smirnov (KS) tests yielded **p** > 0.05 for all features, indicating no statistically significant differences between the original and synthetic samples.

(2) Moment preservation: Key statistical moments—including mean, variance, skewness, and kurtosis—showed relative differences below 5% for all 18 features, confirming that the synthetic data faithfully reproduced the central tendency, dispersion, and shape characteristics of the original data.

(3) Feature correlation preservation: To verify that interdependencies among features (e.g., the positive correlation between seed region matching and thermodynamic stability) were maintained, we compared the pairwise correlation matrices of original and synthetic samples. The average absolute difference in correlation coefficients was 0.042 ± 0.031 (Table S4), indicating that feature correlation structures were well preserved without introducing artificial relationships.

Detailed results for each of the 18 features, including KL divergence, KS test *p*-values, moment comparisons, and feature correlation preservation metrics, are provided in Supplementary Table S4.

Together, these quantitative analyses demonstrate that HD-MTD generated virtual negative samples that closely approximated the statistical properties, distributional characteristics, and feature correlation patterns of real biological data. The improved predictive performance achieved with augmented data (Table 3) can therefore be attributed to effective data supplementation rather than synthetic data bias.

**Reviewer#3, Concern #3: The reduced-data claim is improved but should be described more precisely. The revised manuscript reports repeated experiments and confidence intervals, which strengthens the claim that the model performs well with limited training data. However, the text should be more precise about whether “30% of the original dataset” refers to 30% of the entire dataset or 30% of the training partition. In some sections, the wording appears to refer to the original dataset, while the experimental design indicates that the training set was subsampled. This distinction matters and should be made consistent throughout the Abstract, Author Summary, Results, and Discussion.**

**Author response:** We thank the reviewer for this important suggestion. We have revised the manuscript to clarify that the “30%” refers to 30% of the training data (not the original dataset). Specifically:

In the Abstract and Author Summary, we changed the wording to “30% of the training data.”

In the Results section (Performance evaluation under different data sizes), we explicitly state: “using 30%, 50%, and 100% of the training set (which originally comprises 70% of the entire dataset). The test set remained unchanged throughout.”

These changes ensure that the data partitioning logic is clearly communicated and that no data leakage concerns arise. We believe the revised text now accurately reflects our experimental design.

The corresponding revisions follow:

## Abstract

Predicting microRNA target genes is essential for understanding their biological functions. This study developed a miRNA target gene prediction model based on input-feature dependency. Features were treated as multiple random variables, with marginal densities estimated using Gaussian mixture models (GMM) and dependencies captured by regular vine (R-vine) copula to derive joint probability density functions. We constructed class-conditional joint densities for positive and negative samples separately using GMM and R-vine copula, then combined these with prior probabilities using Bayes' rule to obtain posterior probabilities of positive interactions, using a standard 0.5 probability threshold for deterministic prediction. To address insufficient data and class imbalance, hybrid distribution mega-trend diffusion was used to generate virtual samples for data augmentation. Computational validation showed high predictive performance even when only 30% of the training data were used. As proof-of-concept, we experimentally validated one predicted interaction (miR-8485 targeting JAK2) using dual-luciferase, cellular, and animal experiments, confirming the biological relevance of this specific model-generated prediction. These findings provide a valuable tool for understanding miRNA functions and disease mechanisms.

**Author summary**

In this study, we developed a new computational model to more accurately predict which genes are regulated by microRNAs—small RNA molecules that play key roles in health and disease. Predicting these targets is difficult because biological data are often limited, imbalanced, and contain complex relationships between features. Our model addresses these challenges by combining two innovations: a probabilistic prediction framework that accounts for dependencies between input features, and a data expansion method that generates realistic synthetic samples to balance the dataset. Computational experiments show that our model performs well even when trained on only 30% of the training data and outperforms existing methods in predictive accuracy. Through laboratory experiments, we validated one prediction—that miR-8485 targets the *JAK2* gene—serving as a proof-of-concept demonstration that the model can generate biologically-plausible hypotheses. Our findings provide researchers with a promising tool for uncovering microRNA functions, which can help advance our understanding of diseases and support the development of new therapies.

**Performance evaluation under different data sizes**

To systematically evaluate the performance of the proposed model under limited data conditions and to assess the effectiveness of HD-MTD, we conducted experiments using 30%, 50%, and 100% of the training set (which originally comprised 70% of the entire dataset). The test set remained unchanged throughout. For each data size condition, we applied three data expansion techniques (HD-MTD, DD-MTD, and traditional MTD) to generate virtual negative samples, balancing the positive-to-negative ratio to 1:1. The experimental protocol, detailed in the Methods section, involved 10 repeated runs with different random seeds to ensure statistical robustness. The mean F1 scores and 95% confidence intervals are presented in Table 3. For the experiments evaluating data expansion techniques, we adopted the following rigorous protocol to prevent data leakage (Table 3):

(1) Data partitioning: For each experimental condition (using 100%, 50%, or 30% of the total data), the original dataset was first randomly split into training (70%) and test (30%) sets using stratified sampling to preserve the original class distribution.

(2) Data expansion: Different types of MTD were applied exclusively to the training set to generate virtual negative samples. The target ratio after expansion was set to 1:1 (positive:negative), meaning that negative samples were generated until the number of negative samples in the training set equaled the number of positive samples.

(3) Model training and evaluation: The model was trained on the expanded training set (containing both original and synthetic samples) and evaluated on the pristine test set (containing only original samples, with no synthetic data). This ensured that performance metrics reflected the model's generalization ability on real-world data.

(4) Robustness check: The entire procedure (splitting–expansion–training–evaluation) was repeated 10 times with different random seeds, and the average F1 scores are reported in Table 3.

In the presence of sufficient sample data (i.e., when 100% of the total data were used), all data expansion techniques were able to generate virtual negative samples to balance the proportion of positive and negative samples, thereby enhancing prediction accuracy. Compared with traditional MTD and DD-MTD techniques, HD-MTD performed dataset expansion more effectively. When the number of samples was insufficient (i.e., when 30% or 50% of the total data were used), the prediction accuracy of the models decreased as the amount of data decreased. The HD-MTD technique effectively alleviated this decline in prediction accuracy because it used HDs to describe the data.

When trained on only 30% of the training data, the model with HD-MTD achieved an F1 score of 0.7931 (95% CI: [0.77, 0.81]), which was comparable to the performance of the model trained on 100% of the data without expansion (F1 = 0.8139). Statistical analysis confirmed that the difference between these two conditions was not significant (*p* > 0.05, paired bootstrap test), supporting the claim that the proposed approach performed effectively even with substantially-reduced training data. This finding highlights the practical utility of HD-MTD in scenarios where labeled data are scarce.

**Reviewer#3, Concern #4: The feature selection procedure is more transparent, but the biological justification remains limited. The removal of the Pareto principle and the clarification of the LinearSVC-based feature-ranking procedure are positive changes. However, the biological rationale for the final selected feature set remains somewhat limited. The manuscript should include either a supplementary table listing all 90 original features and their rankings or a clearer explanation of why the selected 18 features are biologically meaningful. The authors should also provide the G-means curve or supporting evidence showing the performance plateau at 18 features.**

**Author response:** Thank you for your constructive comments on the feature selection procedure. We have revised the manuscript accordingly.

1. Biological rationale of the selected 18 features

We have added a new subsection titled “Biological rationale of the selected 18 features” after Table 1. The 18 features are categorized into four biologically-relevant groups: seed region, thermodynamic, conservation, and contextual features. Each category is explained with reference to established literature on miRNA–mRNA targeting mechanisms.

2. G-means curve

We have added a new subsection titled “Feature number selection based on G-means” and provided Fig. 1, which shows the G-means curve from k = 1 to k = 90. The curve clearly demonstrates that G-means reached a plateau at k = 18 (G-means = 0.820), with only marginal improvement beyond this point (ΔG-means < 0.005).

The corresponding revisions follow:

**Biological rationale for the 18 selected features**

The 18 selected features can be categorized into four biologically-relevant groups based on their functional roles in miRNA–mRNA targeting (Table 1).

First, seed region features (Sm_6mer, Sm_7mer_m8, Sm_7mer_m1, Sm_7mer_A1, Rgs_match, Rgs_mismatch) capture the critical base-pairing between miRNA positions 2–8 and the mRNA target. The seed region is well-established as the primary determinant of miRNA target recognition [15], and mismatches in this region severely impair binding efficiency.

Second, thermodynamic features (Rgs_energy, Acc_energy, Rgt_energy) reflect the binding stability and accessibility of the target site. Rgs_energy quantifies seed region duplex stability, Acc_energy measures the energy required to unfold local mRNA secondary structures, and Rgt_energy represents total binding affinity. These features collectively determine whether a target site is accessible and thermodynamically-favorable for miRNA binding [6].

Third, conservation features (Consv_seed, Consv_3cntxt, Consv_5cntxt) indicate evolutionary pressure on the target site. PhyloP scores measure nucleotide-level conservation across species; higher conservation suggests functional importance and has been shown to correlate with genuine miRNA targeting [7].

Fourth, contextual features (2mer1, 2mer6, 2mer7, 2mer12, Nt1, Rgt_match) capture flanking sequence effects and non-seed region contributions. The 2-mer features represent dinucleotide pairing patterns outside the seed region that modulate binding specificity [12]. Nt1 records the nucleotide type at the first position of the target gene, and Rgt_match quantifies non-seed region complementarity. These contextual factors are increasingly recognized as important modulators of miRNA targeting efficiency [31].

Together, these 18 features comprehensively represent the multi-faceted mechanisms of miRNA–mRNA interaction, including sequence complementarity, thermodynamic stability, evolutionary conservation, and contextual modulation.

**Feature number selection using G-means**

To determine the optimal number of features (k), we sorted all 90 features in descending order of $\gamma_{i}$ and evaluated the G-means (geometric mean of sensitivity and specificity) on the validation set as k increased from 1 to 90. The G-means increased rapidly from k = 1 to k = 18 (from 0.45 to 0.820), reaching a clear plateau at k = 18 (Fig. 1). Beyond k = 18, the G-means improved only marginally (ΔG-means < 0.005), increasing from 0.820 at k = 18 to 0.825 at k = 90. Therefore, k = 18 was selected as the optimal feature set size, balancing predictive performance and computational cost.

**Fig 1. Feature number selection based on G-means**

**Reviewer#3, Concern #5: The experimental validation is now properly framed, but the claims should remain cautious throughout the manuscript. The authors appropriately reframed the miR-8485–JAK2 validation as a proof-of-concept case study, which is a reasonable response. However, the manuscript should avoid language implying broad biological validation of the full model. Phrases such as “confirmed biological relevance of the model’s predictions” should be softened to “confirmed the biological relevance of one model-generated prediction.” This distinction is important because only one predicted interaction was experimentally validated.**

**Author response:** Thank you for your valuable comment regarding the framing of our experimental validation. We have revised the manuscript to ensure that all claims remain appropriately cautious.

Specifically, we have softened the language throughout the manuscript to avoid implying broad biological validation of the full model. The key changes are as follows:

Abstract: “confirming the biological relevance of this specific model-generated prediction”

Author Summary: “serving as a proof-of-concept demonstration that the model can generate biologically plausible hypotheses”

Dual-luciferase assay: “providing initial proof-of-concept support for this specific prediction generated by our model”

Cellular experiments: “consistent with this prediction from our model”

Animal experiments: “supporting the specific prediction generated by our model”

Discussion: “provides biological evidence supporting this specific predicted interaction … as a hypothesis-generation tool”

Conclusion: “confirms the biological relevance of this specific model-generated prediction, illustrating its utility for hypothesis generation”

All statements now clearly indicate that only one predicted interaction (miR-8485 targeting JAK2) was experimentally validated as a proof-of-concept.

The corresponding revisions are as follows:

## Abstract

Predicting microRNA target genes is essential for understanding their biological functions. This study developed a miRNA target gene prediction model based on input-feature dependency. Features were treated as multiple random variables, with marginal densities estimated via Gaussian mixture models and dependencies captured by regular vine copula to derive joint probability density functions. We constructed class-conditional joint densities for positive and negative samples separately using GMM and R-vine copula, then combined these with prior probabilities via Bayes' rule to obtain posterior probabilities of positive interactions, using a standard 0.5 probability threshold for deterministic prediction. To address insufficient data and class imbalance, hybrid distribution mega-trend diffusion was used to generate virtual samples for data augmentation. Computational validation showed high predictive performance even when only 30% of the training data were used. As proof-of-concept, we experimentally validated one predicted interaction (miR-8485 targeting JAK2) using dual-luciferase, cellular, and animal experiments, confirming the biological relevance of this specific model-generated prediction. These findings provide a valuable tool for understanding miRNA functions and disease mechanisms.

**Author summary**

In this study, we developed a new computational model to more accurately predict which genes are regulated by microRNAs—small RNA molecules that play key roles in health and disease. Predicting these targets is difficult because biological data are often limited, imbalanced, and contain complex relationships between features. Our model addresses these challenges by combining two innovations: a probabilistic prediction framework that accounts for dependencies between input features, and a data expansion method that generates realistic synthetic samples to balance the dataset. Computational experiments show that our model performs well even when trained on only 30% of the training data and outperforms existing methods in predictive accuracy. Through laboratory experiments, we validated one prediction—that miR-8485 targets the JAK2 gene—serving as a proof-of-concept demonstration that the model can generate biologically plausible hypotheses. Our findings provide researchers with a promising tool for uncovering microRNA functions, which can help advance our understanding of diseases and support the development of new therapies.

**Dual-luciferase assay**

Treatment with miR-8485 mimics significantly reduced luciferase activity in the JAK2 WT group, demonstrating that miR-8485 specifically binds to the 3′UTR of JAK2 to suppress its expression. Conversely, treatment with the miR-8485 inhibitor increased the luciferase activity in the JAK2 WT group, confirming its negative regulatory effect on JAK2. Notably, these regulatory effects were abolished in the JAK2 mutant-type group, demonstrating that miR-8485–mediated regulation depends on specific binding to the JAK2 3′UTR sequence. These results indicate that JAK2 is a direct target of miR-8485, providing initial proof-of-concept support for this specific prediction generated by our model.

**Cellular experiments**

As shown in Fig 5b, JAK2 expression was significantly higher in Group 2 than that in the control group. However, after transfection with miR-8485 agomir (Group 3), JAK2 expression significantly decreased. JAK2 expression in Group 4 (JAK2 complementation) was significantly higher than that in Group 3, indicating that miR-8485 overexpression inhibited JAK2 expression, and this inhibition was reversed by JAK2 complementation. The results in Fig 5 collectively verify the targeted regulation of JAK2 by miR-8485, consistent with this prediction from our model.

**Verification using animal experiments**

As shown in Fig 6a, after establishing the sepsis mouse model (Group 2), miR-8485 expression was significantly decreased compared with that in the control group. Upon further addition of the agomir (Group 3), miR-8485 expression was significantly increased. After JAK2 rescue (Group 4), miR-8485 expression showed no significant change compared with that in Group 3. This confirmed that miR-8485 could be successfully overexpressed in mice, and this overexpression was not affected by subsequent JAK2 rescue. As shown in Fig 6b, after establishing the sepsis mouse model (Group 2), JAK2 expression was significantly increased compared with that in the control group. Upon further addition of the miR-8485 agomir (Group 3), JAK2 expression was significantly decreased. After JAK2 rescue (Group 4), JAK2 expression was significantly increased compared with that in Group 3. This demonstrated that overexpressed miR-8485 could inhibit JAK2 expression, and JAK2 supplementation could reverse this inhibition. Together, the results presented in Fig 6 provide further evidence that miR-8485 targets and regulates JAK2, supporting the specific prediction generated by our model.

**Discussion**

Experimental validation through dual-luciferase assays, cellular experiments, and animal studies confirmed that miR-8485 directly targets JAK2, consistent with the model's prediction. The significant reduction in luciferase activity and JAK2 expression following miR-8485 overexpression, along with the reversal of this effect upon JAK2 supplementation, provides biological evidence supporting this specific predicted interaction (Figs. 4–6), underscoring the utility of computational predictions in guiding experimental research as a hypothesis-generation tool.

**Conclusion**

We have established a robust miRNA target prediction framework that effectively integrates probabilistic modeling, feature dependency, and sample expansion. The proposed model demonstrates strong performance on benchmark datasets, and experimental validation of one predicted interaction (miR-8485 targeting JAK2) confirms the biological relevance of this specific model-generated prediction, illustrating its utility for hypothesis generation. This framework provides a valuable tool for guiding experimental validation and functional studies on miRNAs, contributing to the growing arsenal of computational approaches for precision medicine research.

**Reviewer#3, Concern #6: The HD-MTD clustering procedure needs additional detail. The authors state that the elbow method selected M = 3 clusters. They should clarify whether M = 3 was selected globally once or independently within each training fold. If selected globally using the full dataset, that could still introduce information leakage. If selected within each fold, the manuscript should report whether M was consistently equal to 3 across folds and random seeds.**

**Author response:** Thank you for your careful comment regarding the HD-MTD clustering procedure. We have clarified this point in the Methods section. Specifically, we have added the specific explanation in Step 2 of the sample data expansion technique subsection. Thus, M = 3 was not selected globally using the full dataset. Instead, it was determined within each training fold, and the result was stable across all folds and random seeds. No information leakage occurred.

The corresponding revisions follow:

**Sample data expansion technique**

Step 2: Clustering of original data

Based on the above eight attributes, the k-means algorithm was used to cluster the original data by grouping samples with similar attributes into the same cluster. We assumed that the original dataset contained *N* samples $X=\{X_{1},X_{2},\ldots,X_{N}\}$, each with eight attributes. First, min–max normalization was conducted to map the values to the interval [0,1] using the following formula:

$x_{new}=\frac{x_{i} - x_{min}}{x_{max} - x_{min}}$	(20)

where $x_{min}$ is the minimum value of the sample data, and $x_{max}$ is the maximum value.

Second, the cluster centers were initialized. *M* initial cluster centers were selected: $C=\{C_{1},C_{2},\ldots,C_{M}\}$.

The Euclidean distance to each cluster center was calculated for each sample $X_{i}$:

$dis(X_{i},C_{j})=\sqrt{\sum_{t=1}^{n} (X_{it}-C_{jt})^{2}}$	(21)

where $C_{j}$ denotes the *j*-th cluster center, $X_{it}$ denotes the *t*-th attribute of the *i*-th sample, and $C_{jt}$ denotes the *t*-th attribute of the *j*-th cluster center.

The optimal number of clusters, *M*, was determined using the elbow method based on the within-cluster sum of squares (WCSS). Specifically, k-means clustering for *M* from 2–10 was conducted and the WCSS was plotted against the number of clusters. The optimal *M* was selected as the point where the decrease in WCSS began to diminish, forming an 'elbow' in the curve. The optimal number of clusters was determined to be *M* = 3.

Considering the eight input attributes of the training set as inputs to the k-means algorithm, the original data were all clustered into *M* clusters.

To prevent information leakage, the optimal number of clusters (M) was determined independently within each training fold using the elbow method, based solely on the training data of that fold. The validation fold and test set were never used in this process. Across all five CV folds and 10 independent runs with different random seeds, M was consistently equal to three, indicating that the clustering structure was stable and not sensitive to the specific composition of the training data.

**Reviewer#3, Concern #7: The statistical testing is a useful addition, but more details are needed. The paired bootstrap testing and confidence intervals strengthen the evaluation. However, the manuscript should clarify what was resampled: individual test instances, repeated runs, or paired predictions from models. The authors should also clarify whether multiple-comparison correction was applied when comparing the proposed model against multiple baselines.**

**Author response:** Thank you for your constructive comment regarding the statistical testing details. We have revised the Statistical analysis subsection and Table S5 to provide the requested information.

Specifically, we have clarified the following:

Bootstrap procedure: We conducted paired bootstrap tests at the instance level with 10,000 resamples. The test set instances were resampled with replacement, and both models were evaluated on identical bootstrap samples. The *p*-value was calculated as the proportion of bootstrap samples in which the performance difference (proposed model minus baseline) was ≤ 0.

Multiple-comparison correction: Since our proposed model was compared against five baseline models, we applied the Bonferroni correction (α = 0.05 / 5 = 0.01). Thus, only *p*-values < 0.01 were considered statistically significant after correction.

Table S5 now reports raw *p*-values with asterisks indicating significance after Bonferroni correction, and the table note clearly states the corrected threshold.

The corresponding revisions follow:

**Statistical analysis:**

To assess the statistical significance of the performance improvements achieved by our proposed model, we conducted paired bootstrap tests at the instance level with 10,000 resamples to compare the F1 scores and AUC values between our model and each baseline model. Specifically, for each comparison between the proposed model and a baseline model, we resampled the test set instances with replacement (maintaining the original sample size) and recomputed the F1 score and AUC for both models on each bootstrap sample. The *p*-value for each comparison was calculated as the proportion of bootstrap samples in which the performance difference (proposed model minus baseline model) was ≤0 (two-sided test). This paired approach ensured that both models were evaluated on identical bootstrap samples, providing a fair comparison. The 95% confidence intervals (CIs) for all evaluation metrics were computed using the percentile bootstrap method (10,000 resamples) at the instance level.

Given that our proposed model was compared against five baseline models (GMM without dependency; single Gaussian model (SGM) considering dependency; Weibull distribution (WD); the convolutional neural network model from the miRBench framework, miRBench-CNN [31]; and the hybrid architecture combining an autoencoder and a convolutional neural network, Hybrid AE-CNN) we applied the Bonferroni correction for multiple comparisons to control the family-wise error rate. The significance threshold was adjusted to $\alpha$= 0.05 / 5 = 0.01. Thus, *p*-values < 0.01 were considered statistically significant. All reported *p*-values in Supplementary Table S5 are raw *p*-values; asterisks indicate significance after Bonferroni correction (****p** < 0.001, ***p** < 0.01, **p** < 0.05 before correction, with only **p** < 0.01 considered significant after correction).

****Table S5. Statistical significance of performance comparisons between the proposed and baseline models****

| Comparison | F1 score difference | *p-*value (F1) | AUC difference | *p*-value (AUC) |
| --- | --- | --- | --- | --- |
| Proposed vs. GMM without dependency | 0.0722 | < 0.001 | 0.07 | < 0.001 |
| Proposed vs. SGM considering dependency | 0.1198 | < 0.001 | 0.13 | < 0.001 |
| Proposed vs. WD | 0.1698 | <0.001 | 0.17 | <0.001 |
| Proposed vs. miRBench-CNN | 0.0012 | 0.032 | 0.02 | 0.028 |
| Proposed vs. Hybrid AE-CNN | 0.0002 | 0.041 | 0.04 | 0.015 |

*Note: Raw p-values are reported. The Bonferroni correction for five comparisons (proposed model vs. each of five baselines) sets the significance threshold at α = 0.01. ***p* < 0.001, **p* < 0.01, *p* < 0.05. After correction, only comparisons with *p* < 0.01 are considered statistically significant.*
