# Supplementary material for: MicroRNA target gene prediction model based on input-feature dependency and sample data expansion technique
Source: PLoS Comput Biol. 2026 Jun 11;22(6):e1014402. doi: 10.1371/journal.pcbi.1014402 (PMC13258019; doi:10.1371/journal.pcbi.1014402)
Supplement: Peer review history

# Peer review: Decision Letter 0

**DOI:** 10.1371/journal.pcbi.1014402.r001

**Authors:** Ilya Ioshikhes, Lun Hu

10 Mar 2026

PCOMPBIOL-D-26-00186

MicroRNA target-gene prediction model based on input-feature dependency and sample data expansion technique

PLOS Computational Biology

Dear Dr. Dong,

Thank you for submitting your manuscript to PLOS Computational Biology. After careful consideration, we feel that it has merit but does not fully meet PLOS Computational Biology's publication criteria as it currently stands. Therefore, we invite you to submit a revised version of the manuscript that addresses the points raised during the review process.

Please submit your revised manuscript by May 09 2026 11:59PM. If you will need more time than this to complete your revisions, please reply to this message or contact the journal office at ploscompbiol@plos.org. When you're ready to submit your revision, log on to https://www.editorialmanager.com/pcompbiol/ and select the 'Submissions Needing Revision' folder to locate your manuscript file.

Please include the following items when submitting your revised manuscript:

* A letter that responds to each point raised by the editor and reviewer(s). You should upload this letter as a separate file labeled 'Response to Reviewers'. This file does not need to include responses to formatting updates and technical items listed in the 'Journal Requirements' section below.

* A marked-up copy of your manuscript that highlights changes made to the original version. You should upload this as a separate file labeled 'Revised Manuscript with Track Changes'.

* An unmarked version of your revised paper without tracked changes. You should upload this as a separate file labeled 'Manuscript'.

If you would like to make changes to your financial disclosure, competing interests statement, or data availability statement, please make these updates within the submission form at the time of resubmission. Guidelines for resubmitting your figure files are available below the reviewer comments at the end of this letter

We look forward to receiving your revised manuscript.

Kind regards,

Lun Hu

Academic Editor

PLOS Computational Biology

Ilya Ioshikhes

Section Editor

PLOS Computational Biology

**Additional Editor Comments:**

I received three review reports for our invited reviewers, and they all found merit in this work. However, they also raised several critical concerns regarding the quality of this work, such as inconsistent writing, the clarification of experiment setup, extension of performance validations, and insufficient methodology details. Due to these issues, I would like ask authors to carefully revise their manuscript for a major revision.

**Journal Requirements:**

1) Please ensure that the CRediT author contributions listed for every co-author are completed accurately and in full.

At this stage, the following Authors/Authors require contributions: Yan Shao, Yazhou Li, Hexin Zhai, and Shimin Dong. Please ensure that the full contributions of each author are acknowledged in the "Add/Edit/Remove Authors" section of our submission form.

The list of CRediT author contributions may be found here: https://journals.plos.org/ploscompbiol/s/authorship#loc-author-contributions

2) Please upload all main figures as separate Figure files in .tif or .eps format. For more information about how to convert and format your figure files please see our guidelines:

https://journals.plos.org/ploscompbiol/s/figures

3) Some material included in your submission may be copyrighted. According to PLOS’s copyright policy, authors who use figures or other material (e.g., graphics, clipart, maps) from another author or copyright holder must demonstrate or obtain permission to publish this material under the Creative Commons Attribution 4.0 International (CC BY 4.0) License used by PLOS journals. Please closely review the details of PLOS’s copyright requirements here: PLOS Licenses and Copyright. If you need to request permissions from a copyright holder, you may use PLOS's Copyright Content Permission form.

Please respond directly to this email and provide any known details concerning your material's license terms and permissions required for reuse, even if you have not yet obtained copyright permissions or are unsure of your material's copyright compatibility. Once you have responded and addressed all other outstanding technical requirements, you may resubmit your manuscript within Editorial Manager.

Potential Copyright Issues:

i) Exfect 2000 Transfection Reagent folder in the supplementary material includes clipart. Please confirm whether you drew the images / clip-art within the figure panels by hand. If you did not draw the images, please provide (a) a link to the source of the images or icons and their license / terms of use; or (b) written permission from the copyright holder to publish the images or icons under our CC BY 4.0 license. Alternatively, you may replace the images with open source alternatives. See these open source resources you may use to replace images / clip-art:

- https://commons.wikimedia.org

- https://openclipart.org/

ii) Exfect 2000 Transfection Reagent folder, In-Vivo Plasmid DNA Mid-Quantity Kit and DL101-Dual Luciferase Reporter Assay Kit1534845724946 in the supplementary material contain logos. We are not permitted to publish this under our CC BY 4.0 license, even with permission. We ask that you please remove or replace it.

4) Please ensure that the funders and grant numbers match between the Financial Disclosure field and the Funding Information tab in your submission form. Note that the funders must be provided in the same order in both places as well. Currently, the order of the grants is different in both places.

Note: If the reviewer comments include a recommendation to cite specific previously published works, please review and evaluate these publications to determine whether they are relevant and should be cited. There is no requirement to cite these works unless the editor has indicated otherwise.

**Reviewers' comments:**

Reviewer's Responses to Questions

Reviewer #1: 1. The authors must immediately clarify a critical ambiguity in the methodological framework that fundamentally undermines the reproducibility of this work. Specifically, the transition from the 90 initially extracted features to the final 18-feature set, selected via the G-means criterion, lacks a transparent reporting of the feature selection process itself. While equation 19 defines a feature effectiveness measure γᵢ, the manuscript fails to disclose the algorithm or procedure used to solve the convex quadratic programming problem that yields the optimal weight vector ω*. The reader is left to assume a standard support vector machine implementation, but this must be explicitly stated, including the specific solver, kernel function (if any, despite the linear decision function implied), and the method for tuning the penalty parameter C. Without this information, the derived γᵢ values and the subsequent ranking of features cannot be verified or replicated by the community, rendering the core of the model's interpretability claims unsubstantiated. The authors must provide a complete algorithmic description, including all hyperparameters, in a revised methods section.

2. The justification for using the Pareto principle to reduce the feature set from 90 to 18 is presented as a fait accompli and is scientifically unsound in this context. The authors state that "80% of the effects are determined by the most important 20% of the characteristics," but this empirical rule from economics and quality control is not a biological law and cannot be used to arbitrarily truncate a feature set. The decision to fix k=18 based on the observation that G-means plateaus after this point is a valid empirical approach, but referencing the Pareto principle adds no scientific value and introduces a logical fallacy. The authors must remove this spurious justification and instead frame the feature selection purely as an optimization based on the G-means criterion, which is already a sufficient and valid method. The current wording detracts from the otherwise sound methodological approach.

3. The derivation and presentation of the joint probability density function in equation 7 are mathematically incomplete and potentially incorrect, requiring an immediate revision. The equation presents the joint density f(T₁,...,T_d) as a product of marginal densities f_d(T_d) and a product of bivariate copula densities. However, the notation f_d(T_d) is poorly defined; it should clearly indicate that it is the product of all marginal densities, i.e., ∏ f_i(T_i). More critically, the indices in the second product term are ambiguous. The notation ∏_{k∈E_i} P_{y(k),z(k)|M(k)} is technically correct for a vine copula, but the authors have failed to define the arguments of these copula density functions in the equation itself. A copula density takes as inputs the conditional distribution functions, e.g., c(F(T_y|T_M), F(T_z|T_M)). While these are defined in equation 8 for a specific case, they must be included as arguments in the main joint density formula to be mathematically rigorous. The authors must rewrite equation 7 to include the proper arguments for the copula densities, ensuring the equation is a complete and accurate representation of an R-vine density.

4. The experimental validation, while commendable for including in-vitro and in-vivo work, is fundamentally confounded by a selection bias that must be addressed. The authors validated a single prediction—miR-8485 targeting JAK2. Selecting only one successful validation case constitutes "cherry-picking" and provides no information about the model's false positive rate or its performance across the diverse set of its predictions. The dual-luciferase, cellular, and animal experiments are powerful demonstrations of the biological relevance of one predicted interaction, but they do not constitute a validation of the model's overall predictive accuracy. To substantiate their claim of high accuracy, the authors must either provide a statistical summary of validation experiments on a randomly selected, representative cohort of predictions (e.g., 20-30 predicted interactions) or, at a minimum, report on the outcome of any negative or failed validations they may have performed. Presenting a single positive case is anecdotal evidence, not model validation.

5. The description of the Hybrid Distribution Mega-Trend Diffusion (HD-MTD) technique is critically under-specified in the manuscript, preventing any meaningful evaluation or adoption of the method. Step 2 involves clustering the original data using K-means based on eight statistical attributes. The authors must specify the value of M (the number of clusters) and, crucially, justify how this number was determined. Was it based on an elbow method, a silhouette score, or was it arbitrarily chosen? Step 3 is even more problematic. The authors state they construct the PDF of each data cluster and then use a "skill score" to select the optimal distribution type from a set of candidates (e.g., normal, Weibull). The skill score formula presented in equation 11 is not a standard goodness-of-fit measure and is poorly explained. The term P_i represents the original data, but the comparison with γ_l, the PDF of a distribution type, is nonsensical—a probability density value cannot be directly compared to a data point in this manner. The skill score calculation appears mathematically incoherent. The authors must provide a clear, step-by-step mathematical definition of their skill score, explaining how it quantifies the fit between a continuous probability density function and a discrete set of data points.

6. The manuscript contains a fundamental error in the interpretation of the marginal probability density functions used within the copula framework. The authors state they treat the "18 features of the target gene... as multiple random variables" and estimate their marginal PDFs using a GMM. However, in a standard copula model for prediction, the variables are not the raw features themselves. The correct procedure is to model the conditional distribution of the target variable (e.g., the binary label of positive/negative interaction) given the features, or to model the joint distribution of the features conditioned on the class label. By modeling the raw features directly as T₁...T_d and plugging them into a vine copula, the authors are implicitly building a model of the feature distribution, not a predictive model for the target gene. This is a category error. The authors must completely re-frame their probabilistic model. They need to clarify whether they are building two separate copula models—one for the feature distribution of positive samples and one for negative samples—and then using Bayes' rule to derive a prediction, or if they have employed a different conditional copula approach. As it stands, the methodological description conflates feature distribution modeling with predictive modeling.

7. The authors' critique of existing methods as "deterministic" and the positioning of their work as a "probabilistic prediction" model is overstated and misrepresents the field. Many machine learning models, including the DNN and XGBoost models they cite, provide probabilistic outputs. For instance, a neural network with a softmax output layer produces a probability distribution over classes. XGBoost can output calibrated probabilities. The novelty, therefore, is not in producing a probability per se, but in the specific architecture of using a vine copula to model feature dependencies for this purpose. The authors must moderate their claims and reframe their contribution accurately. They should state that they are introducing a novel probabilistic architecture based on generative modeling through copulas, rather than implying that all existing models only output a hard binary classification without an associated confidence or probability score. This distinction is crucial for an honest positioning of their work within the existing literature.

8. The handling of the imbalance between positive (831) and negative (306) samples is methodologically weak. The authors use HD-MTD to generate virtual negative samples to balance the dataset. However, they fail to specify the target ratio after expansion. Do they generate negatives until a 1:1 ratio is achieved? Furthermore, the validation of this approach in Table 3 is flawed. When evaluating on 30% of the data, they use a smaller subset of the original, imbalanced data, expand it, and then test on... what? The test set is not described. To properly evaluate the data expansion technique, the authors must perform cross-validation *after* data expansion in a way that prevents data leakage. The standard approach is to expand the training fold only and keep the test fold pristine with original, non-synthetic samples. The manuscript gives no indication that this was done. The authors must describe their exact cross-validation setup for the experiments in Table 3, explicitly stating how they prevented synthetic samples from contaminating the test sets. Without this, the reported performance metrics are likely optimistically biased and meaningless.

9. The description of the 18 selected features in Table 1 is inconsistent, incomplete, and in one case, appears to contain a critical typo that obscures the feature's definition. Features like Rgs_energy (x3), Acc_energy (x4), and Rgt_energy (x5) are all described as "ΔG" values calculated by RNAfold, but no distinction is made between these three thermodynamic features. What is the biological or structural difference between them? The authors must provide a clear definition for each. More alarmingly, in the table, both Sm_7mer_m8 (x7) and Sm_7mer_A1 (x9) are given identical conditional definitions: "z2 - z8 complementary pairing with target g". This cannot be correct. Sm_7mer_A1 typically refers to a match opposite the first nucleotide of the miRNA, which is often an adenine. The definition is almost certainly wrong and must be corrected immediately, as this is a standard and important feature in miRNA targeting. The authors must review the entire table for accuracy and provide complete, unambiguous definitions for every feature.

10. The "Performance test of GMM" section and the accompanying Figure 1 are entirely uninterpretable and must be removed or completely re-conceived. The authors state they randomly selected five miRNAs and show the prediction results of different models, claiming the GMM peak is "closer to the actual target gene." However, for a binary classification task (target or not), a probability density function over a continuous variable does not have a concept of an "actual target gene." What is being plotted on the x-axis? It appears the authors are plotting the probability density of the decision function value S (from equation 17) for the five miRNAs. This is meaningless. A single miRNA has a fixed feature vector, which yields a single, deterministic value of S. Plotting a density around it is nonsensical. This figure reveals a deep misunderstanding of the model's own output. The GMM and copula are used to build a joint probability model; the prediction for a single instance should be a conditional probability, not a full density. This section and its corresponding figure demonstrate a fundamental flaw in how the authors conceptualize their own probabilistic prediction and must be completely omitted from the manuscript.

Reviewer #2: This paper proposes a probabilistic framework for binary prediction that combines Gaussian mixture modeling, R-vine copula–based dependency modeling, and synthetic sample generation to address data imbalance and complex feature dependencies. The method constructs a joint probability distribution to model interactions among multiple variables and derives deterministic predictions from probabilistic outputs, which are then evaluated using standard classification metrics. Experimental results suggest potential performance improvements over several baseline models, though the study primarily demonstrates methodological feasibility rather than comprehensive real-world validation.

While the manuscript presents an interesting idea and a potentially useful methodological framework, there are several substantial issues that prevent acceptance in its current form.

1. For example, several sentences contain awkward phrasing and inconsistent terminology (e.g., switching between “target gene” vs. “target-gene,” and occasionally unclear antecedents), which reduces readability and precision in a methods-heavy paper. These language issues appear throughout the manuscript draft and should be corrected for a PLOS Computational Biology Research Article standard.

2. In Eq. (2), the normalizing constant is written as (1/\sqrt{(2\pi)^m\sigma_m^2}), which is dimensionally unclear for a (seemingly) 1-D Gaussian and mixes “(m)” (component index) with dimensionality; additionally, the text alternates between “k-th” and “m-th” sub-model when defining (\mu_m,\sigma_m^2,\gamma_m), which can confuse the meaning of parameters and prevents a clean step-by-step derivation from Eqs. (1)–(2).

3. In the vine structure definition, Eq. (5) is written in a way that is not mathematically coherent (“({y(k), z(k) = y(k), z(k)|M(k)})”), and Eq. (7) presents nested products with unclear index ranges and undefined symbols (e.g., the meaning of (\prod f_d(T_d)) and the product limits “(i=d-1)” / “(i=i)” are not readable as written), making it hard to verify correctness or reproduce the joint density construction.

4. In the Abstract/summary narrative, the manuscript suggests selecting the “highest probability density” as the deterministic outcome, whereas later it states that the “0.5 quantile (median)” is selected as the deterministic prediction result; these are different decision rules and will generally yield different classifications, so the authors must unify the definition and explain precisely how probabilities are converted into labels.

5. The paper reports Accuracy/Precision/Recall/F1/AUC (Eqs. 12–16) but does not clearly state the train/validation/test split strategy, whether k-fold CV (or LOOCV) is used, or how hyperparameters (e.g., GMM components, vine selection, K-means cluster number, SVM penalty (C)) are tuned; moreover, because synthetic samples are generated (HD–MTD), the manuscript must clarify whether virtual samples are generated **only from the training fold** to avoid information leakage into evaluation.

6. The main computational baselines are simplified distributional variants (e.g., WD, SGM, and GMM variants) rather than recognized state-of-the-art miRNA target prediction systems or recent ML/DL baselines, and the biological validation focuses on essentially one exemplar (miR-8485 → JAK2) rather than a systematic validation of predictions across many targets; thus, the paper needs stronger benchmarking against modern methods and more robust external validation to support generalizability.

7. The authors are encouraged to cite and discuss recent related studies (10.1016/j.csbj.2024.06.032; 10.1093/bib/bbac384; 10.1109/TCBBIO.2025.3610881; 10.1002/advs.202512453; 10.1109/JBHI.2024.3383591), which represent state-of-the-art methodological advances relevant to this topic. Incorporating insights, benchmarking strategies, or methodological elements from these references in future work could strengthen the novelty, validation rigor, and alignment with current research trends.

Reviewer #3: The manuscript presents an interesting attempt to develop a probabilistic framework for microRNA target gene prediction by incorporating feature dependency modeling and a data expansion strategy. Although the study addresses an important problem and proposes a technically sophisticated methodology, several methodological and experimental aspects require further clarification and improvement to adequately support the claims and demonstrate the robustness and general applicability of the proposed approach.

- The novelty and methodological contribution of the proposed model are not clearly justified relative to existing machine learning and deep learning approaches for miRNA target prediction, and the manuscript lacks a rigorous comparison with state of the art computational models such as modern ensemble methods, graph based models, or deep learning frameworks commonly used in this domain.

- The dataset used for training and evaluation is relatively small and highly imbalanced with a substantially larger number of positive samples than negative samples, raising concerns about model generalization and potential overfitting, particularly given the complexity of the probabilistic modeling framework.

- The proposed hybrid distribution mega trend diffusion data expansion technique generates synthetic samples, yet the manuscript does not sufficiently analyze the potential bias introduced by synthetic data nor demonstrate that the generated samples preserve biologically meaningful distributions.

- The feature selection process based on the Pareto principle and feature effectiveness metric lacks a clear biological justification and may risk discarding biologically relevant features, especially given that only a small subset of features was ultimately selected from the originally extracted features.

- The probabilistic modeling framework combining Gaussian mixture models and regular vine copulas is mathematically complex but the manuscript does not provide sufficient explanation of model training procedures, parameter estimation strategies, or computational complexity, limiting reproducibility.

- The comparison experiments include only a limited set of baseline models that are relatively weak and do not reflect the current state of the art in miRNA target prediction methods.

- The evaluation metrics focus mainly on accuracy based measures such as F1 score, accuracy, specificity, and AUC, but the manuscript does not provide statistical significance testing or confidence intervals to demonstrate that the improvements over baseline methods are meaningful.

- The claim that the model performs well using only a small portion of the original dataset is not sufficiently supported by detailed experimental design or statistical validation, and the experimental setup for reduced data scenarios is not clearly described.

- The biological validation experiments focus only on a single predicted interaction between one miRNA and one target gene, which is insufficient to demonstrate the general predictive capability of the model across diverse miRNA target interactions.

**********

**Have the authors made all data and (if applicable) computational code underlying the findings in their manuscript fully available?**

The PLOS Data policy requires authors to make all data and code underlying the findings described in their manuscript fully available without restriction, with rare exception (please refer to the Data Availability Statement in the manuscript PDF file). The data and code should be provided as part of the manuscript or its supporting information, or deposited to a public repository. For example, in addition to summary statistics, the data points behind means, medians and variance measures should be available. If there are restrictions on publicly sharing data or code —e.g. participant privacy or use of data from a third party—those must be specified.

Reviewer #1: None

Reviewer #2: None

Reviewer #3: Yes

**********

PLOS authors have the option to publish the peer review history of their article (what does this mean?). If published, this will include your full peer review and any attached files.

If you choose “no”, your identity will remain anonymous but your review may still be made public.

**Do you want your identity to be public for this peer review?**   For information about this choice, including consent withdrawal, please see our Privacy Policy.

Reviewer #1: **Yes:**  Mohammad Hossein Alizadeh Roknabadi

Reviewer #2: **Yes:**  Bowei Zhao

Reviewer #3: No

[NOTE: If reviewer comments were submitted as an attachment file, they will be attached to this email and accessible via the submission site. Please log into your account, locate the manuscript record, and check for the action link "View Attachments". If this link does not appear, there are no attachment files.]

**Figure resubmission:**

While revising your submission, we strongly recommend that you use PLOS’s NAAS tool (https://ngplosjournals.pagemajik.ai/artanalysis) to test your figure files. NAAS can convert your figure files to the TIFF file type and meet basic requirements (such as print size, resolution), or provide you with a report on issues that do not meet our requirements and that NAAS cannot fix.

After uploading your figures to PLOS’s NAAS tool - https://ngplosjournals.pagemajik.ai/artanalysis, NAAS will process the files provided and display the results in the "Uploaded Files" section of the page as the processing is complete. If the uploaded figures meet our requirements (or NAAS is able to fix the files to meet our requirements), the figure will be marked as "fixed" above. If NAAS is unable to fix the files, a red "failed" label will appear above. When NAAS has confirmed that the figure files meet our requirements, please download the file via the download option, and include these NAAS processed figure files when submitting your revised manuscript.

**Reproducibility:**

To enhance the reproducibility of your results, we recommend that authors of applicable studies deposit laboratory protocols in protocols.io, where a protocol can be assigned its own identifier (DOI) such that it can be cited independently in the future. Additionally, PLOS ONE offers an option to publish peer-reviewed clinical study protocols. Read more information on sharing protocols at https://plos.org/protocols?utm_medium=editorial-email&utm_source=authorletters&utm_campaign=protocols

# Author comment: Author response to Decision Letter 1

**DOI:** 10.1371/journal.pcbi.1014402.r002

1 Apr 2026

Attachment Submitted filename: Response_to_Reviewers (1)R1.docx

# Peer review: Decision Letter 1

**DOI:** 10.1371/journal.pcbi.1014402.r003

**Authors:** Ilya Ioshikhes, Lun Hu

11 May 2026

PCOMPBIOL-D-26-00186R1

MicroRNA target gene prediction model based on input-feature dependency and sample data expansion technique

PLOS Computational Biology

Dear Dr. Dong,

Thank you for submitting your manuscript to PLOS Computational Biology. After careful consideration, we feel that it has merit but does not fully meet PLOS Computational Biology's publication criteria as it currently stands. Therefore, we invite you to submit a revised version of the manuscript that addresses the points raised during the review process.

Please submit your revised manuscript by Jul 11 2026 11:59PM. If you will need more time than this to complete your revisions, please reply to this message or contact the journal office at ploscompbiol@plos.org. When you're ready to submit your revision, log on to https://www.editorialmanager.com/pcompbiol/ and select the 'Submissions Needing Revision' folder to locate your manuscript file.

Please include the following items when submitting your revised manuscript:

* A letter that responds to each point raised by the editor and reviewer(s). You should upload this letter as a separate file labeled 'Response to Reviewers'. This file does not need to include responses to formatting updates and technical items listed in the 'Journal Requirements' section below.

* A marked-up copy of your manuscript that highlights changes made to the original version. You should upload this as a separate file labeled 'Revised Manuscript with Track Changes'.

* An unmarked version of your revised paper without tracked changes. You should upload this as a separate file labeled 'Manuscript'.

If you would like to make changes to your financial disclosure, competing interests statement, or data availability statement, please make these updates within the submission form at the time of resubmission. Guidelines for resubmitting your figure files are available below the reviewer comments at the end of this letter.

As the corresponding author, your ORCID iD is verified in the submission system and will appear in the published article. PLOS supports the use of ORCID, and we encourage all coauthors to register for an ORCID iD and use it as well. Please encourage your coauthors to verify their ORCID iD within the submission system before final acceptance, as unverified ORCID iDs will not appear in the published article. Only the individual author can complete the verification step; PLOS staff cannot verify ORCID iDs on behalf of authors.

We look forward to receiving your revised manuscript.

Kind regards,

Lun Hu

Academic Editor

PLOS Computational Biology

Ilya Ioshikhes

Section Editor

PLOS Computational Biology

**Additional Editor Comments:**

According to reviewers' comments, the manuscript has been significantly improved. However, one of our reviewers still had several minor concerns, such as the training strategy of baselines, the biological validity of synthetic data, the biological justification of feature selection, and lack of statistic details. Due to these issues, I would like ask authors to revise their manuscript for a minor revision.

**Journal Requirements:**

If the reviewer comments include a recommendation to cite specific previously published works, please review and evaluate these publications to determine whether they are relevant and should be cited. There is no requirement to cite these works unless the editor has indicated otherwise.

1) Your manuscript's sections are not in the correct order.  Please amend to the following order: Abstract, Introduction, Results, Discussion, and Methods

2) Thank you for including an Ethics Statement for your study. Please include:

i) A statement that formal consent was obtained (must state whether verbal/written) OR the reason consent was not obtained (e.g. anonymity). NOTE: If child participants, the statement must declare that formal consent was obtained from the parent/guardian.].

3) We have noticed that you have uploaded Supporting Information files, but you have not included a complete list of legends. Please add a full list of legends for your Supporting Information files after the references list.

4) Some material included in your submission may be copyrighted. According to PLOS’s copyright policy, authors who use figures or other material (e.g., graphics, clipart, maps) from another author or copyright holder must demonstrate or obtain permission to publish this material under the Creative Commons Attribution 4.0 International (CC BY 4.0) License used by PLOS journals. Please closely review the details of PLOS’s copyright requirements here: PLOS Licenses and Copyright. If you need to request permissions from a copyright holder, you may use PLOS's Copyright Content Permission form.

Please respond directly to this email and provide any known details concerning your material's license terms and permissions required for reuse, even if you have not yet obtained copyright permissions or are unsure of your material's copyright compatibility. Once you have responded and addressed all other outstanding technical requirements, you may resubmit your manuscript within Editorial Manager.

Potential Copyright Issues:

i) The following Figure contains a logo or branding: DL101-Dual-Luciferase-Reporter-Assay-Kit1534845724946. We are not permitted to publish this under our CC-BY 4.0 license, even with permission. We ask that you please remove or replace it.

5) In the online submission form, you indicated that  available from the corresponding author on reasonable request.. All PLOS journals now require all data underlying the findings described in their manuscript to be freely available to other researchers, either

1. In a public repository

2. Within the manuscript itself

3. Uploaded as supplementary information.

This policy applies to all data except where public deposition would breach compliance with the protocol approved by your research ethics board. If your data cannot be made publicly available for ethical or legal reasons (e.g., public availability would compromise patient privacy), please explain your reasons by return email and your exemption request will be escalated to the editor for approval. Your exemption request will be handled independently and will not hold up the peer review process, but will need to be resolved should your manuscript be accepted for publication. One of the Editorial team will then be in touch if there are any issues.

**Reviewers' comments:**

Reviewer's Responses to Questions

**Comments to the Authors:**

**Please note here if the review is uploaded as an attachment.**

Reviewer #1: accept

Reviewer #2: The draft submitted by the author gave a satisfactory answer. I think this article is acceptable.

Reviewer #3: The revised manuscript has improved substantially compared with the previous version. The authors have made meaningful efforts to clarify the probabilistic modeling framework, address the data leakage concern, improve the description of feature selection, add stronger baseline comparisons, include confidence intervals and statistical testing, and reframe the biological validation as a proof-of-concept rather than as comprehensive validation of the model. These revisions improve the methodological transparency and reduce several concerns raised in the previous review. However, some issues remain that should be addressed before the manuscript can be considered fully satisfactory.

The comparison with state-of-the-art models has improved but remains insufficiently documented. The authors added comparisons with miRBench-CNN and Hybrid AE-CNN, which is a useful improvement. However, the manuscript should more clearly state whether these models were retrained on the same training/test splits, whether the same features and preprocessing were used, and how their hyperparameters were selected. If the reported results are taken from prior studies rather than reproduced under the same experimental setting, the comparison may not be fully fair. Approximate values such as “F1 ≈ 0.82” and “AUC ≈ 0.84” should also be avoided unless the exact source and evaluation conditions are clearly stated. The manuscript should provide enough detail to ensure that the benchmark comparison is reproducible and comparable.

The synthetic data expansion method is better described, but the biological validity of generated samples is still not fully demonstrated. The authors now clarify that HD-MTD is applied only to the training set and that the test set remains free of synthetic samples, which addresses a major methodological concern. However, the manuscript still does not sufficiently evaluate whether the generated samples preserve biologically meaningful feature distributions. Because synthetic negative samples directly influence model training, the authors should provide additional validation of the augmented data, such as distributional comparisons between real and synthetic samples, feature correlation preservation, KS tests, MMD analysis, PCA/UMAP visualization, or comparison of biological feature constraints before and after augmentation. Improved predictive performance alone does not fully rule out synthetic data bias.

The reduced-data claim is improved but should be described more precisely. The revised manuscript reports repeated experiments and confidence intervals, which strengthens the claim that the model performs well with limited training data. However, the text should be more precise about whether “30% of the original dataset” refers to 30% of the entire dataset or 30% of the training partition. In some sections, the wording appears to refer to the original dataset, while the experimental design indicates that the training set was subsampled. This distinction matters and should be made consistent throughout the Abstract, Author Summary, Results, and Discussion.

The feature selection procedure is more transparent, but the biological justification remains limited. The removal of the Pareto principle and the clarification of the LinearSVC-based feature-ranking procedure are positive changes. However, the biological rationale for the final selected feature set remains somewhat limited. The manuscript should include either a supplementary table listing all 90 original features and their rankings or a clearer explanation of why the selected 18 features are biologically meaningful. The authors should also provide the G-means curve or supporting evidence showing the performance plateau at 18 features.

The experimental validation is now properly framed, but the claims should remain cautious throughout the manuscript. The authors appropriately reframed the miR-8485–JAK2 validation as a proof-of-concept case study, which is a reasonable response. However, the manuscript should avoid language implying broad biological validation of the full model. Phrases such as “confirmed biological relevance of the model’s predictions” should be softened to “confirmed the biological relevance of one model-generated prediction.” This distinction is important because only one predicted interaction was experimentally validated.

The HD-MTD clustering procedure needs additional detail. The authors state that the elbow method selected M = 3 clusters. They should clarify whether M = 3 was selected globally once or independently within each training fold. If selected globally using the full dataset, that could still introduce information leakage. If selected within each fold, the manuscript should report whether M was consistently equal to 3 across folds and random seeds.

The statistical testing is a useful addition, but more details are needed. The paired bootstrap testing and confidence intervals strengthen the evaluation. However, the manuscript should clarify what was resampled: individual test instances, repeated runs, or paired predictions from models. The authors should also clarify whether multiple-comparison correction was applied when comparing the proposed model against multiple baselines.

**********

**Have the authors made all data and (if applicable) computational code underlying the findings in their manuscript fully available?**

The PLOS Data policy requires authors to make all data and code underlying the findings described in their manuscript fully available without restriction, with rare exception (please refer to the Data Availability Statement in the manuscript PDF file). The data and code should be provided as part of the manuscript or its supporting information, or deposited to a public repository. For example, in addition to summary statistics, the data points behind means, medians and variance measures should be available. If there are restrictions on publicly sharing data or code —e.g. participant privacy or use of data from a third party—those must be specified.

Reviewer #1: None

Reviewer #2: None

Reviewer #3: Yes

**********

PLOS authors have the option to publish the peer review history of their article (what does this mean?). If published, this will include your full peer review and any attached files.

If you choose “no”, your identity will remain anonymous but your review may still be made public.

**Do you want your identity to be public for this peer review?**   For information about this choice, including consent withdrawal, please see our Privacy Policy.

Reviewer #1: **Yes:**  Mohammad Hossein Alizadeh Roknabadi

Reviewer #2: No

Reviewer #3: No

**Figure resubmission:**

-->While revising your submission, we strongly recommend that you use PLOS’s NAAS tool (https://ngplosjournals.pagemajik.ai/artanalysis) to test your figure files. NAAS can convert your figure files to the TIFF file type and meet basic requirements (such as print size, resolution), or provide you with a report on issues that do not meet our requirements and that NAAS cannot fix.

After uploading your figures to PLOS’s NAAS tool - https://ngplosjournals.pagemajik.ai/artanalysis, NAAS will process the files provided and display the results in the "Uploaded Files" section of the page as the processing is complete. If the uploaded figures meet our requirements (or NAAS is able to fix the files to meet our requirements), the figure will be marked as "fixed" above. If NAAS is unable to fix the files, a red "failed" label will appear above. When NAAS has confirmed that the figure files meet our requirements, please download the file via the download option, and include these NAAS processed figure files when submitting your revised manuscript.

**Reproducibility:**

To enhance the reproducibility of your results, we recommend that authors of applicable studies deposit laboratory protocols in protocols.io, where a protocol can be assigned its own identifier (DOI) such that it can be cited independently in the future. Additionally, PLOS ONE offers an option to publish peer-reviewed clinical study protocols. Read more information on sharing protocols at https://plos.org/protocols?utm_medium=editorial-email&utm_source=authorletters&utm_campaign=protocols

# Author comment: Author response to Decision Letter 2

**DOI:** 10.1371/journal.pcbi.1014402.r004

21 May 2026

Attachment Submitted filename: Response_to_Reviewers.docx

# Peer review: Decision Letter 2

**DOI:** 10.1371/journal.pcbi.1014402.r005

**Authors:** Ilya Ioshikhes, Lun Hu

3 Jun 2026

Dear Dr Dong,

We are pleased to inform you that your manuscript 'MicroRNA target gene prediction model based on input-feature dependency and sample data expansion technique' has been provisionally accepted for publication in PLOS Computational Biology.

Before your manuscript can be formally accepted you will need to complete some formatting changes, which you will receive in a follow up email. A member of our team will be in touch with a set of requests.

Please note that your manuscript will not be scheduled for publication until you have made the required changes, so a swift response is appreciated.

IMPORTANT: The editorial review process is now complete. PLOS will only permit corrections to spelling, formatting or significant scientific errors from this point onwards. Requests for major changes, or any which affect the scientific understanding of your work, will cause delays to the publication date of your manuscript.

Should you, your institution's press office or the journal office choose to press release your paper, you will automatically be opted out of early publication. We ask that you notify us now if you or your institution is planning to press release the article. All press must be co-ordinated with PLOS.

Thank you again for supporting Open Access publishing; we are looking forward to publishing your work in PLOS Computational Biology.

Best regards,

Lun Hu

Academic Editor

PLOS Computational Biology

Ilya Ioshikhes

Section Editor

PLOS Computational Biology

***********************************************************

All reviewers were satisfied with the changes made in this revised version of the manuscript.

Reviewer's Responses to Questions

**Comments to the Authors:**

**Please note here if the review is uploaded as an attachment.**

Reviewer #3: I have carefully reviewed the second revised version of the manuscript and the authors’ responses to the previous comments. In my opinion, the authors have properly addressed the major concerns raised during the earlier review rounds. The revised manuscript is now much clearer, better organized, and more complete in terms of methodological explanation, presentation of results, and discussion of the study’s limitations.

I appreciate the authors’ efforts in revising the manuscript and responding to the concerns. Based on the current version, I do not have any remaining major academic or technical concerns. The manuscript appears to be suitable for publication.

However, I recommend that the authors perform one final round of careful proofreading before publication. There are still some minor language, grammar, formatting, or wording issues that should be corrected to improve the clarity and readability of the final version. These issues are minor and do not affect the scientific contribution of the manuscript.

**********

**Have the authors made all data and (if applicable) computational code underlying the findings in their manuscript fully available?**

The PLOS Data policy requires authors to make all data and code underlying the findings described in their manuscript fully available without restriction, with rare exception (please refer to the Data Availability Statement in the manuscript PDF file). The data and code should be provided as part of the manuscript or its supporting information, or deposited to a public repository. For example, in addition to summary statistics, the data points behind means, medians and variance measures should be available. If there are restrictions on publicly sharing data or code —e.g. participant privacy or use of data from a third party—those must be specified.

Reviewer #3: Yes

**********

PLOS authors have the option to publish the peer review history of their article (what does this mean?). If published, this will include your full peer review and any attached files.

If you choose “no”, your identity will remain anonymous but your review may still be made public.

**Do you want your identity to be public for this peer review?**   For information about this choice, including consent withdrawal, please see our Privacy Policy.

Reviewer #3: No

# Editor report: Acceptance letter

**DOI:** 10.1371/journal.pcbi.1014402.r006

**Authors:** Ilya Ioshikhes, Lun Hu

PCOMPBIOL-D-26-00186R2

MicroRNA target gene prediction model based on input-feature dependency and sample data expansion technique

Dear Dr Dong,

I am pleased to inform you that your manuscript has been formally accepted for publication in PLOS Computational Biology. Your manuscript is now with our production department and you will be notified of the publication date in due course.

The corresponding author will soon be receiving a typeset proof for review, to ensure errors have not been introduced during production. Please review the PDF proof of your manuscript carefully, as this is the last chance to correct any errors. Please note that major changes, or those which affect the scientific understanding of the work, will likely cause delays to the publication date of your manuscript.

Soon after your final files are uploaded, unless you have opted out, the early version of your manuscript will be published online. The date of the early version will be your article's publication date. The final article will be published to the same URL, and all versions of the paper will be accessible to readers.

For Research, Software, and Methods articles, you will receive an invoice from PLOS for your publication fee after your manuscript has reached the completed accept phase. If you receive an email requesting payment before acceptance or for any other service, this may be a phishing scheme. Learn how to identify phishing emails and protect your accounts at https://explore.plos.org/phishing.

Thank you again for supporting PLOS Computational Biology and open-access publishing. We are looking forward to publishing your work!

With kind regards,

Anita Estes

PLOS Computational Biology | Carlyle House, Carlyle Road, Cambridge CB4 3DN | United Kingdom ploscompbiol@plos.org | Phone +44 (0) 1223-442824 | ploscompbiol.org | @PLOSCompBiol
